# Cross-Language Distributions of High Frequency and Phonetically Similar Cognates

**DOI:** 10.1371/journal.pone.0063006

**Published:** 2013-05-10

**Authors:** Job Schepens, Ton Dijkstra, Franc Grootjen, Walter J. B. van Heuven

**Affiliations:** 1 Donders Institute for Brain, Cognition and Behaviour, Radboud University Nijmegen, Nijmegen, The Netherlands; 2 Centre for Language Studies, Radboud University Nijmegen, Nijmegen, The Netherlands; 3 School of Psychology, University of Nottingham, Nottingham, United Kingdom; University of Leicester, United Kingdom

## Abstract

The coinciding form and meaning similarity of cognates, e.g. ‘flamme’ (French), ‘Flamme’ (German), ‘vlam’ (Dutch), meaning ‘flame’ in English, facilitates learning of additional languages. The cross-language frequency and similarity distributions of cognates vary according to evolutionary change and language contact. We compare frequency and orthographic (O), phonetic (P), and semantic similarity of cognates, automatically identified in semi-complete lexicons of six widely spoken languages. Comparisons of P and O similarity reveal inconsistent mappings in language pairs with deep orthographies. The frequency distributions show that cognate frequency is reduced in less closely related language pairs as compared to more closely related languages (e.g., French-English vs. German-English). These frequency and similarity patterns may support a better understanding of cognate processing in natural and experimental settings. The automatically identified cognates are available in the supplementary materials, including the frequency and similarity measurements.

## Introduction

In contrast to what the story on the Tower of Babel suggests, it is sometimes the case that speakers of different languages can understand each other. For example, speakers of Dutch and German or Spanish and Italian, are able to understand quite a lot of each other’s speech. It is clear that mutual intelligibility depends on the degree of cross-language similarity. Translation equivalents that overlap in form and meaning may provide help in getting a message across the language barrier [1]. In the present paper, we computationally determined the form and meaning overlap and the frequency characteristics of translation equivalents across six languages to compare lexical similarity distributions. Before zooming in on our simulations of cross-language similarity distributions, we will first discuss dimensions of word overlap as well as lexicostatistic and phylogenetic methods currently in use for estimating cross-language lexical similarity.

### Dimensions of Cross-Language Similarity

The cross-language similarity of word pairs from different languages can concern both form and meaning overlap. With respect to word form similarity, one can distinguish orthographic similarity and phonetic similarity. Orthographically (O) similar words are called (near-) homographs, and phonetically (P) similar words are called (near-) homophones. With respect to meaning overlap, semantically (S) similar words are called synonyms within languages and translation equivalents between two languages. In this study, we were particularly interested in words with a relatively high form and meaning overlap. Translation equivalents with large spelling and/or sound similarities across languages are referred to as *cognates*. For example, the English-Dutch translation equivalents *wheel – wiel* have a high spelling and sound overlap. Although cognates are often historically related, we do not use this etymological criterion to identify them in the present study.

In contrast to cognates, other word pairs with a similar spelling and sound refer to different rather than similar concepts. Such word pairs can be referred to as *false friends*. False friends complicate the understanding of a foreign language. For example, the English-Dutch form-similar words *magazine* (English: *warehouse*, *periodical*) and *magazijn* (Dutch: *warehouse*) also have different meaning aspects while their form overlap is high.

As Table 1 shows, S, O, or P similarity can be defined and compared not only within-languages, but also between-languages. Research indicates that the word recognition performance by multilinguals depends on both within- and between-language S, O, and P similarity [2], [3].

**Table 1:** Intralingual and interlingual language similarities in terms of semantics (S), orthography (O), and phonology (P).

Intralingual	English-English word pair	Overlap	English-Dutch word pair	Interlingual
Synonym	*bad – evil*	S	*bad – slecht*	Translation
Homograph	*bow – bow*	O	*type – type*	Homograph
Homophone	*naught – nought*	P	*wheel – wiel*	Homophone
Similar synonym	*eatable – edible*	(O and/or P) and S	*tomato – tomaat*	Cognate
Homonym	*night – knight*	O and/or P, no S	*bloom – bloem*	False friend

Translation equivalents can not only be compared with respect to their linguistic dimensions (O, P, and S), but also with respect to how often the words are encountered or used in everyday language. For example, the Dutch translation equivalent of *hair,* which is written as *haar,* is used much more often than *hair* because *haar* also translates to *her*. Word frequency can be assessed by measuring how often a particular word occurs per million words (occurrence per million or opm) in collected corpora (e.g., [4], [5]). A high word frequency has been found to facilitate within- and between-language word recognition in terms of response times and accuracy (e.g., [6]). Frequency of word usage can be used to distinguish common and uncommon S, O, or P similarities and may serve as a ‘weighting factor’ when assessing their effects. In our study, we will use word frequency statistics to quantify the occurrence of similarities on these S, O, and P dimensions across six European languages, three Germanic languages (English, German, and Dutch), and three Romance languages (French, Italian, and Spanish).

### Existing Quantitative Approaches for Measuring Cross-Language Similarity

To measure cross-language similarity, quantitative approaches are available in various branches of cognitive science and biology [7]. *Lexicostatistical comparison* typically estimates the percentage of shared cognates in language pairs to give an account of the historical relatedness between languages. For example, Germanic languages are more closely related to each other than to Romance languages, and vice versa. In the lexicostatical approach, the percentage of cognates shared by two languages is estimated on the basis of cognacy judgments by experts. The vocabulary used for such cognacy judgments often consists of translation pairs from Swadesh lists [8]. Swadesh lists are small sets of universal culture-free meanings that are robust to changes in meaning and appearance over time. Examples of robust concepts in the Swadesh list are *water, arm,* and *ear*. Meaning of items in Swadesh lists is considered to be resistant to borrowings or chance resemblances between languages. Quantifications of the percentage of shared cognates in Swadesh lists can accurately predict language relatedness [9], [10] and can shed new light on traditional accounts of historical relatedness.

In the *phylogenetic approach*, the likelihood of cognate sets in language trees is maximized to find the language tree that best reflects cognacy between languages. This approach is based on techniques from evolutionary biology and is also applied in studies of language evolution. Divergence in evolutionary relationships can be simulated with phylogenetic techniques using expert cognacy judgements in 200-item Swadesh lists of 87 Indo-European languages [11], also see [12]. Language trees can be used to predict language divergence times and provide more general insights into the evolutionary process. The branch lengths of these phylogenetic language trees are proportional to maximum likelihood estimates of evolutionary change. Cognate classifications in Swadesh lists are made by experts using the comparative method. Pagel [13] found that high frequency words evolve relatively slowly; high frequency words in Swadesh lists are therefore useful for estimations of evolutionary relatedness between languages.

In the phylogenetic studies on language relatedness mentioned above, cognacy judgments are still made by experts using the comparative method. However, recent attempts show that interchanging expert cognacy judgments for an *automatic cognacy measure* can result in accurate predictions of language relatedness as well [14]–[16]. As a consequence, computational methods are becoming more and more popular to estimate the numbers of shared cognates across languages [15], [17]–[20].

An example of an automatic measure that is able to simulate lexical matching criteria is the *Levenshtein matching algorithm*. This algorithm is a standard string matching metric from information theory that calculates the minimum number of insertions, deletions, and substitutions that are needed to edit one string into another. For example, the Levenshtein distance of the cognate pair *guitar* – *gitaar* results in a distance of two (one deletion of *u* and one insertion of *a*). When applied to words, this number represents form distance based on the overlap of the letters in the two words. Recent studies have made successful use of Levenshtein distance to simulate orthographic similarity [16], [21], [22].

Yarkoni et al. [22] showed that the Levenshtein distance is able to outperform Coltheart’s orthographic neighbourhood size metric [23] in terms of word recognition and word production measures (a neighbour is a word that differs in just one letter position from a target word, e.g., *cork* – *work*). The authors computed the so-called orthographic Levenshtein distance 20 (OLD20) for all words in a monolingual lexicon (including words of different lengths). This OLD20 measures the average distance over the 20 closest neighbours according to the Levenshtein distance metric. OLD20 turned out to be a significantly better predictor of both lexical decision and pronunciation performance in three large data sets than standard orthographic neighbourhood density. There was a stronger interaction of the new measure with word frequency and stronger effects of neighbourhood frequency as well. However, due to its dependency on a fixed set of 20 words, OLD20 may conflate neighbourhood density with word frequency.

### A New Computational Approach for Measuring Cross-Language Similarity

In a previous study [24], Schepens et al. constructed a language similarity ordering by automatically comparing the semi-complete lexicons of six European languages. The method used was similar to those used in lexicostatistical studies, but expert cognacy judgments were replaced with automatic judgement for semantic and orthographic similarity. To determine *semantic similarity* across languages, translation equivalents from six European languages were collected using a professional translation system. The word pairs that were identified using automatic translation overlapped substantially (81.5%) with subjective translation judgments [25]. *Orthographic similarity* was determined by applying a formal cognacy measure assuming semantic similarity of translation pairs. It was found that normalized scores of a Levenshtein distance based measure resembled form similarity judgments to a large extent (91%). This implies that researchers selecting cognates (e.g., as stimulus materials in experiments) can be confident in using computational tools for determining similarity automatically.

The numbers of automatically identified cognates correlated significantly (r = 0.72, p<.001) with branch lengths extracted from a study by Gray and Atkinson [11]. Although both accounts were largely consistent, some differences were observed, which appeared to be due to the similarity of English to Romance languages. The most prominent differences between the two studies were found in their accounts of English-French, English-Spanish, and English-Italian relatedness. A possible explanation is that the total English lexicon contains about 50% borrowings from Romance languages [26]. Differences between the results from expert and computational approaches may be explained by differences in word frequency of cognates, phonetic similarity and the mapping of phonetic to orthographic similarity. The present study considers these theoretically important but unresolved issues.

First, Schepens et al. [24] demonstrated that the degree of lexical orthographic similarity between language pairs could be quantified in terms of cognate distributions within and between languages from the same or different families. However, the present study also takes into account that cognates and translation equivalents have varying *frequencies of usage* in the languages concerned.

Second, the present study considers language pairs in terms of the *phonetic similarity* of translation equivalents in these languages. It will be investigated how a measure of phoneme similarity can contribute to the Levenshtein distance. Assessing cross-language phonetic similarity requires a phonetic representation of words in the different languages with a cross-linguistically valid measurement system. Therefore, an adapted International Phonetic Alphabet (IPA) will be used for the cross-language comparisons. The use of this categorization system allows the assessment of phonetic differences that do not directly depend on phonological combinations present in the languages considered. Phonetic representations of words are available in lexical databases [4], [27]–[29]. Vitevich [30] proposed that the Levenshtein distance metric applied to phonetic representations of translation equivalents could be used for estimations of phonological overlap between languages.

Third, the relationship between orthographic and phonetic similarity of translation equivalents in various language pairs will be considered in the present study. It is likely that the derived orthographic and phonetic similarity measures will be correlated; their relationship must be complex, because it depends on the *orthographic depth* or shallowness of the two spelling systems that the compared languages employ. Orthographic depth is a key term with respect to the orthographic make-up of languages. In the case of *father,* the English form has one letter more (the *h*) than the Dutch and German forms. This is an example of a word for which the English orthographic depth is *deeper* than the orthographic depth in Dutch and German. In this case, the orthographic depth of Dutch and German is shallower. In English, the two-letter combination *th* stands for the single phoneme indicated by θ. The number of phonemes in the word *father* is therefore the same across Dutch, German, and English. Also, English has a deeper orthography in which it can pronounce the 4-letter combination *-ough* in at least six different ways depending on the preceding letter: *bough*, *cough*, *dough*, *rough*, *tough*, *though*. This single 4-letter combination maps out many different sounds. In a perfectly shallow orthography, n-letter combinations always map to one sound.

The degree to which languages have a shallow or deep orthographic depth can be quantified using computational tools [31]–[34]. According to [34], orthographic depth may be related to differences between O and P similarity measures. Only shallow orthographies (e.g. German and Spanish) showed high overlap of computationally derived similarity measures and deep orthographies showed low overlap (e.g. French). We hypothesize that the variation between shallow and deep spelling systems has consequences for the orthographic and phonological dimensions of cross-language similarity distributions. Two more specific hypotheses are concerned with orthographic depth and cognate frequency.

#### Orthographic depth

Quantifying the mapping between phonological and orthographic dimensions allows us to measure the orthographic depth of the spelling systems. We assume that differences in orthographic depth directly affect the similarity between spelling systems, because spelling systems tend to be parasitic on speech systems [35]. Our expectation is that distributions of phonetically similar cognates are associated with different patterns in their orthographic similarity distributions, depending on the mapping processes that determine orthographic depth. Because of their large degree of form similarity, differences in highly similar cognates across language combinations might reflect changes in mapping processes in a more sensitive way than differences in translation equivalents in general. The resulting quantifications of orthographic depth in terms of cognate frequency distributions are compared to commonly used categorizations of orthographic depth (in terms of regularities in spelling to sound mapping).

#### Cognate frequency

In addition, we will investigate how differences in word frequency interact with differences in orthographic depth. O and P similarity distributions as well as word frequency distributions vary within the same linguistic system. The differences between how often we write and pronounce words may have consequences for the shapes of the orthographic as well as the phonetic similarity distribution. We assume that frequency of use and stability go hand in hand: Words with a more frequent use are generally more stable, while less frequent words are more susceptible to lexical replacement [36]. More and less closely related languages will therefore show different shapes in their word frequency distributions. We expect that words shared between more closely related languages are used more often than words shared between less closely related languages. Thus, differences in cognate frequency distributions should be directly related to the degree of relatedness between languages. It is predicted that cognates of higher frequency occur more in combinations of more closely related languages.

## Method

An automatic cognate identification procedure was used that involved an application of the Levenshtein distance (discussed in the Introduction) to lexical databases of six languages (Dutch, English, German, French, Spanish, Italian), linking each word semantically by means of a translation database. The lexical and translation data used and the new automatic cognate identification procedures for orthographic and phonetic similarity are described below.

### Integrated Database

First, we incorporated lexical databases for each of the six languages in our computational tools in order to compare language similarity across languages. We used the standard input-output functionality of Euroglot Professional [37] as a translation database, and restricted ourselves to the first translation provided for each input word. Word matching between the lexicons and the dictionary resulted in an average of 3449.8 different highly frequent words per language with frequency, semantic (S), orthographic (O), and phonetic (P) information available (*SD* = 1076.3), see second column of Table 2. The availability of word frequency estimations enabled us to apply a threshold on the frequency of translation equivalents to be selected. We adopted a minimum frequency threshold of 10 occurrences per million words. This is a relatively conservative boundary that maintains the selection of a substantial part of items used in daily conversation. The relatively large English lexicon is known to contain both Germanic and Romance words, which is reflected in the numbers of translation equivalents in Table 2. All languages have most translation equivalents with English, presumably because English often has both a translation of Romance origin and a translation of Germanic origin. For example, the nouns *assassin* and *murderer* can both translate to French as *assassin* and to Dutch as *moordenaar*.

**Table 2:** Calculations of relative cognate frequency distinguish closely related language pair from less closely related language pairs.

Language Pair	Translation Equivalents (F)	Cognates (F, O)	Cognates (F, P)	Relative Cognate Frequency (F, O, P)
Spanish-Italian	2946	*(2)* 1438	996	(1) 1.04
Dutch-English	4192	1104	*(2)* 1223	(2).94
Dutch-German	2802	*(1)* 1474	*(1)* 1640	(3).89
Italian-French	2846	1128	713	.89
Spanish-French	2761	1166	849	.82
German-English	4625	778	953	.76
Dutch-Italian	2893	491	525	.69
Dutch-Spanish	2527	481	509	.65
Spanish-German	2632	418	461	.61
French-English	*(2)* 5206	*(3)* 1272	*(3)* 1058	.57
Spanish-English	*(3*) 5057	1057	869	.57
Dutch-French	2599	559	639	.56
Italian-English	*(1)* 5281	962	572	.47
Italian-German	2835	405	437	.44
French-German	2545	452	448	.40

Besides relative cognate frequency, also number of translation equivalents and number of identified cognates are given. The abbreviations in each of the column headers show the thresholds applied, namely: frequency (F) of at least 10 occurrences per million, orthographic (O) similarity of at least.5 (0 = no overlap, 1 = identical), phonetic (P) similarity of at least.75 (0 = no overlap, 1 = identical). The table is sorted by cognate frequency relative to translation equivalent frequency (.5 = cognates have half the frequency of translation equivalents, 1 = equal frequency, 2 = double frequency).

In order to assess whether translation equivalents from automatic translation are similar to translation equivalents from subjective similarity judgments, automatically identified translation pairs for Dutch-English were compared with semantic similarity ratings for 1004 semantic relations [25]. The results showed that 691 out of 701 (99%) automatically identified translation pairs had high (rated 5/7 or higher) semantic similarity ratings, 6 (0%) had lower semantic similarity ratings. Furthermore, 776 out of 951 (81.5%) high rated word pairs were found in the translation database used. The differences in database retrieval and ratings appear to originate from the specific conceptual structure implemented in the database, which was constructed by experts, see [24]. For instance, a translation pair like *gemeen* – *cruel* is absent in the database, because, according to experts, it does not share the exact same relation(s) to the shared concept. Other word pairs in the database, like *gemeen – mean* and *wreed – cruel*, are, in fact, better translation pairs than those obtained by the semantic similarity ratings. Overall, we conclude that automatic translation can be used successfully to classify translation pairs as potential cognates.

### Orthographic Similarity

We examined word pairs across language combinations in terms of their frequency and O and P similarity. The O- and P-similarity measures were validated using subjective similarity norms.

Orthographic similarity is known to influence performance in tasks that require word naming, picture naming, lexical decision, and multilingual tasks like translation naming (as an example, consider [2]). Because of a limited availability of orthographic similarity measures, researchers need to collect orthographic similarity ratings to select their stimuli or use form-identical items only. Orthographic similarity norms are available [25] for items with various degrees of orthographic similarity. Such norms are based on experimentally acquired ratings for a variety of lexical items in order to capture the continuous nature of orthographic similarity. Schepens et al. [24] demonstrated that computational orthographic similarity measures can successfully simulate orthographic similarity norms based on experimentally acquired ratings. Recently, a number of computational dialectometry, quantitative historical linguistic, and psycholinguistic studies have been using the Levenshtein distance metric [38] (see definition in the Introduction), to calculate similarity between words [16], [18], [22], [24], [39].

Various adjustments to the standard Levenshtein distance have been proposed to improve the measure. Yarkoni et al. [22] tested transposition of letters (e.g., ‘trial’ into ‘trail’), but this resulted in virtually identical similarity scores. Also, varying substitution costs (systematic 20% reductions or increases in relative cost of insertion, deletion, or substitution) did produce similarly unaffected results. However, this aspect needs to be evaluated using a reasonable substitution cost distribution.

Because the Levenshtein distance metric depends strongly on word length, it needs to be normalized in order to compare orthographic similarity scores between long and short words. Normalization can be performed as in Equation 1 below. This normalized Levenshtein Distance (NLD) makes sure that identical form-overlap between translation equivalents results in similarity scores of one, and no overlap results in a score of zero. Various slightly different ways to normalize the Levenshtein distance have been utilized, see Pompei et al. [40] for a comparison. Mackay and Kondrak [17] have argued that the Levenshtein distance needs to be normalized in an exponential instead of in a linear way. Vanilla Levenshtein distance introduces a bias to distances between classes of words that adopt regular n-gram patterns. For example, the infinitive of Dutch verbs usually ends with the suffix *-en*. Normalizing the Levenshtein distance by the maximum word length of the two compared words can account for such issues.
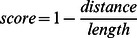



Equation 1. Levenshtein distance normalized for word length. *Length* is the maximum of the source expression and the destination expression. *Distance* is the minimum number of insertions, deletions, and substitutions.

### Phonetic Similarity

Phonetic similarity can also be measured with the Levenshtein distance. For this measure, we varied substitution costs according to similarities between the phonemes in the two phonetic representations of words.

In addition to orthographic similarity, phonetic similarity is one of the keys for identifying cognates [17]. Phonetic similarity is concerned with articulatory, acoustic, and perceptual similarities between vowels and consonants. Kondrak [41], [42] developed the ALINE software for gradual phonetic similarity measurement, in which phonemes are represented as vectors with phonetic features. Differences between 10 binary features and 2 multi-valued features of two phonemes were multiplied with each feature’s salience weight. Subsequently, they were summed up, normalized by dividing by maximum word length, and subtracted from a maximum score to finally result in a phoneme similarity score between 0 and 1. The two multi-valued features were *manner* and *place. Manner* could take 7 values: stop = 1.0, affricate = 0.9, fricative = 0.8, approximant = 0.6, high vowel = 0.4, mid vowel = 0.2, and low vowel = 0.0 (as based on Ladefoged [43]). Kondrak [41] was able to demonstrate that his gradual measure of segment distance outperforms binary measures. McMahon and McMahon [10] developed a similar method in which segment distance measurements are based simply on the number of overlapping phonetic features. The measure was considered successful and further research into gradual segment distance measurement was encouraged. At the same time, Heeringa [16] developed a similar measure but this did not lead to better performance. Heeringa et al. [44] concluded that simple phonetic transcriptions (as yet still) perform better than phonetic feature representations.

To determine a degree of phonetic similarity, a computer algorithm needs phonetic representations. Lexical databases can provide researchers with phonetic transcriptions of word pronunciation, all in varying phonetic alphabets, which are suitable for cross-language comparison between translation equivalents. By re-coding each transcription, using symbols from a universally applicable phonetic alphabet, such as the International Phonetic Alphabet (IPA) [45], it is possible to compare pronunciation irrespective of a particular phonetic system. Although the IPA is being improved continuously, the alphabet in its current form is a useful symbolic representation of speech. For computer applications, the IPA is often simplified into a single coded set of phonemes, varying in components and complexity.

For the phonetic comparison across six languages in the present study, the IPA symbols were re-coded into an ASCI-coded phonetic alphabet. For Dutch, German, and English, we used DISC phonetic transcriptions from Celex [4]. DISC is an IPA-based coding scheme that represents the IPA symbols as single ASCII symbols. For Spanish, we used the phonetic transcriptions that are included in the lexical database B-PAL [27], [46], because these are also based on the DISC standard. For French, we used the phonetic transcriptions that are included in Lexique [28], which are based on X-Sampa. In contrast to DISC, X-Sampa is not single-coded, i.e. not every ASCII character represents an IPA symbol. The recoding of X-Sampa involved a number of substitutions of characters combinations into DISC characters. For Italian, no phonetic transcriptions were available in the lexical database used (CoLFIS [29]). In order to be able to apply a phonetic similarity measure to compatible phonetic transcriptions across all six languages, we applied a *text-to-speech* algorithm to CoLFIS’ Italian orthographic forms using a pronunciation guide [47]. This is possible because Italian has a shallow orthography. In the case of both Lexique and CoLFIS, new phonemes were identified that had to be added to the set of phonemes included in DISC. This resulted in a new coding scheme that we refer to as DISC++. Table S1 presents all the phonetic alphabets that were discussed above aligned with DISC++. Textfile S1 contains the abbreviations used in Table S1.

For our computation of phonetic similarity, we varied the Levenshtein substitution cost according to the similarity between phonemes. Phoneme similarities are assumed to play a decisive role in the match between the NLD and similarity ratings. We computed a substitution cost distribution according to the distinctive phonetic feature space as given by the IPA. The phoneme space that is represented in the IPA enabled a distance computation between phonemes. Substitution cost was calculated by measuring the Euclidean distance in the respective IPA vowel or consonant space and by adding a penalty in case at least one of the phonemes was non-pulmonic, an affricate, a diphthong, a borrowed vowel, or a long vowel. Penalties were not applied when both phonemes were long vowels or both were long affricates. Substitutions between vowels and consonants received the maximal substitution cost of 2. Our computation of phonetic similarity used phonetic transcriptions available in lexical databases. The availability of phonetic transcriptions enabled us to look at phonetic similarity without considering irregularities in grapheme to phoneme mapping. The similarity between two phonetic transcriptions was calculated by applying the NLD, as discussed in the previous section, onto phonetic transcriptions. Similar to the other measures of similarity the phonetic similarity values were validated using similarity ratings from human subjects, see the end of the results section.

## Results

The application of the orthographic and phonetic similarity measures to translation equivalents allowed the estimation of cross-language distributions of high frequency and phonetically similar cognates. The results are presented in three steps: first cross-language similarity distributions, then cognate frequency comparisons, followed by external validation of the similarity measures and the numbers of cognates automatically identified.

Various similarity patterns were observed in the cross-language cognate distributions. These patterns resulted from comparisons of cross-language orthographic similarity distributions with cross-language phonetic similarity distributions. The comparisons show how much orthographic and phonetic similarity differ according to the orthographic depth of the languages. Furthermore, the cross-language cognate distributions allowed comparisons of cognate frequency across all 15 language pairs. The comparisons show how patterns in cognate frequency relate to differences in evolutionary relatedness across languages. Finally, it is demonstrated that subjective similarity ratings and measures of evolutionary change validate the automatic measures of orthographic and phonetic similarity and the automatically identified numbers of cognates. The automatically identified cognates are available in Dataset S1. These cognates correspond to the third column of Table 2. Textfile S1 contains the abbreviations used in Dataset S1.

### Orthographic and Phonetic Similarity Patterns Across Languages

Distributions of similarity measures for 15 different language pairs revealed how translation equivalents are distributed over the range of the orthographic or phonetic similarity function. These distributions visualize how closely related and less closely related language pairs differ in specific parts of orthographic and phonetic similarity continua.

**Figure 1:**
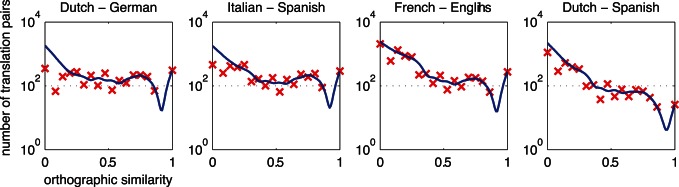
Orthographic similarity distributions across translation equivalents. The data points represent the normalized Levensthein distances binned into 18 equal parts on the obtained similarity scale. The solid line uses locally weighted scatter plot smoothing and spline interpolation over the bins. Notice the logarithmic scale on the y axis.

**Figure 2:**
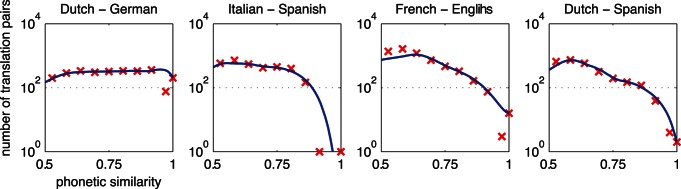
Phonetic similarity distributions across translation equivalents. See the legend of Figure 1 for a description.

Figures 1 and 2 exemplify orthographic and phonetic similarity distributions for four pivotal language pairs. For these distributions, we chose a minimum word frequency of 10. There were 23 possible orthographic similarity measures given the discreteness of the Levenshtein distance codes [24]. These measures were divided in 18 equal bins in order to distribute the orthographic similarity measures equally in the range from 0 to 1. The phonetic similarity measures were divided in 36 bins. Phonetic data were divided in twice as many bins, because substitution cost in phonetic similarity measurement could range between 0 and 2 instead of using a fixed cost of 1 as for orthographic similarity measurement. As a consequence, plots of phonetic similarity distributions start at.5 instead of 0, thus ensuring that the number of bins in the graphs stayed equal. The resulting scatter plots were smoothed using locally weighted scatter plot smoothing (LOWESS) [48]. A smoothing factor of.25 was used. This value determines the proportion of the bins that is used to smooth the curve through all of the values in the bins, using low degree polynomials. The resulting similarity degrees (represented by small crosses in Figure 1 and 2) were plotted on logarithmic y-axes. We restored numbers of form-identical cognates to their original values in order to compare identical numbers of cognates across language pairs. Therefore, a fast bump or drop in the number of form-identical cognates can be observed in each of the plotted similarity distributions. We applied *spline* interpolation to get polynomial-like shapes.

Except for Dutch-Spanish, high numbers of orthographically identical cognates are present in the four language pairs displayed. The numbers of orthographically identical cognates are similar across the first three examples, but differ between Dutch and Spanish. Orthographic similarity values are distributed evenly over the orthographic similarity continuum for the first two examples, i.e. Dutch-German and Italian-Spanish. However, this is not the case for the second two examples, i.e. French-English and Dutch-Spanish. Furthermore, the distribution of English-French lies higher in the graph than other distributions, indicating that more high frequency translation equivalents were found between these two languages. Note that the drops in almost-identical cognates reflect that only a few possible combinations of word length can result in almost-identical similarity scores. For example, a score of.75 can result from eight combinations of word length, and a score of.8 can only result from three combinations of word length. Phonetic similarity measures were distributed in a more continuous way.

Distributions of phonetic similarity reveal that similarity measures are more concentrated near the centred values, rather than being distributed equally over the phonetic similarity continuum. This is a result of the higher substitution cost that is allowed in phonetic similarity measurement. Because phonetic similarity substitution costs can be twice as much as in orthographic similarity measures, the phonetic similarity measures are distributed differently in the first halves of the examples displayed. However, the second halves, starting from a phonetic similarity of.5, distributions become comparable again.

In terms of phonetically identical translation equivalents, the distributions only show fast drops, except for Dutch-German. More orthographically than phonetically identical cognates appear to exist across language pairs. Also, the distribution of Dutch-German and Italian-Spanish seem to have a more horizontal spreading than French-English, which may indicate the relatedness between these two languages.

**Figure 3:**
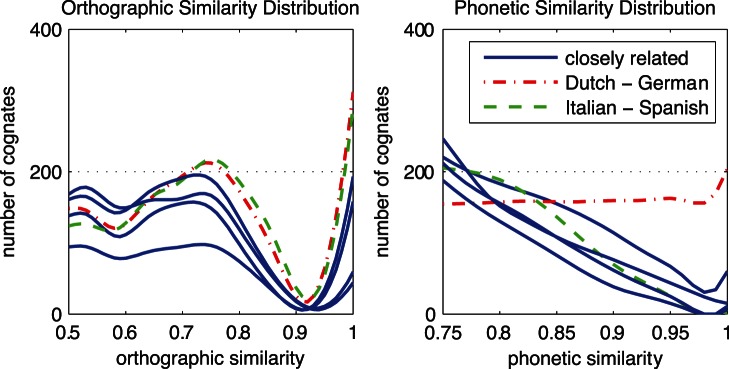
Similarity distributions of cognates for closely related language pairs. German – Dutch and Italian – Spanish are coded with dashed lines. The solid lines use locally weighted scatter plot smoothing and spline interpolation over 18 bins.

**Figure 4:**
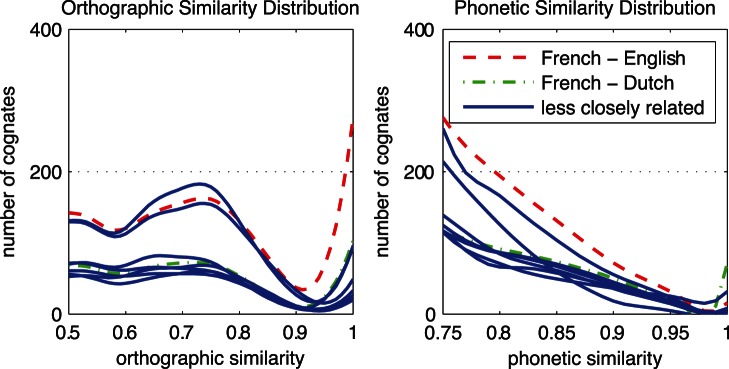
Similarity distributions of cognates for less closely related language pairs. French – English and Fench – Dutch are coded with dashed lines. See the legend of Figure 6 for a description.

By zooming in on the highly similar orthographic and phonetic cognates, the differences between the orthographic and phonetic similarity distributions become more evident. Figure 3 shows distributions of orthographic and phonetic similarity for closely related languages. Figure 4 shows remaining distributions for languages of different (sub)families. Distributions of related languages showed more differences between O and P similarity than the distributions of unrelated languages. According to Figure 3, O similarity is sometimes higher than P similarity, as in Italian-Spanish (marked by the dashed green line), and sometimes O similarity is lower than P similarity, as is the case for Dutch-German (marked by the dashed purple line). According to the right panels of both Figure 3 and Figure 4, Dutch-German is the only language pair with a high number of phonetically identical cognates. Although the differences in unrelated language pairs are smaller, some language pairs show distinctive numbers of cognates in the highest similarity ranges. Especially English-French (marked by the line with triangles) shares a large number of highly orthographically similar cognates. On the other hand, it seems that Dutch-French (marked by the line with circles) shares a large number of highly phonetically similar cognates.

In general, orthographic and phonetic similarity distributions appear to lie higher for related languages than for unrelated languages. However, especially in the higher similarity ranges (on the right hand side of the graphs), some language pairs have a higher orthographic than phonetic overlap or vice versa.

In order to determine if the orthographic and phonetic similarity measures reflect different cognate characteristics, we analyzed the overlap between both similarity measures by correlating orthographic and phonetic similarity measures. The correlations showed a clear distinction between two groups of language pairs (see Table 3). The correlations in the shallow languages were always equal or higher (*r* ranging from.32 to.55) than the correlations in the language pairs in which at least one language has a deep orthography (*r* ranging from.05 to.32), except English-Spanish (*r* = .34). Dutch-German and Italian-Spanish had the highest O-P similarity correlations, while Dutch-French and English-French had the lowest correlations.

**Table 3:** Correlations between orthographic and phonetic similarity measures of cognates.

L1–L2	Dutch	English	German	French	Spanish	Italian
Dutch (S)		.30	.42	.05	.43	.35
English (D)			.23	.19	.35	.29
German (S)				.14	.32	.47
French (D)					.32	.31
Spanish (S)						.55
Italian (S)						

S = shallow orthography, D = deep orthography. Underlined correlations involve two shallow orthographies. All correlations *p*<.0001, except French-German, *p*<.05.

The orthographic and phonetic similarity measures seem useful to quantify differences in speech and writing systems between languages. Low correlations between similarity measures indicate that both measures evaluate different characteristics of cognates. In turn, high correlations indicate similarity between writing and speech systems. High correlations of language pairs involving two shallow orthographies, demonstrate how close the mapping between speech and writing systems can be. Correlations of language pairs that involve at least one language with a deep orthography demonstrate the complex mapping between speech and writing systems across language pairs.

### Frequency Patterns of Cognates Across Languages

**Figure 5:**
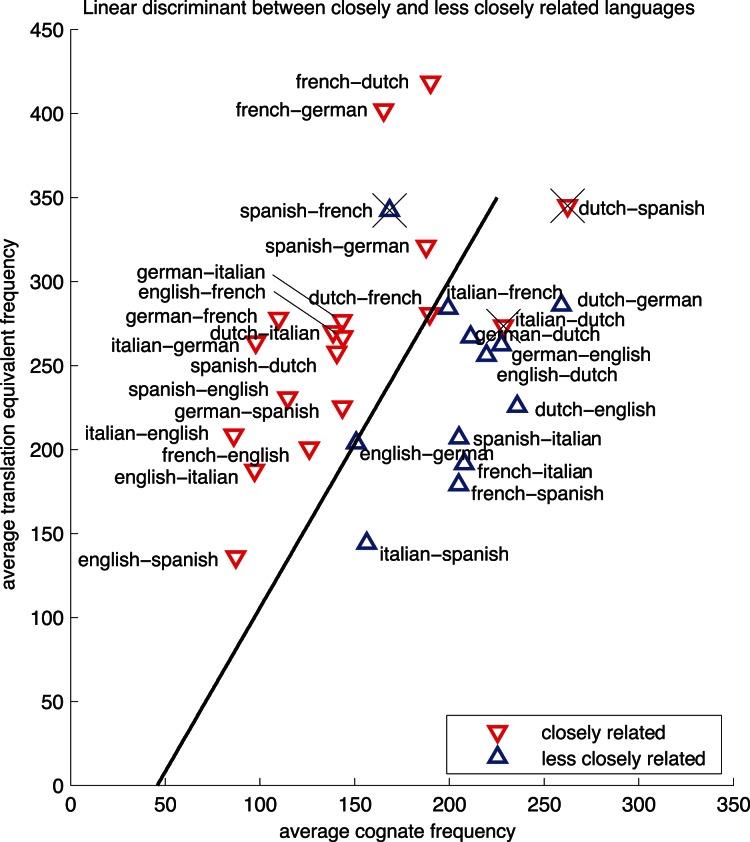
Relative cognate frequency predicts degree of genetic relatedness between languages. Average frequencies are shown for both languages in each language pair. The straight line represents the result of a linear discriminant analysis between the classes more and less closely related language pairs.



This section describes how we compared cognate frequency between more and less closely related languages pairs. To compute a measure of cognate frequency in a language pair, we estimated the frequency of the cognate’s reading as the mean of the frequencies of the L1 and L2 orthographic forms. However, because word frequency distributions differ across languages, we computed cognate frequency by the mean frequency of all L1 and L2 orthographic forms respectively (in the set of cognates that we identified by applying a phonetic similarity threshold, see Table 2). We applied the same procedure to the obtained sets of translation equivalents. This resulted in a more noisy hierarchy than we obtained for the cognate frequencies. We then investigated whether this noisy signal could be filtered out from our initial hierarchy by dividing the frequency means of cognates by the frequency means of translation equivalents. Figure 5 shows that the best distinction between more and less closely related languages depends on both average cognate frequency and average translation equivalent frequency. With a simple linear discriminant analysis, already a classification accuracy of 86.67% can be achieved, separating the class of closely related languages (within the Germanic or within the Romance subfamilies), from less closely related languages (between Germanic and Romance subfamilies). With this measure of cognate frequency, normalized by general characteristics of the obtained set of translation equivalents (see Equation 2), we obtained a hierarchy that was able to separate languages pairs with a high surface similarity (e.g. English-French) from languages pairs that are genetically closely related (e.g. English-German). The hierarchy this way is displayed in the last column of Table 2.

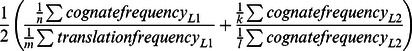



Equation 2. Relative cognate frequency can be computed by dividing the average cognate frequency in each language by the average frequency of translation equivalents.

As cognates are likely to be shorter words than translation equivalents, one might argue that we are essentially obtaining a measure of the difference in word length across more and less closely related languages. However, it is the case that more frequently used words tend to lose more characters than less frequently used words (cf. Zipf’s law [49]). For example, the common ancestor in Danish and Dutch for *car* (*automobiel),* lost -*biel* in the Dutch word, which is *auto,* and lost *automo-*, in the Danish word, which is *bil* [50]. We only included translation pairs and cognates with a word length between 3 and 8 letters, which might actually have resulted in the exclusion of more translation pairs than of cognates. So, to characterize the frequency of identified cognates, we computed the mean cognate frequencies of automatically identified cognates for each language pair and divided this by its mean translation frequency. In this way, we obtained a relative cognate frequency measure per language pair.

The hierarchy in relative cognate frequencies shows how cognate frequency is able to distinguish languages pairs from the same subfamily from language pairs from different subfamilies. In contrast, hierarchies based on numbers of cognates only were not able to distinguish English-French from language pairs from the same subfamily, which is likely to be due to borrowing as a result from language contact. For example, our estimated measure of form overlap between English and French looks relatively high given their historical relatedness. The relative frequency of these overlapping forms reveals that many cognates do not follow the same pattern of high frequency as cognates in more closely related languages do.

**Figure 6:**
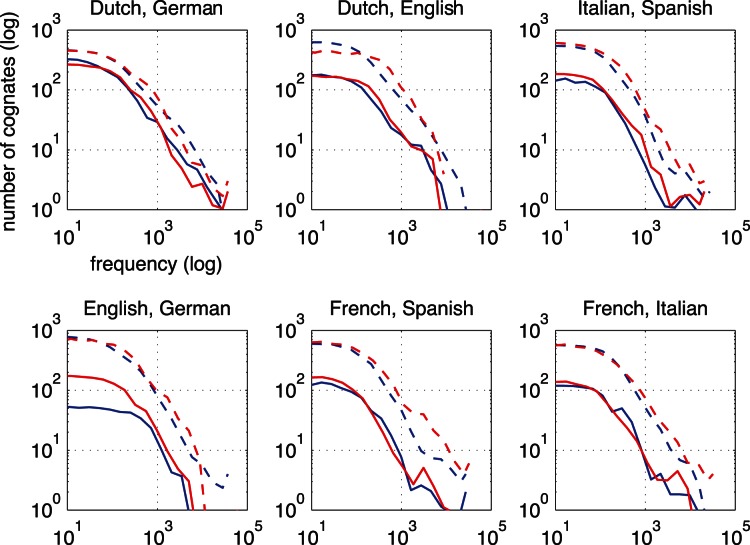
Comparisons of cognate to translation frequency distributions for six closely related language pairs. The x axes show cognate frequencies per million words. The y axes show the numbers of cognates observed. The frequency distributions of translation equivalents are plotted with dotted lines. The blue colored lines code for the L1, the red colored lines code for the L2. The order of languages in the subtitles indicate which language is the L1 and which language is the L2. The cognate frequencies are binned into 14 equal parts on the word frequency scale. The lines use locally weighted scatter plot smoothing over the bins. Notice the logartithmic scales.

**Figure 7:**
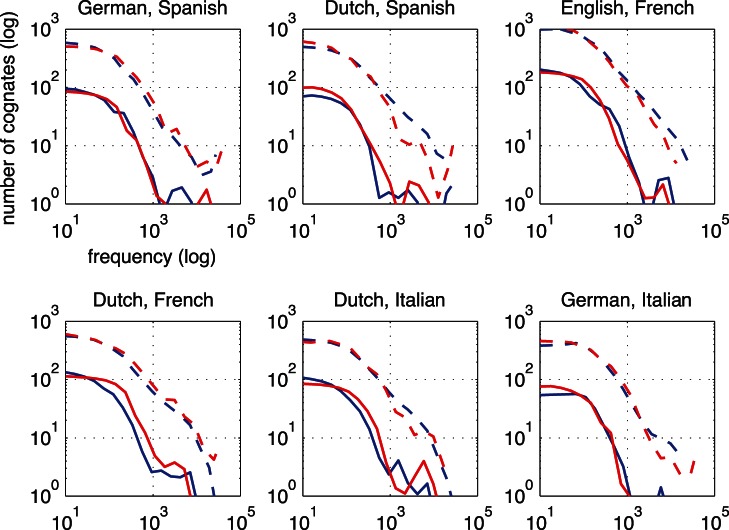
Comparisons of cognate and translation frequency distributions for six less closely related language pairs. See the legend of Figure 6 for a description.

The underlying cognate frequency distributions are plotted in Figure 6 and 7. The figures show how the frequency distributions of cognates differ slightly between the languages in each pair. The frequency distributions of translation equivalents provide a reference point for the cognate frequency distributions. As the relatedness between the language pairs decreases, also the distance to the frequency distributions of translation equivalents decreases. Generally, most cognates are found in the lower frequency bands. The cognate frequency distributions of the languages in each pair differ more in the higher frequency bands than in the lower frequency bands.

In sum, relative cognate frequency correlates highly with language relatedness, and it is possible to distinguish closely related languages from languages that are similar because of heavy borrowing.

### Validation of O and P Cognate Similarity Norms

In order to evaluate whether the list of automatically detected cognates with highly similar O and P values correspond to cognates identified by humans a validation of the computerised similarity norms was needed. This validation was conducted by applying thresholds to the scores resulting from O similarity based on the normalized Levenshtein Distance (O NLD) and P similarity based on NLD (P NLD) and then comparing both O and P NLD scores to the 1003 similarity ratings from Tokowicz et al. [25] and the 318 ratings from Dijkstra et al. [3]. Ratings from Tokowicz et al. [25] were based on both form and sound similarity judgement, whereas ratings from Dijkstra et al. [3] were available for form and sound similarity judgments separately. In both studies, rating scales ranging from 1 to 7 were used. For a number of threshold configurations, we measured correct cognate classification (percentage correct of word pairs rated higher than 5/7), and correct non-cognate classification (percentage correct of word pairs rated lower than 5/7). On the basis of this study, we used an inclusive O threshold of.5 and an inclusive P threshold of.75 for the computed similarity measures for automatic cognate identification. The results are presented in Table 4 and the similarity measures are presented in Tables S2 and S3.

**Table 4:** Classification rates as based on subjective measurements.

Data	Similarity	Threshold	Similar translations	Correctly classified	Dissimilar translations	Correctly classified
Tokowicz et al. [25]	O	.5	173	91%	714	97%
Tokowicz et al. [25]	P	.75	168	87%	676	92%
Dijkstra et al. [3]	O	.5	78	100%	184	90%
Dijkstra et al. [3]	P	.75	74	95%	174	85%

As Table 4 shows, the automatic cognate identification procedure worked very well: On average, over 90% of cognates were correctly classified. The first three translation pairs with low orthographic and phonetic ratings that were qualified as dissimilar by human subjects, but not by either automatic orthographic similarity or automatic phonetic similarity (based on ratings from Dijkstra et al. [3]) are the following: *schroef – screw* (O rating 1.88, P rating 2.25, O NLD.571, P NLD.82); *gids – guide* (O rating 3.5, P rating 2.25, O NLD.33, P NLD.77); and *koning – king* (O rating 3.88, P rating 3, O NLD.67, P NLD.80). With respect to the first word pair, participants might have misjudged the similarity due to the difference in word length. Actually, 3 of the 4 letters in the English word are present in the Dutch word. Moreover, 3 of the 4 English phonemes are also present in the Dutch phonetic transcription (*sxruf – skru*).

To evaluate the semi-continuous norms of O and P NLD, we correlated resulting scores with the rated word pairs from Tokowicz et al. [25] and from Dijkstra et al. [3]. The ratings correlated, respectively,.88 (*p*<.001) and.96 (*p*<.001) with O NLD, and.82 (*p*<.001) and.85 (*p*<.001) with P NLD. The P NLD and Raw P NLD (no substitution cost distribution applied) correlated equally with the same orthographic and phonetic similarity ratings. The P NLD norms were more continuous than the norms of the O NLD; the O NLD scores for any language pair were distributed over only 23 different values, the P NLD scores in Dutch-English were distributed over 652 different scores.

To summarize, we obtained orthographic similarity norms using a normalized Levenshtein distance measure and phonetic similarity norms with a normalized Levenshtein distance measure that made use of an IPA-based substitution cost distribution. Both measures can be applied successfully to obtain reliable measures of orthographic and phonetic similarity for given word pairs and their phonetic transcriptions. Using these measures, it is possible to automatically detect orthographically and phonetically similar translation pairs in large cross-language lexical databases or corpora. This procedure is much faster than traditional methods that require human similarity judgements.

### Validation of Automatically Identified Cognates

We hypothesized that numbers of automatically identified cognates in language pairs can predict language similarity as observed in studies that incorporate expert knowledge (e.g., [11]). More specifically, phonetic similarity and word frequency could provide a better account of language similarity than the language ordering based on orthographic distance between translation pairs alone [24]. To assess surface variation in word forms across languages, we compared automatic cognate identification with varying parameters and thresholds to the language similarity tree provided by Gray and Atkinson [11], see Introduction.

With respect to O similarity, the identified cognates from the present study correlated *r = *.60, *p*<.05) with the expert account of language relatedness [11] (*r = *.62, p<.05 based on an inclusive O threshold). The language pair with the highest number of shared cognates was Dutch-German. With respect to a minimum word frequency of the O representations, the exclusion of low frequency cognates resulted in the identification of almost half of the numbers of cognates. For a minimum word frequency of 0 opm, on average 3600 cognates were identified with a standard deviation of 1200. For a minimum word frequency of 2 opm, on average 1880 cognates were identified with a standard deviation of 854. For a minimum word frequency of 10 opm, on average 820 cognates were identified with a standard deviation of 420.

With respect to P similarity, numbers of phonetically similar cognates correlated with Gray and Atkinson [11] about as strong as correlations based on numbers of orthographically similar cognates (*r* = .59, *p*<.05 vs. *r* = .61, *p*<.05 for frequencies ≥2 opm, and *r* = .64, *p*<.05 vs. *r* = .64, *p*<.05 for frequencies ≥10 opm). The numbers of English-French, English-Italian, and English-Spanish cognates based on P similarity were always slightly lower than the numbers of cognates based on O similarity, see Table 2. This suggests that P similarity is able to distinguish cognates in these less closely related languages from those in more closely related languages. Furthermore, the P similarity ordering revealed a larger phonetic than orthographic overlap between Dutch and French. Cognate identification using phonetic similarity as a criteria revealed remarkable differences between less and more closely related languages. The language pairs that were too similar based on orthographic similarity (French-English, Spanish-English, and Italian-English) were still too similar based on phonetic similarity. However, these language pairs shared fewer phonetically similar cognates than orthographically similar cognates.

The relative cognate frequencies identified by applying a phonetic similarity threshold to automatically identified translation equivalents correlated higher with branch lengths from Gray and Atkinson [11] than numbers of phonetically similar cognates (*r = *.87, *p*<.0001 vs. *r* = .64, *p*<.05 for frequencies ≥10 opm). Relative cognate frequencies are able to distinguish between more and less closely related languages better than numbers of phonetically or orthographically similar cognates can.

## General Discussion

The present study investigated cross-language similarity in terms of phonetic and frequency characteristics between translation equivalents across six related European languages. Orthographic and phonetic transcriptions in combination with frequency measures of words were used to compare O, P, and frequency distributions of cognates. Spelling, pronunciation, and frequency data were collected by linking different lexical databases. The computation of orthographic and phonetic similarity based on the normalized Levenshtein distance (NLD) make it possible to investigate the distributions of cognates characterized by frequency and S, O, and P similarity.

The phonetic similarity of translation pairs was determined using NLD while varying substitution costs according to the distinctive phonetic feature space from the International Phonetic Alphabet. Phonetic similarity norms were more continuous than orthographic similarity norms (over 600 different phonetic similarity degrees vs. 23 orthographic similarity degrees) because we incorporated an adapted IPA coding of phonemes. The validation study that compared subjective similarity ratings with the automatically derived objective similarity measures revealed that automatically obtained similarity measures are usable as reliable replacements for subjective similarity ratings. Consequently, these automatically derived similarity measures are useful in computational models and applications in which orthographic or phonetic similarity in large sets of translation equivalents needs to be computed.

Orthographic and phonetic similarity measures reflect the orthographic depth of spelling as depending on sound systems. Orthographic and phonetic similarity distributions showed how these dependencies differ across languages. Frequency measures revealed more about the genetic relatedness of languages. Cognates had higher frequencies when they occurred between two closely related languages than when they occurred between two less closely related languages. The results can therefore be related to principles in language change across languages.

In the following two sections, we evaluate the usefulness of the observed frequency and O and P similarity characteristics of automatically identified cognates in terms of the dependency between sound and spelling systems of languages, and language change.

### Orthographic Depth in terms of Phonetic to Orthographic Mappings

Automatic phonetic similarity measures were able to detect different degrees of phonetically similar cognates. As was the case for orthographic similarity, the shape of the distributions revealed fewer translation equivalents (or more cognates) as phonetic overlap increased.

A comparison of both dimensions indicated the consistency between spelling and pronunciation of a word pair. A correlation measure across a large number of word pairs (see fourth column of Table 2) provided an overall consistency measure of spelling and pronunciation similarity. When orthographic and phonetic similarity are highly consistent (e.g., Italian-Spanish), spelling is a direct reflection of pronunciation of words for both languages. When both characteristics are different (e.g. Dutch-French), one or both languages have inconsistent grapheme-to-phoneme mappings. In our study, correlations between O and P similarity measures were able to demonstrate these distinctions. Consequently, these measures may be used to quantify compatibility between sound and spelling systems of languages.

Pronunciation of words may change over time according to communication needs. Ultimately, geographical spreading and localization of communication needs may result in the formation of different pronunciation systems. Distance between speakers may then result in proportional distances between pronunciations of words. The observed phonetic similarity distributions suggest that some pronunciation systems are indeed more compatible than others.

These patterns are similar to orthographic similarity distributions. In contrast to orthographically identical cognates [24], low numbers of phonetically identical cognates were automatically identified in the present study (see right panel of Figure 3). Dutch-German contained relatively high numbers of phonetically identical cognates whereas Italian-Spanish contained relatively high numbers of phonetically similar cognates. Italian-Spanish and Dutch-German orthographic similarity distributions were relatively similar to each other. Because the orthographic similarity distribution does not show this pattern, we conclude that the sound systems of Dutch and German produce more often cognates with higher similarity than the sound systems of Italian and Spanish. This finding is in line with the finding that the consistency of O and P similarity measures between German and Dutch is lower than the consistency of O and P similarity measures between Italian and Spanish (see Table 3). The proportion of phonetically similar to phonetically identical cognates can change across language pairs under the influence of divergence time, divergence speed, borrowing, and chance resemblance.

### Cognate Frequency and Language Change

Computationally determined orthographic and phonetic overlap in translation equivalents demonstrated how cognate distributions correspond to expert accounts of historical relatedness. The numbers of automatically identified cognates correlated strongly to branch lengths from a consensus tree of language relatedness reported by Gray and Atkinson [11]. However, the present study revealed to what extent shared language relations are visible on the surface of daily language use. In the language pairs we tested, we observed an average cognate percentage of 25% in high frequency translation equivalents (highest for Dutch-German, 60%). Thus, computational orthographic and phonetic similarity measures quantify and reveal previously invisible characteristics relevant for language relatedness estimations.

Using cognate frequency measures, we were able to separate closely related cognates from more distantly related cognates. In our study, minimum frequency thresholds enabled a closer comparison of high frequency (≥10 opm) cognates. We found that numbers of automatically identified cognates better resembled accounts of language relatedness when using minimum frequency thresholds than using no minimum frequency thresholds. Furthermore, we found that relative cognate frequency was always higher in related language pairs than in less closely related language pairs. It seems that related languages can be characterized by automatically measuring orthographic and phonetic overlap and then establishing frequency measures in automatically identified cognate sets. Here, frequency and O, and P similarity of translation equivalents were used to determine relatedness between languages through automatic identification of cognates. Such analyses showed that frequency measures of cognates indicate whether orthographically and/or phonetically similar translation equivalents belong to more or less closely related languages. The low frequency cognates in less closely related languages may indicate that these cognates have changed more and over a longer period of over time.

The relative cognate frequency in each more closely related language pair was higher than that in each less closely related language pair. The relative frequency of cognates strongly correlated with expert accounts of language relatedness (*r* = .87, *p*<.0001). It is plausible that the relative mean cognate frequency measures the recentness of shared origin. Theoretically, cognates that branch off earlier have longer time to diverge as a result of unspecified processes in language change. A longer divergence time between two words sharing a common origin may be reflected in lower average word frequencies, because many words change in their use and meaning, facilitating replacement [36]. Further circumstances may alter rates of replacement over time. Reasoning from this perspective, we conclude that change in cognate frequency is a core process in language change.

We now compare the striking patterns in relative cognate frequency (i.e., if cognates are used relatively more often than other translation pairs) across more and less related language pairs to more general correlation measures (i.e., if it is more likely that a cognate is used equally often than other translation pairs). On average, automatically identified cognates were highly similar in terms of frequency of usage in the two languages involved (average *r* = .53). Translation equivalents were also similar in terms of frequency, but less similar than cognates (average *r* = .29). An almost identical distribution of cognate frequency measures was found for Dutch-German (*r* = .97, *p*<.001). In contrast, Italian – German cognate frequencies were weakly related (*r* = .09, *p*<.001), indicating that the frequency of usage of a cognate item shared between Italian and German was practically unrelated; The Latin origin of many of the German-Italian cognates could be a reason for the observed differences in frequency of German-Italian cognates. However, less closely related languages also had high correlations, e.g., Italian-English (*r* = .92, *p*<.001). Observed differences in frequency similarity measures of cognate and translation equivalents might relate to the following problems in L1–L2 frequency alignment in translation equivalents.

Meaning overlap in translation equivalents is often multilateral, allowing overlap in a number of different ways. Frequency measures are mostly unilateral, measuring word counts as an orthographic statistic rather than taking into account its different meaning functions. For example, the translation pair *bank* – *bank* shares more than one meaning between English and Dutch. In addition, idiosyncratic meanings of *bank* exist in both English (e.g., *capsize*) and Dutch (e.g., *sofa*), mixing up the semantic alignment of L1–L2 frequency measures. On the one hand, these alignment differences relate to differences in numbers of translations of L1 words in L2. Such cross-linguistic differences in polysemy may be asymmetrical between language pairs [25]. However, asymmetric differences were not included in the semantic structure of the translation database used here. On the other hand, equivalent meanings in L2 might not always exist (for example English-Chinese), resulting in more ambiguous semantic mappings between translation equivalents. Semantic matching of L1–L2 frequency similarity measures might expose more cross-linguistic differences than frequency similarity only.

### Conclusion

To our knowledge, this is the first study in which dependencies between sound and spelling systems and principles in language change are discussed in terms of distributions of automatically identified cognates. In addition to Marian et al. [34], which is a useful resource for neighbourhood densities, our study now provides a resource for cognate distributions. The added semantic component makes it possible to compare languages in pairs instead of in isolation. The cognates that are automatically identified this way are crucial for an understanding of cross-language processing. The newly estimated cognate distributions by frequency and phonological overlap confirm the importance of these two dimensions for understanding cross-language similarity. Automatically identified distributions of cognates appear to differ in the same way as commonly used categorizations in terms of orthographic depth and language change.

The observed patterns in O and P similarity have consequences for our understanding of orthographic depth. Our observations show that the regularity between O and P is indeed associated with typical patterns in cognate distributions. Underlying may be the dependence of writing system properties on spoken language characteristics. Also, cognates are more useful for quantifying orthographic depth than translation equivalents, because the O-P regularity pattern is more faithfully represented in cognates.

Furthermore, the observed patterns in frequency distributions have consequences for our hypotheses concerning language change. The degree of relatedness between languages seems to be strongly associated with the frequency of cognates, even more strongly than with O and P overlap. This illustrating the central role that word frequency must take in cross-linguistic study. This finding provides empirical support using semi-complete lexicons for the explanation given by Pagel et al. [36] that more frequent words are more resistant against lexical replacement. The frequency of cognates appears to be proportional to the divergence time between two languages. For example, Dutch and German have more recently branched off from each other than Dutch and Spanish, and as a consequence the relative frequency of Dutch-German cognates is higher than of Dutch-Spanish cognates.

In all, the present study has successfully applied automatic identification algorithms to characterize distributions of cognates in terms of frequency and phonetic similarity. Automatic cognate identification is useful for psycholinguists and linguists to quantify cross-linguistic differences in word processing, and spelling and sound systems in general. The newly developed similarity measures can be applied in computational models and tools to quickly provide reliable orthographic or phonetic similarity measures. We have identified large lists of cognates across six languages that are in demand for experimental studies on bilingual word recognition and word production. In sum, linguistic data from lexical and translational databases can be used in innovative computational ways to quantify similarities and differences across languages.

## Supporting Information

Table S1 **Keys to the phonetic transcriptions used in Dataset S1.** Codes from several phonetic alphabets (i.e. IPA, DISC, SAMPA, CELEX, CPA, X-SAMPA, Lexique, and CoLFIS) are aligned with that of DISC++. Textfile S1 contains the abbreviations used in Table S1.
(DOCX)

Table S2 **Subjective form and meaning similarity ratings for 1004 cognates and non-cognates.** Automatic form and meaning similarity measures are added for comparison.
(DOCX)

Table S3 **Subjective form similarity ratings (both orthographic and phonetic similarity ratings) for 319 cognates and non-cognates.** Automatic form and meaning similarity measures are added for comparison.
(DOCX)

Dataset S1 **A collection of 15 separate plain text files that contain the automatically identified cognates corresponding to the third column of** **Table 2** **.** Measures of orthographic and phonetic similarity as well as frequency information are included. Textfile S1 contains the abbreviations used in the headers of the text files.
(ZIP)

Textfile S1 **Meanings of the abbreviations used in Dataset S1 and Table S1.**
(TXT)
