# Supplementary material for: Cross-Language Distributions of High Frequency and Phonetically Similar Cognates
Source: PLoS One. 2013 May 10;8(5):e63006. doi: 10.1371/journal.pone.0063006 (PMC3651159; doi:10.1371/journal.pone.0063006)
Supplement: Table S1 — Keys to the phonetic transcriptions used in Dataset S1. Codes from several phonetic alphabets (i.e. IPA, DISC, SAMPA, CELEX, CPA, X-SAMPA, Lexique, and CoLFIS) are aligned with that of DISC++. Textfile S1 contains the abbreviations used in Table S1. (DOCX) [file pone.0063006.s001.docx]

Table S1. Phonetic alphabets

| Cat | IPA | example | disc | disc | sam | ce- | cpa | x-sa- | Lex- | CoL- |
| --- | --- | --- | --- | --- | --- | --- | --- | --- | --- | --- |
|  |  |  | ++ |  | pa | lex |  | mpa | Ique | FIS |
| PLM CNS |  |  |  |  |  |  |  |  |  |  |
| PLSV | p | put | p | p | p | p | p | p | p | p |
|  | b | bad | b | b | b | b | b | b | b | b |
|  | t | tak | t | t | t | t | t | t | t | t |
|  | d | dak | d | d | d | d | d | d | d | d |
|  | k | kat | k | k | k | k | k | k | k | c, ch |
|  | ɡ | goal | g | g | g | g | g | g | g | gh, g |
| Nasal | m | mat | m | m | m | m | m | m | m | m |
| *Syll. CNS* |  | idealisM | F | F | M | m | m | M= |  |  |
|  | n | nat | n | n | n | n | n | n | n | n |
| *Syll. CNS* |  | burdeN | H | H | n | n | n | H= | n |  |
|  | ɲ | gnomo | µ |  |  |  |  | J | N | gn |
|  | ŋ | lang | N | N | N | N | N | N | G |  |
| *Syll. CNS* | n | bacoN | C | C | N | N | N | N= | N |  |
| *Syll. CNS* |  | fatheR | R | R | r* | r* | r* | r*= | r |  |
|  | ʀ | rat | r | r | r | r | r | R\ | R | r |
| FRCAT | f | fiets | f | f | f | f | f | f | f | f |
|  | v | vat | v | v | v | v | v | v | v | v |
|  | θ |  | T | T |  |  |  | T | T |  |
|  | ð |  | D |  |  |  |  | D | D |  |
|  | s | sap | s | s | s | s | s | s | s | s |
|  | z | zat | z | z | z | z | z | z | z | s-m, s-b, |
|  |  |  |  |  |  |  |  |  |  | s-o, s-u |
|  | ∫ | sjaal | S | S | S | S | S | S | S | sc-e, sci |
|  | ʒ | ravage | Z | Z | Z | Z | Z | Z | Z |  |
|  | x | licht,gaat | x | x | x | x | x | x | x | x |
|  | ɣ | regen | G | G | G | G | G | G | G |  |
|  | h | had | h | h | h | h | h | h | h | h |
| APPRX | ʋ | wat | w | w | w | w | w | P, v\ |  | w |
|  | j | jas | j | j | j | j | j | j | J | i |
| Lat.  APPRX | l | lat | l | l | l | l | l | l | l | l |
| *Syll. CNS* |  | dangLe | P | P | l | l | l | l= |  |  |
|  | ʎ |  | L | L |  |  |  | L |  | gli |
| NPULM |  |  |  |  |  |  |  |  |  |  |
| CO-ARTIC | ɥ |  | ¬ |  |  |  |  | H | 8 |  |
|  | wa |  | ­ |  |  |  |  | wa |  | ua |
|  | we |  | ® |  |  |  |  | we |  | ue |
|  | wi |  | ¯ |  |  |  |  | wi |  | ui |
|  | wo |  | ° |  |  |  |  | wO |  | uo |
| AFFRC |  | PFerd | + | + | pf | pf | pf |  |  |  |
|  |  | Zahl | = | = | ts | ts | C/ |  |  |  |
|  | ts | Matsch | J | J | tS | tS | T/ |  |  | z |
|  | tts |  | ¡ |  |  |  |  |  |  | i-zz, a-zz |
|  | dz | jazz | _ | _ | dZ | dZ | J/ | dz | dz | z (at pos 0) |
|  | ddz |  | ¢ |  |  |  |  |  |  | e-zz, o-zz |
|  | t∫ |  | £ |  |  |  |  |  |  | ci, ce |
|  | kw |  | ¤ |  |  |  |  |  |  | qu |
|  | dʒ |  | ¥ |  |  |  |  |  |  | gi, g-e |
|  | ddʒ |  | ¦ |  |  |  |  |  |  | gg-i, gg-e |
|  | ʎʎ |  | § |  |  |  |  |  |  | a-gli, u-gli,  i-gli |
|  | ɲɲ |  | ¨ |  |  |  |  |  |  | a-gno,  i-gno |
|  | ss |  | © |  |  |  |  |  |  | a-sc,  u-sci |
|  | ∫∫ |  | ª |  |  |  |  |  |  | a-sci |
|  | tt∫ |  | « |  |  |  |  |  |  | cci, cc |
| VWL |  |  |  |  |  |  |  |  |  |  |
| Long | i: | liep | i | i | i: | i: | i: | i | i | i,ì |
|  | y: | buut | y | y | y: | y: | y: | y | y | y |
|  | e: | leeg | e | e | e: | e: | e: | e | 5 | e,é |
|  | ø: | deuk | \| | \| | \|: | &: | q: | 2 | 2 |  |
|  | a: | laat | a | a | a: | a: | a: | a | @ | a,à |
|  | o: | boom | o | o | o: | o: | o: | o | § | o |
|  | u: | boek | u | u | u: | u: | u: | u | u | u, ù |
|  | ɑ: | advAntage | # | # | A: | A: | A: | A: |  |  |
|  | ɔ: | Allround | $ | $ | O: | O: | O: |  | O |  |
|  | ɜ: | teamwOrk | 3 | 3 | 3: | 3: | @: | 3 |  |  |
|  | ɛ: | kAEse | ) | ) | E: | E: | E: |  |  |  |
| Short | ɪ | lip | I | I | I | I | I | I |  |  |
|  | ʏ | pfUEtze | Y | Y | Y | Y | Y | Y |  |  |
|  | ɛ | leg | E | E | E | E | E | E | E | e, è |
|  | œ | gOEtter | / | / | / | Q | Q |  |  |  |
|  | ɑ | lat | A | A | A | A | A | A | a |  |
|  | æ | trap (EN) | { | { | { | & | ^/ | { |  |  |
|  | a | hAt | & | & | a | a | a |  |  |  |
|  | ɒ | pot | Q | Q | Q | O | O |  |  |  |
|  | ɔ | bom | O | O | O | O | O | O | O | o, ò |
|  | ʉ | put | } | } | } | U | Y/ | } |  |  |
|  | ʌ | plum (EN) | V | V | V | V | ^ | V |  |  |
|  | ʊ | foot (EN) | U | U | U | U | U | U |  |  |
|  | ə | gelijk | @ | @ | @ | @ | @ | @ | ° |  |
| BRWD | i:: | analyse | ! | ! | i:: | i:: | i:: |  |  |  |
|  | y:: | centrifuge | ( | ( | y:: | y:: | y:: |  |  |  |
|  | ɛ: | scene | ) | ) | E: | E: | E: |  |  |  |
|  | œ: | ferule | * | * | /: | U: | Q: | 9 | 9 |  |
|  | ɒ: | zone | < | < | Q: | O: | o: | Q |  |  |
|  | æ~ | IMpromptu | c | c | {~ | &~ | ^/~ |  |  |  |
|  | ɑ~: | detENte | q | q | A~: | A~: | A~: |  |  |  |
|  | æ~: | bassIN | 0 | 0 | {~: | {~: | ^/~: |  |  |  |
|  | ɔ~: | affrONt | ~ | ~ | O~: | O:~ | O:~ |  |  |  |
|  | ɨ | parfUM | ^ | ^ | /~: | Q~: | Q~: | 1 | 1 |  |
| DPHTNG | ɛi | wijs | K | K | EI | EI | y/ |  |  |  |
|  | œy | huis | L | L | /I | UI | q/ |  |  |  |
|  | ɑu | koud | M | M | Au | AU | A/ |  |  |  |
|  | eI | nAtive | 1 | 1 | eI | eI | e/ | 1 |  |  |
|  | aI | shYlock | 2 | 2 | aI | aI | a/ | 2 |  |  |
|  | ɔI | playbOY | 4 | 4 | OI | OI | o/ | 4 |  |  |
|  | ɘʊ | -- | 5 | 5 | @U | @U | O/ | 5 | 5 |  |
|  | aʊ | allrOUnd | 6 | 6 | aU | aU | A/ | 6 |  |  |
|  | Iɘ | -- | 7 | 7 | I@ | I@ | I/ | 7 |  |  |
|  | ɛɘ | -- | 8 | 8 | [E@](mailto:E@) | E@ | E/ | 8 | 8 |  |
|  | ʊɘ | -- | 9 | 9 | [U@](mailto:U@) | U@ | U/ | 9 | 9 |  |
|  | ai | wEIt | W | W | ai | ai | a/ |  |  |  |
|  | au | hAUt | B | B | au | au | A/ |  |  |  |
|  | ɔy | frEUt | X | X | Oy | Oy | o/ |  |  |  |
