# Supplementary material for: Cross-Language Distributions of High Frequency and Phonetically Similar Cognates
Source: PLoS One. 2013 May 10;8(5):e63006. doi: 10.1371/journal.pone.0063006 (PMC3651159; doi:10.1371/journal.pone.0063006)
Supplement: Table S2 — Subjective form and meaning similarity ratings for 1004 cognates and non-cognates. Automatic form and meaning similarity measures are added for comparison. (DOCX) [file pone.0063006.s002.docx]

Table S2. Stimulus materials from [1]

This list contains form similarity ratings (form rating) and semantic similarity ratings (S rating) for 1004 cognates and non-cognates provided by [1]. We have added similarity scores based on automatic orthographic (O sim) and phonetic (P sim) similarity measures. Phonetic transcriptions (P1/P2) were used for the computation of phonetic similarity.

1. Tokowicz N, Kroll JF, De Groot AMB, Van Hell JG (2002) Number-of-translation norms for Dutch—English translation pairs: A new tool for examining language production. Behavior Research Methods 34: 435–451.

Table 23. Stimulus materials from [1] with S, O, and P similarity measures.

| Dutch | English | P1 | P2 | O sim | P sim | form rating | S rating |
| --- | --- | --- | --- | --- | --- | --- | --- |
| aandacht | attention | andAxt | @tEnSH | 0.111 | 0.625 | 2.62 | 6.88 |
| aankomst | arrival | aNkOmst | @r2vP | 0.125 | 0.629 | 2.38 | 7 |
| aanmelding | entry | anmEldIN | EntrI | 0.1 | 0.781 | 1.38 | 6 |
| aanzoek | proposal | anzuk | pr@p5zP | 0.125 | 0.679 | 1.25 | 6.5 |
| aap | ape | ap | 1p | 0.333 | 0.75 | 6.12 | 5.88 |
| aap | monkey | ap | mVNkI | 0 | 0.7 | 1.12 | 7 |
| aard | nature | art | n1J@R | 0.333 | 0.74 | 1.12 | 6.38 |
| aardappel | potato | ardAp@l | p@t1t5 | 0.111 | 0.679 | 1 | 6.88 |
| aardbei | strawberry | ardbK | str$b@rI | 0.3 | 0.744 | 1 | 7 |
| aarde | soil | ard@ | s4l | 0 | 0.587 | 5.12 | 7 |
| aarde | earth | ard@ | 3T | 0.4 | 0.675 | 1.25 | 5.88 |
| aardig | nice | ard@x | n2s | 0 | 0.68 | 1.12 | 6.62 |
| achterbak | trunk | Axt@rbAk | trVNk | 0.333 | 0.781 | 1.25 | 6.75 |
| actie | action | Aksi | {kSH | 0.667 | 0.688 | 5.75 | 6.88 |
| adelaar | eagle | ad@lar | igP | 0.143 | 0.7 | 2 | 6.29 |
| afkeer | dislike | Afker | dIsl2k | 0 | 0.625 | 1.25 | 6.12 |
| afstand | distance | AfstAnt | dIst@ns | 0.5 | 0.715 | 1.25 | 7 |
| afval | waste | AfAl | w1st | 0 | 0.5 | 1.25 | 5.88 |
| afval | trash | AfAl | tr{S | 0 | 0.5 | 1.43 | 6.29 |
| afval | rubbish | AfAl | rVbIS | 0 | 0.55 | 1.12 | 6 |
| appel | apple | Ap@l | {pP | 0.6 | 0.8 | 6.62 | 7 |
| arend | eagle | ar@nt | igP | 0 | 0.6 | 2.75 | 7 |
| attentie | attention | AtEnsi | @tEnSH | 0.778 | 0.792 | 6 | 6.75 |
| auteur | writer | Mt\|r | r2t@R | 0.333 | 0.76 | 6.12 | 7 |
| auteur | author | Mt\|r | $T@R | 0.667 | 0.637 | 1.38 | 5.88 |
| auto | car | Mto | k#R | 0 | 0.5 | 1.5 | 6.75 |
| bad | bath | bAt | b#T | 0.5 | 0.817 | 5.5 | 6.88 |
| bakker | bakery | bAk@r | b1k@rI | 0.667 | 0.875 | 5.62 | 7 |
| bakker | baker | bAk@r | b1k@R | 0.833 | 0.89 | 5.12 | 5.88 |
| bal | dance | bAl | d#ns | 0.2 | 0.675 | 6.38 | 7 |
| bal | ball | bAl | b$l | 0.75 | 0.834 | 1.29 | 5.57 |
| bandiet | crook | bAndit | krUk | 0 | 0.625 | 1.12 | 6.62 |
| bank | couch | bANk | k6J | 0 | 0.688 | 6.88 | 5.88 |
| bank | bench | bANk | bEnJ | 0.4 | 0.625 | 4 | 5.62 |
| bank | bank | bANk | b{Nk | 1 | 0.875 | 1.25 | 6.75 |
| bankje | bench | bANkj@ | bEnJ | 0.333 | 0.691 | 4.25 | 6.29 |
| basis | basic | baz@s | b1sIk | 0.8 | 0.72 | 4.62 | 6.38 |
| basis | basis | baz@s | b1sIs | 1 | 0.8 | 4.88 | 5.75 |
| basis | base | baz@s | b1s | 0.6 | 0.82 | 7 | 6.75 |
| bedoeling | meaning | b@dulIN | minIN | 0.444 | 0.786 | 1.38 | 6.25 |
| bedreiging | threat | b@drKGIN | TrEt | 0.2 | 0.744 | 1 | 6.62 |
| bedrieger | crook | b@driG@r | krUk | 0.111 | 0.688 | 1.12 | 5.75 |
| bedrieging | betrayal | NA | bItr1@l | 0.3 | NA | 2.38 | 6 |
| bedrog | betrayal | b@drOx | bItr1@l | 0.375 | 0.771 | 3.38 | 5.88 |
| beeld | insight | belt | Ins2t | 0 | 0.69 | 1.12 | 5.38 |
| been | leg | ben | lEg | 0.25 | 0.5 | 2 | 6.88 |
| begrafenis | funeral | b@Graf@nIs | fjun@r@l | 0.1 | 0.7 | 1.12 | 6.88 |
| bekentenis | confession | b@kEnt@nIs | k@nfESH | 0.1 | 0.75 | 1.12 | 7 |
| belangstelling | interest | b@lANstElIN | Intr@st | 0.214 | 0.728 | 1.25 | 6.5 |
| belofte | promise | b@lOft@ | prQmIs | 0.143 | 0.607 | 1.25 | 6.75 |
| benodigdheid | necessity | NA | nIsEs@tI | 0.083 | NA | 1.75 | 6 |
| benzine | gas | bEnzin@ | g{s | 0 | 0.75 | 1 | 6 |
| berg | mountain | bErx | m6ntIn | 0 | 0.667 | 1.38 | 7 |
| bericht | message | b@rIxt | mEsI_ | 0.143 | 0.766 | 1 | 6.88 |
| beroemd | fame | b@rumt | f1m | 0.143 | 0.725 | 1.12 | 5.62 |
| bescherming | protection | b@sxErmIN | pr@tEkSH | 0.182 | 0.717 | 1.25 | 6.88 |
| beschrijving | description | b@sxrKvIN | dIskrIpSH | 0.5 | 0.667 | 1.75 | 6.88 |
| beslissing | decision | b@slIsIN | dIsIZH | 0.4 | 0.75 | 1.38 | 6.88 |
| besluit | decision | b@slLt | dIsIZH | 0.25 | 0.65 | 1.43 | 6.86 |
| bestelling | order | b@stElIN | $d@R | 0.1 | 0.718 | 1.38 | 6.5 |
| betekenis | meaning | b@tek@nIs | minIN | 0.222 | 0.75 | 1.12 | 7 |
| bevel | order | b@vEl | $d@R | 0.2 | 0.65 | 1.12 | 6.62 |
| bevel | command | b@vEl | k@m#nd | 0 | 0.625 | 1.75 | 6.5 |
| bewijs | prove | b@wKs | pruv | 0 | 0.65 | 1.38 | 6.38 |
| bewijs | proof | b@wKs | pruf | 0 | 0.65 | 1.29 | 6.86 |
| bewijs | evidence | b@wKs | EvId@ns | 0.125 | 0.715 | 1.25 | 6.38 |
| bezit | property | b@zIt | prQp@tI | 0.125 | 0.786 | 1.25 | 6.62 |
| bezit | possession | b@zIt | p@zESH | 0.2 | 0.825 | 1 | 6.25 |
| biecht | confession | bixt | k@nfESH | 0.1 | 0.643 | 1.25 | 6.75 |
| bier | ale | bir | 1l | 0.25 | 0.634 | 1.25 | 5.62 |
| bier | beer | bir | b7R | 0.75 | 0.867 | 6.12 | 6.88 |
| bij | with | bK | wID | 0.25 | 0.65 | 1.25 | 5.88 |
| bij | bee | bK | bi | 0.333 | 0.95 | 4.38 | 7 |
| bij | at | bK | NA | 0 | NA | 1.12 | 4.88 |
| bijbel | bible | bKb@l | b2bP | 0.5 | 0.88 | 4.88 | 7 |
| bijeenkomst | meeting | bKeNkOmst | mitIN | 0.182 | 0.761 | 1.38 | 6.75 |
| blaam | blame | blam | bl1m | 0.6 | 0.875 | 5.75 | 6.5 |
| blad | sheet | blAt | Sit | 0 | 0.738 | 1.14 | 6.25 |
| bleek | fair | blek | f8R | 0 | 0.6 | 1.38 | 3 |
| bliksem | lightning | blIks@m | l2tnIN | 0.111 | 0.707 | 1.88 | 6.75 |
| blind | blind | blInt | bl2nd | 1 | 0.99 | 6.75 | 6.88 |
| bloem | flower | blum | fl6@R | 0.5 | 0.68 | 1.62 | 6.88 |
| blok | square | blOk | skw8R | 0 | 0.65 | 6.12 | 6.62 |
| blok | block | blOk | blQk | 0.8 | 0.875 | 1.12 | 4.12 |
| blokkade | block | blOkad@ | blQk | 0.5 | 0.822 | 4.12 | 4.75 |
| bod | bid | bOt | bId | 0.667 | 0.834 | 5.62 | 6.62 |
| bod | offer | bOt | Qf@R | 0 | 0.599 | 1 | 6 |
| boek | book | buk | bUk | 0.75 | 0.9 | 6.25 | 7 |
| boer | farmer | bur | f#m@R | 0.333 | 0.74 | 1.12 | 6.88 |
| boerderij | farm | burd@rK | f#m | 0.111 | 0.679 | 1.12 | 7 |
| boezem | breast | buz@m | brEst | 0.333 | 0.7 | 5.43 | 6.71 |
| boezem | bosom | buz@m | bUz@m | 0.5 | 0.94 | 2.5 | 5.75 |
| bont | fur | bOnt | f3R | 0 | 0.663 | 1.25 | 6.62 |
| boodschap | message | botsxAp | mEsI_ | 0.111 | 0.679 | 1.38 | 6 |
| boodschap | errand | botsxAp | Er@nd | 0 | 0.657 | 1.38 | 6.88 |
| boom | tree | bom | tri | 0 | 0.567 | 1.29 | 6.86 |
| boomstronk | trunk | bomstrONk | trVNk | 0.4 | 0.889 | 2.38 | 5.25 |
| boosheid | anger | boshKt | {Ng@R | 0.125 | 0.575 | 1.25 | 6.38 |
| boot | ship | bot | SIp | 0 | 0.667 | 6.12 | 6.88 |
| boot | boat | bot | b5t | 0.75 | 0.884 | 1.12 | 6.5 |
| borst | chest | bOrst | JEst | 0.4 | 0.75 | 5 | 6.38 |
| borst | breast | bOrst | brEst | 0.5 | 0.9 | 2.38 | 6.43 |
| borstkas | chest | bOrstkAs | JEst | 0.25 | 0.75 | 2.12 | 6.5 |
| bos | forest | bOs | fQrIst | 0.333 | 0.709 | 1.5 | 6.75 |
| bos | wood | bOs | wUd | 0.25 | 0.5 | 1.25 | 5.62 |
| bot | rude | bOt | rud | 0 | 0.667 | 2.12 | 5.5 |
| bot | bone | bOt | b5n | 0.5 | 0.75 | 3.12 | 6.88 |
| bot | blunt | bOt | blVnt | 0.4 | 0.9 | 1 | 6.38 |
| broek | trousers | bruk | tr6z@z | 0.375 | 0.692 | 1.12 | 6.75 |
| broek | pants | bruk | p{nts | 0 | 0.7 | 1.12 | 6.5 |
| broer | brother | brur | brVD@R | 0.714 | 0.825 | 3.71 | 7 |
| brood | bread | brot | brEd | 0.6 | 0.875 | 5.62 | 6.62 |
| bruid | bride | brLt | br2d | 0.6 | 0.925 | 5.38 | 7 |
| bureau | desk | byro | dEsk | 0.167 | 0.5 | 1.12 | 6.38 |
| cadeau | gift | kado | gIft | 0 | 0.75 | 1.25 | 6.88 |
| cape | cloak | kep | kl5k | 0.2 | 0.725 | 7 | 3.5 |
| cape | cape | kep | k1p | 1 | 0.967 | 2.75 | 5.38 |
| cirkel | cycle | sIrk@l | s2kP | 0.333 | 0.9 | 6.38 | 6.88 |
| cirkel | circle | sIrk@l | s3kP | 0.5 | 0.825 | 3.25 | 2.88 |
| citroen | lemon | sitrun | lEm@n | 0.286 | 0.625 | 1.43 | 6.43 |
| creatie | creation | krejatsi | kri1SH | 0.75 | 0.769 | 5.88 | 6.38 |
| crimineel | crook | kriminel | krUk | 0.222 | 0.75 | 2 | 6.62 |
| cru | crude | kry | krud | 0.6 | 0.813 | 5.75 | 6.25 |
| cultuur | culture | k}ltyr | kVlJ@R | 0.714 | 0.817 | 5.75 | 7 |
| cyclus | circle | sikl}s | s3kP | 0.333 | 0.825 | 3.38 | 3.25 |
| cyclus | cycle | sikl}s | s2kP | 0.667 | 0.85 | 5.38 | 6.12 |
| daad | deed | dat | did | 0.5 | 0.834 | 5.5 | 6.12 |
| dag | day | dAx | d1 | 0.667 | 0.75 | 6.12 | 7 |
| dageraad | dawn | daG@rat | d$n | 0.25 | 0.764 | 2.5 | 6.75 |
| dak | roof | dAk | ruf | 0 | 0.5 | 1.12 | 6.88 |
| daling | descent | dalIN | dIsEnt | 0.286 | 0.709 | 2.62 | 6.12 |
| daling | fall | dalIN | f$l | 0.333 | 0.7 | 2.5 | 6.38 |
| daling | decline | dalIN | dIkl2n | 0.571 | 0.784 | 1.12 | 4.38 |
| dame | dame | dam@ | d1m | 1 | 0.813 | 7 | 5.71 |
| dame | lady | dam@ | l1dI | 0.25 | 0.688 | 1.25 | 6.62 |
| dans | dance | dAns | d#ns | 0.6 | 0.988 | 6.25 | 6.62 |
| dansen | dance | dAns@ | d#ns | 0.667 | 0.94 | 4.75 | 7 |
| dapper | bold | dAp@r | b5ld | 0 | 0.69 | 1.12 | 6.12 |
| dapper | brave | dAp@r | br1v | 0.167 | 0.75 | 1.38 | 6.62 |
| darm | bowel | dArm | b6@l | 0 | 0.563 | 1.14 | 4.29 |
| datum | date | dat}m | d1t | 0.6 | 0.8 | 5 | 7 |
| deel | part | del | p#t | 0 | 0.667 | 1.62 | 6.25 |
| deken | blanket | dek@ | bl{NkIt | 0.286 | 0.679 | 1.43 | 6 |
| den | pine | dEn | p2n | 0.25 | 0.667 | 1.12 | 6.88 |
| deugd | virtue | d\|xt | v3tju | 0 | 0.65 | 1.5 | 6.88 |
| dief | crook | dif | krUk | 0 | 0.563 | 1.62 | 5.88 |
| dief | thief | dif | Tif | 0.6 | 0.834 | 5.25 | 6.88 |
| dienstmeisje | maid | dinstmKsj@ | m1d | 0.167 | 0.745 | 1.88 | 6.75 |
| dierenarts | vet | dir@Arts | vEt | 0.2 | 0.737 | 1.12 | 7 |
| dierenarts | veterinarian | dir@Arts | vEt@rIn8r7n | 0.417 | 0.746 | 1.25 | 6.88 |
| dij | thigh | dK | T2 | 0.2 | 0.6 | 5.29 | 7 |
| ding | thing | dIN | TIN | 0.6 | 0.834 | 6.17 | 6.86 |
| directie | direction | dirEksi | dIrEkSH | 0.778 | 0.85 | 5.86 | 3.29 |
| dochter | daughter | dOxt@r | d$t@R | 0.625 | 0.942 | 4.12 | 6.88 |
| dokter | doctor | dOkt@r | dQkt@R | 0.667 | 0.909 | 6.25 | 6.88 |
| domein | property | domKn | prQp@tI | 0.25 | 0.679 | 6 | 7 |
| domein | domain | domKn | d5m1n | 0.833 | 0.87 | 1.38 | 4.12 |
| doos | box | dos | bQks | 0.25 | 0.688 | 1.75 | 7 |
| dorp | village | dOrp | vIlI_ | 0 | 0.64 | 1.12 | 6.88 |
| dorpje | village | NA | vIlI_ | 0.143 | NA | 1.38 | 7 |
| draad | wire | drat | w2@R | 0 | 0.613 | 3.62 | 6 |
| draad | thread | drat | TrEd | 0.5 | 0.75 | 1.25 | 6.5 |
| draadje | thread | dratj@ | TrEd | 0.286 | 0.75 | 2.38 | 6.25 |
| dreigement | threat | drKG@mEnt | TrEt | 0.2 | 0.806 | 1.62 | 6.62 |
| dreiging | threat | drKGIN | TrEt | 0.125 | 0.742 | 2.5 | 6.5 |
| driehoek | triangle | drihuk | tr2{NgP | 0.25 | 0.772 | 1.62 | 7 |
| druif | grape | drLf | gr1p | 0.2 | 0.675 | 1.62 | 6.12 |
| drukte | crowd | dr}kt@ | kr6d | 0.167 | 0.766 | 1.62 | 4.75 |
| duif | pigeon | dLf | pI_In | 0 | 0.73 | 4.38 | 6.88 |
| duif | dove | dLf | dVv | 0.25 | 0.834 | 1.12 | 6.75 |
| duim | thumb | dLm | TVm | 0.2 | 0.667 | 3 | 7 |
| dun | slim | d}n | slIm | 0 | 0.599 | 1.57 | 5.86 |
| dun | thin | d}n | TIn | 0.25 | 0.716 | 2.88 | 6.88 |
| dwang | compulsion | dwAN | k@mpVlSH | 0 | 0.65 | 1.38 | 6.38 |
| dwang | pressure | dwAN | prES@R | 0 | 0.584 | 1.12 | 6.12 |
| ede | oath | NA | 5T | 0 | NA | 2.75 | 6.75 |
| edel | noble | ed@l | n5bP | 0.2 | 0.725 | 1.62 | 6.75 |
| educatie | education | edykatsi | E_Uk1SH | 0.778 | 0.682 | 6.12 | 6.5 |
| eed | oath | et | 5T | 0 | 0.575 | 3.5 | 6.62 |
| eend | duck | ent | dVk | 0 | 0.667 | 1.5 | 7 |
| eenheid | measure | enhKt | mEZ@R | 0.143 | 0.59 | 1.5 | 5.25 |
| eenheid | unity | enhKt | jun@tI | 0.143 | 0.725 | 2.75 | 6.38 |
| eenvoud | single | envMt | sINgP | 0.143 | 0.54 | 1.12 | 6.88 |
| eenvoud | simplicity | envMt | sImplIs@tI | 0 | 0.695 | 1.25 | 3.25 |
| eenvoudig | simplicity | envMd@x | sImplIs@tI | 0.1 | 0.645 | 1 | 6.25 |
| eenvoudigheid | simplicity | envMd@xhKt | sImplIs@tI | 0.077 | 0.62 | 1.25 | 6.5 |
| eerbied | honor | erbit | NA | 0 | NA | 1.25 | 6.38 |
| eerbied | respect | erbit | rIspEkt | 0.143 | 0.786 | 1.5 | 6.14 |
| eerlijk | honest | erl@k | QnIst | 0 | 0.55 | 1.25 | 6.38 |
| eerlijk | fair | erl@k | f8R | 0.143 | 0.72 | 1.38 | 7 |
| eerlijkheid | honesty | erl@khKt | QnIstI | 0 | 0.682 | 1.38 | 6.75 |
| eeuw | century | ew | sEnJUrI | 0.286 | 0.707 | 1.12 | 6.88 |
| eigenaar | owner | KG@nar | 5n@R | 0.25 | 0.775 | 2.25 | 6.88 |
| eigenschap | virtue | KG@sxAp | v3tju | 0.1 | 0.6 | 1.14 | 5 |
| eind | end | Knt | End | 0.75 | 0.984 | 5.5 | 6.88 |
| einde | end | Knd@ | End | 0.6 | 0.925 | 5.88 | 7 |
| eis | demand | Ks | dIm#nd | 0.167 | 0.7 | 1.12 | 6.38 |
| elementair | crude | el@mEnt)r | krud | 0.1 | 0.722 | 1.12 | 2.5 |
| elleboog | elbow | El@box | Elb5 | 0.5 | 0.859 | 5.62 | 7 |
| enorm | huge | enOrm | hju_ | 0 | 0.59 | 1.62 | 6.12 |
| enorm | gigantic | enOrm | _2g{ntIk | 0.125 | 0.706 | 1.25 | 6.88 |
| enorm | tremendous | enOrm | trImEnd@s | 0.3 | 0.75 | 1.75 | 6.62 |
| entree | entry | Entre | EntrI | 0.667 | 0.94 | 5.88 | 6.12 |
| erfenis | heritage | Erf@nIs | hErItI_ | 0.25 | 0.815 | 1.75 | 6.62 |
| erfenis | inheritance | Erf@nIs | InhErIt@ns | 0.273 | 0.825 | 1.75 | 6.75 |
| ervaring | experience | ErvarIN | Iksp7r7ns | 0.4 | 0.661 | 2.25 | 6.75 |
| erwt | pea | Ert | pi | 0 | 0.584 | 1.14 | 6.29 |
| evenement | event | ev@n@mEnt | IvEnt | 0.556 | 0.856 | 3.25 | 6 |
| fabriek | mill | fAbrik | mIl | 0.143 | 0.7 | 1 | 4.25 |
| fakkel | torch | fAk@l | t$J | 0 | 0.62 | 1.25 | 7 |
| fiets | cycle | fits | s2kP | 0 | 0.625 | 1 | 6.75 |
| fiets | bicycle | fits | b2sIkP | 0.143 | 0.7 | 1.5 | 6.38 |
| fiets | bike | fits | b2k | 0.2 | 0.613 | 1.38 | 5.88 |
| figuur | figure | fiGyr | fIg@R | 0.667 | 0.73 | 5.71 | 5 |
| film | movie | fIlm | muvI | 0 | 0.75 | 6.88 | 6.75 |
| film | film | fIlm | fIlm | 1 | 1 | 1.25 | 7 |
| fles | bottle | flEs | bQtP | 0.167 | 0.613 | 1.62 | 7 |
| formulier | form | fOrmylir | f$m | 0.444 | 0.838 | 4.62 | 6.62 |
| fruit | fruit | frLt | frut | 1 | 0.875 | 6.75 | 6.62 |
| gala | ball | xala | b$l | 0.5 | 0.688 | 1.38 | 6.38 |
| gangetje | alley | xAN@tj@ | {lI | 0.25 | 0.643 | 1.25 | 5.5 |
| gas | gas | xAs | g{s | 1 | 0.667 | 7 | 6.75 |
| gast | chap | xAst | J{p | 0 | 0.675 | 1.88 | 5.5 |
| gat | gap | xAt | g{p | 0.667 | 0.5 | 5 | 5.88 |
| gazon | lawn | xazOn | l$n | 0.4 | 0.79 | 1.62 | 6.62 |
| gebeurtenis | happening | x@b\|rt@nIs | h{pHIN | 0.182 | 0.745 | 1.25 | 7 |
| gebeurtenis | event | x@b\|rt@nIs | IvEnt | 0.273 | 0.72 | 1.12 | 6.86 |
| gebrek | deficiency | x@brEk | dIfISHsI | 0.2 | 0.588 | 1.25 | 6.75 |
| gebrek | lack | x@brEk | l{k | 0.167 | 0.75 | 1 | 5.88 |
| gebrek | flaw | x@brEk | fl$ | 0 | 0.641 | 2.12 | 6.88 |
| gebruiksvoorwerp | utensil | x@brLksforwErp | jutEnsP | 0.062 | 0.693 | 1.25 | 6.5 |
| gedachte | thought | x@dAxt@ | T$t | 0.25 | 0.728 | 2.88 | 6.88 |
| geduld | patience | x@d}lt | p1SHs | 0 | 0.591 | 1.29 | 6.86 |
| geheugen | memory | x@h\|G@ | mEm@rI | 0.125 | 0.617 | 1 | 6.88 |
| geld | money | xElt | mVnI | 0 | 0.563 | 1 | 7 |
| gelegenheid | opportunity | x@leG@hKt | Qp@tjun@tI | 0 | 0.675 | 1.25 | 6.5 |
| gelegenheid | occasion | x@leG@hKt | @k1ZH | 0 | 0.734 | 1.38 | 6.25 |
| geloof | believe | x@lof | bIliv | 0.286 | 0.7 | 1.25 | 6.5 |
| geloof | religion | x@lof | rIlI_@n | 0.375 | 0.657 | 1.5 | 6.38 |
| geloof | faith | x@lof | f1T | 0 | 0.7 | 1.38 | 7 |
| geluid | noise | x@lLt | n4z | 0 | 0.65 | 1.12 | 5.5 |
| geluk | luck | x@l}k | lVk | 0.4 | 0.8 | 1.12 | 6.88 |
| geluk | happiness | x@l}k | h{pInIs | 0 | 0.593 | 3.75 | 6.75 |
| gemak | ease | x@mAk | iz | 0.2 | 0.65 | 1.38 | 6.62 |
| gemeen | cruel | x@men | kr9l | 0.167 | 0.6 | 1.25 | 6.38 |
| gemeen | crude | x@men | krud | 0.167 | 0.6 | 1.25 | 6.75 |
| genade | pardon | x@nad@ | p#dH | 0 | 0.667 | 1.38 | 6.62 |
| genade | mercy | x@nad@ | m3sI | 0.167 | 0.617 | 1.12 | 4.5 |
| gerucht | rumour | x@r}xt | rum@R | 0.143 | 0.7 | 2.62 | 6.88 |
| geschenk | gift | x@sxENk | gIft | 0.125 | 0.679 | 1.62 | 6.62 |
| geur | smell | x\|r | smEl | 0.2 | 0.563 | 1.5 | 6.38 |
| gevaar | danger | x@var | d1n_@R | 0.167 | 0.7 | 1.25 | 7 |
| geval | case | x@vAl | k1s | 0 | 0.62 | 1.5 | 6.38 |
| gevangenis | jail | x@vAN@nIs | _1l | 0.2 | 0.739 | 1.12 | 7 |
| gevangenis | prison | x@vAN@nIs | prIzH | 0.1 | 0.75 | 1 | 7 |
| gevoel | feeling | x@vul | filIN | 0.143 | 0.7 | 2.88 | 6.5 |
| geweer | gun | x@wer | gVn | 0.167 | 0.68 | 2.5 | 6.5 |
| geweer | rifle | x@wer | r2fP | 0.167 | 0.65 | 1 | 6.62 |
| geweten | conscience | x@wet@ | kQnS@ns | 0.2 | 0.643 | 1 | 6.75 |
| gezicht | face | x@zIxt | f1s | 0.143 | 0.725 | 1.12 | 6.88 |
| gezondheid | health | x@zOnthKt | hElT | 0.2 | 0.745 | 1.5 | 7 |
| gift | gift | xIft | gIft | 1 | 0.875 | 6.88 | 5.25 |
| gigantisch | huge | xiGAntis | hju_ | 0.1 | 0.682 | 1.12 | 6.88 |
| gitaar | guitar | xitar | gIt#R | 0.667 | 0.73 | 6 | 7 |
| golf | wave | xOlf | w1v | 0 | 0.688 | 6.88 | 7 |
| golf | golf | xOlf | gQlf | 1 | 0.75 | 1.75 | 6.5 |
| goud | gold | xMt | g5ld | 0.75 | 0.738 | 5.62 | 7 |
| graaf | duke | xraf | djuk | 0 | 0.5 | 1 | 6.75 |
| graan | corn | xran | k$n | 0.2 | 0.688 | 1.38 | 6.12 |
| grap | joke | xrAp | _5k | 0 | 0.563 | 1.12 | 6.75 |
| grapje | joke | xrApj@ | _5k | 0.167 | 0.659 | 1.25 | 6.88 |
| grappig | funny | xrAp@x | fVnI | 0 | 0.625 | 1.38 | 6.75 |
| grasveld | lawn | xrAsfElt | l$n | 0.125 | 0.75 | 1.12 | 6.5 |
| griep | flu | xrip | flu | 0 | 0.563 | 1 | 7 |
| groenteboer | greengrocer | xrunt@bur | gringr5s@R | 0.545 | 0.72 | 3.25 | 6.5 |
| groenteman | greengrocer | xrunt@mAn | gringr5s@R | 0.364 | 0.695 | 3.88 | 6.25 |
| grond | floor | xrOnt | fl$R | 0.2 | 0.68 | 1.43 | 5.43 |
| grond | soil | xrOnt | s4l | 0.2 | 0.69 | 5.88 | 6.75 |
| grond | ground | xrOnt | gr6nd | 0.833 | 0.8 | 1.12 | 6.5 |
| grondbeginsel | principle | xrOndb@GIns@l | prIns@pP | 0.308 | 0.823 | 1.12 | 5.75 |
| groot | huge | xrot | hju_ | 0 | 0.613 | 1.38 | 6.25 |
| groot | tall | xrot | t$l | 0 | 0.688 | 1.62 | 5.75 |
| grootte | size | xrot@ | s2z | 0.143 | 0.6 | 1.12 | 6.88 |
| grot | cave | xrOt | k1v | 0 | 0.563 | 1.12 | 7 |
| grots | giant | NA | _2@nt | 0.2 | NA | 2.88 | 5.38 |
| gunst | favour | x}nst | f1v@R | 0 | 0.56 | 1.25 | 6.88 |
| haai | shark | haj | S#k | 0.4 | 0.5 | 1.62 | 7 |
| haar | hair | har | h8R | 0.75 | 0.817 | 5.25 | 6.88 |
| hals | throat | hAls | Tr5t | 0.167 | 0.563 | 1.38 | 3.88 |
| hand | hand | hAnt | h{nd | 1 | 0.875 | 7 | 6.88 |
| handdoek | towel | hAnduk | t6@l | 0.125 | 0.692 | 1.25 | 7 |
| handel | trade | hAnd@l | tr1d | 0.333 | 0.709 | 1.38 | 6.38 |
| handschoen | glove | hAntsxun | glVv | 0.1 | 0.625 | 1 | 7 |
| handtekening | autograph | hAntek@nIN | $t@gr#f | 0.167 | 0.71 | 1.12 | 5.88 |
| handtekening | signature | hAntek@nIN | sIgn@J@R | 0.167 | 0.715 | 1.12 | 7 |
| hard | tough | hArt | tVf | 0 | 0.688 | 1.38 | 5.75 |
| hardheid | cruelty | hArthKt | kr9ltI | 0.125 | 0.707 | 1 | 5.25 |
| hart | heart | hArt | h#t | 0.8 | 0.925 | 5 | 6.88 |
| hartstocht | passion | hArtstOxt | p{SH | 0.3 | 0.689 | 1.38 | 6.88 |
| haven | haven | hav@ | h1vH | 1 | 0.75 | 6.88 | 4.5 |
| haver | oat | hav@r | 5t | 0.2 | 0.69 | 1 | 6.75 |
| heer | sir | her | s3R | 0.25 | 0.65 | 1 | 6.62 |
| heer | gentleman | her | _EntPm@n | 0.222 | 0.7 | 1.38 | 6.38 |
| heg | hedge | hEx | hE_ | 0.6 | 0.834 | 4.5 | 6 |
| heiden | pagan | hKd@ | p1g@n | 0.167 | 0.69 | 3.71 | 6 |
| heiden | heathen | hKd@ | hiDH | 0.571 | 0.775 | 1.43 | 5.57 |
| heilig | sacred | hKl@x | s1krId | 0 | 0.575 | 4.25 | 7 |
| heilig | holy | hKl@x | h5lI | 0.333 | 0.75 | 1.38 | 6.62 |
| hel | hell | hEl | hEl | 0.75 | 1 | 6.62 | 7 |
| held | hero | hElt | h7r5 | 0.5 | 0.625 | 4.88 | 7 |
| hemel | sky | hem@l | sk2 | 0 | 0.64 | 1.75 | 5.62 |
| herfst | fall | hErfst | f$l | 0 | 0.709 | 1.12 | 6.88 |
| herfst | autumn | hErfst | $t@m | 0 | 0.667 | 1.25 | 6.62 |
| herinnering | memory | hErIn@rIN | mEm@rI | 0.182 | 0.834 | 1.5 | 6.12 |
| herstel | recovery | hErstEl | rIkVv@rI | 0.25 | 0.612 | 1.75 | 6.5 |
| herstellen | recovery | hErstEl@ | rIkVv@rI | 0.2 | 0.625 | 2.12 | 6.25 |
| hertog | duke | hErtOx | djuk | 0 | 0.667 | 1.12 | 6.62 |
| hitte | heat | hIt@ | hit | 0.4 | 0.863 | 5.75 | 6.88 |
| hoed | cap | hut | k{p | 0 | 0.5 | 1.12 | 5.25 |
| hoed | hat | hut | h{t | 0.25 | 0.834 | 5.5 | 6.75 |
| hoek | corner | huk | k$n@R | 0.333 | 0.7 | 1.12 | 6.88 |
| hoek | angle | huk | {NgP | 0 | 0.688 | 1.12 | 6.88 |
| hol | hollow | hOl | hQl5 | 0.5 | 0.813 | 1.38 | 6 |
| hol | cave | hOl | k1v | 0 | 0.5 | 5.25 | 6.12 |
| hond | dog | hOnt | dQg | 0.25 | 0.688 | 1.75 | 7 |
| honing | honey | honIN | hVnI | 0.5 | 0.85 | 5.25 | 6.88 |
| hoofd | master | hoft | m#st@R | 0 | 0.667 | 4.25 | 6.88 |
| hoofd | head | hoft | hEd | 0.4 | 0.813 | 1.12 | 4.5 |
| hoogte | height | hoxt@ | h2t | 0.333 | 0.8 | 4.62 | 5.75 |
| horloge | watch | hOrloZ@ | wQJ | 0 | 0.707 | 1.12 | 6.88 |
| hout | wood | hMt | wUd | 0.25 | 0.734 | 1.75 | 6.62 |
| huid | skin | hLt | skIn | 0.25 | 0.675 | 1.12 | 6.75 |
| huidig | current | hLd@x | kVr@nt | 0.143 | 0.641 | 1.25 | 6.12 |
| huis | house | hLs | h6s | 0.4 | 0.834 | 5.38 | 7 |
| huishoudhulp | maid | hLshMth}lp | m1d | 0.167 | 0.745 | 1 | 5.75 |
| huurder | renter | hyrd@r | rEnt@R | 0.286 | 0.825 | 1.75 | 6.75 |
| huurder | tenant | hyrd@r | tEn@nt | 0 | 0.709 | 1.14 | 6.57 |
| ijzer | iron | Kz@r | 2@n | 0.2 | 0.8 | 3.12 | 6.62 |
| impressie | impression | ImprEsi | ImprESH | 0.8 | 0.893 | 5.88 | 6.75 |
| indruk | impression | Indr}k | ImprESH | 0.2 | 0.679 | 2.25 | 6.62 |
| influentie | influence | InflywEnsi | Infl9ns | 0.8 | 0.875 | 6.12 | 4.88 |
| informatie | information | InfOrmatsi | Inf@m1SH | 0.818 | 0.79 | 5.62 | 7 |
| ingang | entry | INGAN | EntrI | 0.167 | 0.6 | 3.25 | 6.88 |
| ingang | entrance | INGAN | Entr@ns | 0.375 | 0.572 | 1.75 | 6.75 |
| inhoud | volume | InhMt | vQljum | 0 | 0.609 | 1.38 | 5.88 |
| inhoud | contents | InhMt | kQntEnts | 0.125 | 0.719 | 1.43 | 6.71 |
| inhoud | content | InhMt | kQntEnt | 0.143 | 0.715 | 1.25 | 5.62 |
| inkt | ink | INkt | INk | 0.75 | 0.938 | 6.12 | 7 |
| insekt | insect | InsEkt | InsEkt | 0.833 | 1 | 6.57 | 7 |
| interesse | interest | Int@rEs@ | Intr@st | 0.778 | 0.862 | 4.75 | 5.88 |
| invloed | influence | Invlut | Infl9ns | 0.444 | 0.879 | 5 | 6.75 |
| inwoner | citizen | Inwon@r | sItIzH | 0.143 | 0.707 | 1.25 | 5.62 |
| inwoner | resident | Inwon@r | rEzId@nt | 0.125 | 0.719 | 2.25 | 6.75 |
| inwoner | inhabitant | Inwon@r | Inh{bIt@nt | 0.2 | 0.775 | 1.25 | 6.5 |
| inzicht | understanding | InzIxt | Vnd@st{ndIN | 0.154 | 0.75 | 5.75 | 6 |
| inzicht | insight | InzIxt | Ins2t | 0.714 | 0.95 | 1.12 | 6.12 |
| jaar | year | jar | j7R | 0.5 | 0.817 | 5.88 | 7 |
| jammer | pity | jAm@r | pItI | 0 | 0.6 | 1.12 | 6.62 |
| japon | gown | japOn | g6n | 0.2 | 0.78 | 1.25 | 6 |
| japon | dress | japOn | drEs | 0 | 0.6 | 1.25 | 6.62 |
| jas | jacket | jAs | _{kIt | 0.333 | 0.69 | 1.25 | 6.5 |
| jas | coat | jAs | k5t | 0.25 | 0.5 | 3.25 | 5.25 |
| jeugd | youth | j\|xt | juT | 0.2 | 0.688 | 5 | 6.62 |
| jongen | lad | jON@ | l{d | 0 | 0.563 | 1.38 | 6.88 |
| jongen | boy | jON@ | b4 | 0.167 | 0.738 | 1 | 6.25 |
| jurk | gown | j}rk | g6n | 0 | 0.688 | 1.25 | 7 |
| jurk | dress | j}rk | drEs | 0 | 0.625 | 1.25 | 6.25 |
| kaak | jaw | kak | _$ | 0.25 | 0.584 | 1.25 | 6.88 |
| kaars | candle | kars | k{ndP | 0.167 | 0.74 | 3.62 | 7 |
| kaart | map | kart | m{p | 0.2 | 0.613 | 1.12 | 6.88 |
| kaartje | postcard | kartj@ | p5stk#d | 0 | 0.679 | 4.5 | 6.5 |
| kaartje | ticket | kartj@ | tIkIt | 0 | 0.709 | 2.62 | 6.38 |
| kaartje | tag | kartj@ | t{g | 0.143 | 0.709 | 1.25 | 5.25 |
| kaartje | card | kartj@ | k#d | 0.286 | 0.792 | 2.12 | 6.75 |
| kado | gift | NA | gIft | 0 | NA | 1.5 | 6.5 |
| kalf | calf | kAlf | k#f | 0.75 | 0.925 | 5.75 | 7 |
| kamer | room | kam@r | rum | 0 | 0.7 | 1.12 | 6.88 |
| kans | opportunity | kAns | Qp@tjun@tI | 0.091 | 0.75 | 5.5 | 6.88 |
| kans | chance | kAns | J#ns | 0.333 | 0.863 | 1.62 | 6.38 |
| kantoor | office | kAntor | QfIs | 0 | 0.667 | 1.25 | 6.88 |
| kapitein | captain | kApitKn | k{ptIn | 0.625 | 0.85 | 5.88 | 6.62 |
| kast | chest | kAst | JEst | 0.4 | 0.75 | 4 | 4.12 |
| kat | cat | kAt | k{t | 0.667 | 0.834 | 6.25 | 7 |
| katoen | cotton | katun | kQtH | 0.333 | 0.84 | 5 | 7 |
| keel | throat | kel | Tr5t | 0 | 0.6 | 1.75 | 6.5 |
| kerel | lad | ker@l | l{d | 0 | 0.7 | 1.38 | 6.5 |
| kerel | guy | ker@l | g2 | 0 | 0.78 | 1 | 6.38 |
| kerel | dude | ker@l | djud | 0.2 | 0.55 | 1.62 | 6.12 |
| kerel | chap | ker@l | J{p | 0 | 0.6 | 1.12 | 6.5 |
| kerel | fellow | ker@l | fEl5 | 0.167 | 0.68 | 1.25 | 6.5 |
| keten | string | ket@ | strIN | 0.167 | 0.65 | 1.88 | 6.75 |
| keten | chain | ket@ | J1n | 0.2 | 0.725 | 1.12 | 4.75 |
| ketting | chain | kEtIN | J1n | 0.286 | 0.78 | 1.25 | 6.88 |
| keuken | kitchen | k\|k@ | kIJIn | 0.429 | 0.69 | 4.12 | 7 |
| keus | choice | k\|s | J4s | 0 | 0.717 | 2 | 6.75 |
| keuze | choice | k\|z@ | J4s | 0.167 | 0.725 | 3.62 | 6.75 |
| kijken | watch | kKk@ | wQJ | 0 | 0.563 | 1.25 | 6.75 |
| kijkt | watch | NA | wQJ | 0 | NA | 1.25 | 5.25 |
| kikker | frog | kIk@r | frQg | 0 | 0.65 | 1.62 | 6.88 |
| kin | chin | kIn | JIn | 0.5 | 0.834 | 6 | 6.75 |
| klacht | complaint | klAxt | k@mpl1nt | 0.333 | 0.781 | 1.88 | 7 |
| kleur | colour | kl\|r | kVl@R | 0.5 | 0.86 | 3.5 | 7 |
| klimaat | climate | klimat | kl2mIt | 0.571 | 0.859 | 5.75 | 6.88 |
| klok | clock | klOk | klQk | 0.6 | 0.875 | 5.75 | 7 |
| kloof | canyon | klof | k{nj@n | 0.167 | 0.683 | 2.12 | 5.88 |
| kloof | gorge | klof | g$_ | 0 | 0.688 | 1.12 | 5.75 |
| kloof | gap | klof | g{p | 0 | 0.688 | 1.71 | 5.43 |
| knoflook | garlic | knOflok | g#lIk | 0.125 | 0.786 | 1.38 | 6.62 |
| knul | lad | kn}l | l{d | 0 | 0.688 | 1 | 6.5 |
| koe | cow | ku | k6 | 0.333 | 0.825 | 3.75 | 7 |
| kofferbak | trunk | kOf@rbAk | trVNk | 0.222 | 0.719 | 1.12 | 6.88 |
| koffie | coffee | kOfi | kQfI | 0.667 | 0.8 | 6 | 7 |
| kogel | bullet | koG@l | bUlIt | 0.167 | 0.64 | 1.75 | 7 |
| komst | arrival | kOmst | @r2vP | 0 | 0.57 | 1.12 | 6 |
| konijn | rabbit | konKn | r{bIt | 0 | 0.64 | 1.25 | 6.88 |
| koning | king | konIN | kIN | 0.667 | 0.9 | 4.88 | 7 |
| koningin | queen | konINIn | kwin | 0.125 | 0.779 | 3.38 | 6.75 |
| kooi | cage | koj | k1_ | 0 | 0.667 | 3.5 | 6.88 |
| koorts | fever | korts | fiv@R | 0 | 0.61 | 1.12 | 7 |
| koren | corn | kor@ | k$n | 0.6 | 0.75 | 4.88 | 2.75 |
| kostuum | costume | kOstym | kQstjUm | 0.571 | 0.822 | 5.38 | 6.38 |
| kostuum | suit | kOstym | sut | 0.286 | 0.792 | 1.25 | 6.38 |
| kraan | tap | kran | t{p | 0.2 | 0.625 | 1 | 6 |
| kraan | faucet | kran | f$sIt | 0 | 0.6 | 1.12 | 6.75 |
| kracht | force | krAxt | f$s | 0.167 | 0.6 | 1.38 | 6.25 |
| kracht | strength | krAxt | strENT | 0.25 | 0.625 | 1.25 | 6.62 |
| kracht | power | krAxt | p6@R | 0 | 0.64 | 1.5 | 6.62 |
| krant | paper | krAnt | p1p@R | 0 | 0.59 | 1.12 | 7 |
| krant | newspaper | krAnt | njusp1p@R | 0.111 | 0.667 | 1.25 | 6.38 |
| kritiek | criticism | kritik | krItIsIz@m | 0.444 | 0.79 | 4.25 | 6.62 |
| kroon | crown | kron | kr6n | 0.6 | 0.913 | 5.12 | 6.88 |
| kruid | herb | krLt | h3b | 0 | 0.613 | 1.25 | 6.12 |
| kuit | calf | kLt | k#f | 0 | 0.667 | 2.25 | 3.62 |
| kunst | art | k}nst | #t | 0.2 | 0.75 | 1.25 | 6.88 |
| kussen | pillow | k}s@ | pIl5 | 0 | 0.587 | 1.12 | 7 |
| kwaad | anger | kwat | {Ng@R | 0 | 0.65 | 1.25 | 6.25 |
| kwaadheid | anger | kwathKt | {Ng@R | 0.222 | 0.657 | 1.5 | 6.5 |
| kwaliteit | quality | kwalitKt | kwQl@tI | 0.444 | 0.806 | 5.38 | 7 |
| laars | boot | lars | but | 0 | 0.563 | 1.38 | 6.5 |
| lafaard | coward | lAfart | k6@d | 0.429 | 0.734 | 1.38 | 6.75 |
| laken | sheet | lak@ | Sit | 0.2 | 0.563 | 1.38 | 6.62 |
| lam | lamb | lAm | l{m | 0.75 | 0.834 | 6.14 | 6.57 |
| lammetje | lamb | NA | l{m | 0.375 | NA | 4.25 | 7 |
| land | land | lAnt | l{nd | 1 | 0.875 | 1.12 | 6.62 |
| land | country | lAnt | kVntrI | 0.143 | 0.75 | 6.75 | 6.25 |
| landkaart | map | lAntkart | m{p | 0.111 | 0.682 | 1.5 | 6.75 |
| lang | tall | lAN | t$l | 0.25 | 0.667 | 5.25 | 6.5 |
| lang | long | lAN | lQN | 0.75 | 1 | 1.75 | 6.5 |
| lawaai | noise | lawaj | n4z | 0 | 0.6 | 1.12 | 6.5 |
| leeftijd | age | leftKt | 1_ | 0.125 | 0.809 | 1.12 | 6.88 |
| leen | loan | len | l5n | 0.5 | 0.884 | 5.25 | 5.88 |
| leeuw | lion | lew | l2@n | 0.2 | 0.738 | 2.88 | 7 |
| leger | army | leG@r | #mI | 0 | 0.64 | 1.25 | 6.88 |
| leider | leader | lKd@r | lid@R | 0.833 | 0.97 | 6 | 6.88 |
| lening | loan | lenIN | l5n | 0.333 | 0.83 | 4.29 | 7 |
| lens | lens | lEns | lEnz | 1 | 1 | 7 | 6.88 |
| lepel | spoon | lep@l | spun | 0 | 0.65 | 1.12 | 7 |
| les | lesson | lEs | lEsH | 0.5 | 0.938 | 4.12 | 6.75 |
| leugen | lie | l\|G@ | l2 | 0.333 | 0.788 | 2.12 | 6.88 |
| leuk | nice | l\|k | n2s | 0 | 0.55 | 1.38 | 6 |
| leuk | funny | l\|k | fVnI | 0 | 0.613 | 1.25 | 6.5 |
| lichaam | body | lIxam | bQdI | 0 | 0.65 | 1 | 6.75 |
| lied | song | lit | sQN | 0 | 0.5 | 1.25 | 6.88 |
| liedje | song | litj@ | sQN | 0 | 0.6 | 1.57 | 6.57 |
| lijf | body | lKf | bQdI | 0 | 0.613 | 1.38 | 6.38 |
| litteken | scar | lItek@ | sk#R | 0 | 0.667 | 1.12 | 6.88 |
| loon | salary | lon | s{l@rI | 0.167 | 0.766 | 1 | 6.38 |
| loon | payment | lon | p1m@nt | 0.143 | 0.725 | 1.25 | 6.5 |
| loon | wages | lon | w1_Iz | 0 | 0.64 | 1 | 6.38 |
| loon | wage | lon | w1_ | 0 | 0.567 | 1.12 | 6.5 |
| lot | fate | lOt | f1t | 0.25 | 0.667 | 1.62 | 6.62 |
| lucht | sky | l}xt | sk2 | 0 | 0.587 | 1.12 | 6.75 |
| lucht | air | l}xt | 8R | 0 | 0.625 | 1 | 7 |
| maag | stomach | max | stVm@k | 0.286 | 0.709 | 1.25 | 6.88 |
| maan | moon | man | mun | 0.5 | 0.834 | 5.38 | 7 |
| maat | size | mat | s2z | 0 | 0.634 | 1.12 | 6.25 |
| macht | power | mAxt | p6@R | 0 | 0.563 | 1.12 | 6.5 |
| mais | corn | NA | k$n | 0 | NA | 1.12 | 6.88 |
| manier | mode | manir | m5d | 0.333 | 0.75 | 5.5 | 6.5 |
| manier | manner | manir | m{n@R | 0.833 | 0.83 | 2.12 | 5.62 |
| manier | way | manir | w1 | 0.167 | 0.72 | 1.25 | 6.25 |
| masker | mask | mAsk@r | m#sk | 0.667 | 0.909 | 5.88 | 7 |
| mast | mast | mAst | m#st | 1 | 0.988 | 6.88 | 5.5 |
| medaille | medal | medAj@ | mEdP | 0.625 | 0.75 | 4.62 | 6.75 |
| medelijden | mercy | med@lKd@ | m3sI | 0.2 | 0.738 | 2.12 | 6.5 |
| medelijden | pity | med@lKd@ | pItI | 0.1 | 0.769 | 1.12 | 6.5 |
| meelij | pity | melK | pItI | 0 | 0.663 | 1.12 | 6.62 |
| meerderheid | majority | merd@rhKt | m@_Qr@tI | 0.182 | 0.766 | 3.62 | 6.88 |
| meester | master | mest@r | m#st@R | 0.714 | 0.909 | 6 | 6.5 |
| meid | chick | mKt | JIk | 0.2 | 0.65 | 1.25 | 4.88 |
| meid | maid | mKt | m1d | 0.75 | 0.9 | 1.12 | 5.75 |
| meid | girl | mKt | g3l | 0 | 0.55 | 6.12 | 3.88 |
| meisje | girl | mKsj@ | g3l | 0.167 | 0.64 | 1.25 | 7 |
| meneer | gentleman | m@ner | _EntPm@n | 0.333 | 0.781 | 2 | 6.25 |
| menigte | crowd | men@xt@ | kr6d | 0 | 0.715 | 1.12 | 6.62 |
| mening | meaning | menIN | minIN | 0.857 | 0.9 | 5.88 | 3.62 |
| mening | opinion | menIN | @pInj@n | 0.286 | 0.707 | 1.38 | 6.88 |
| mensen | people | NA | pipP | 0.167 | NA | 1.25 | 6.62 |
| mes | knife | mEs | n2f | 0 | 0.567 | 1.12 | 7 |
| metaal | metal | metal | mEtP | 0.833 | 0.84 | 5.88 | 6.88 |
| methode | method | metod@ | mET@d | 0.857 | 0.725 | 5.88 | 7 |
| middelpunt | middle | mId@lp}nt | mIdP | 0.5 | 0.856 | 4.88 | 5.75 |
| middelste | middle | NA | mIdP | 0.667 | NA | 4.5 | 6.25 |
| midden | middle | mId@ | mIdP | 0.667 | 0.875 | 5 | 6.62 |
| mier | ant | mir | {nt | 0 | 0.584 | 1.38 | 7 |
| mijl | mile | mKl | m2l | 0.5 | 0.9 | 6.25 | 6.62 |
| misbruik | abuse | mIzbrLk | @bjus | 0.25 | 0.679 | 1.29 | 6.29 |
| misdaad | crime | mIzdat | kr2m | 0 | 0.667 | 1.12 | 6.62 |
| misdadiger | crook | mIzdad@G@r | krUk | 0 | 0.7 | 1.12 | 5.88 |
| mislukking | failure | mIsl}kIN | f1lj@R | 0.2 | 0.682 | 1.38 | 6.38 |
| mist | mist | mIst | mIst | 1 | 1 | 1.62 | 6.5 |
| mist | fog | mIst | fQg | 0 | 0.563 | 7 | 7 |
| modder | mud | mOd@r | mVd | 0.333 | 0.9 | 4.25 | 7 |
| mode | mode | mod@ | m5d | 1 | 0.85 | 1.12 | 7 |
| mode | fashion | mod@ | f{SH | 0 | 0.55 | 6.88 | 4.88 |
| modus | mode | mod}s | m5d | 0.6 | 0.83 | 5.43 | 5.57 |
| moeder | mother | mud@r | mVD@R | 0.667 | 0.79 | 5.88 | 7 |
| moedig | bold | mud@x | b5ld | 0.333 | 0.73 | 1.12 | 5.12 |
| mogelijkheid | opportunity | moG@l@khKt | Qp@tjun@tI | 0.083 | 0.675 | 1.5 | 5.62 |
| mogelijkheid | chance | moG@l@khKt | J#ns | 0.083 | 0.695 | 1.75 | 6.25 |
| mogelijkheid | possibility | moG@l@khKt | pQs@bIl@tI | 0.167 | 0.725 | 2 | 6.88 |
| molen | windmill | mol@ | wInmIl | 0.125 | 0.75 | 3.62 | 7 |
| molen | mill | mol@ | mIl | 0.4 | 0.813 | 1.88 | 5.62 |
| mond | mouth | mOnt | m6T | 0.4 | 0.75 | 4.25 | 6.88 |
| monnik | monk | mOn@k | mVNk | 0.667 | 0.85 | 5.5 | 6.88 |
| mooi | fair | moj | f8R | 0 | 0.55 | 1.38 | 4.88 |
| moordenaar | murderer | mord@nar | m3d@r@R | 0.5 | 0.806 | 4.25 | 6.88 |
| motief | motive | motif | m5tIv | 0.667 | 0.87 | 5.88 | 6.25 |
| mouw | sleeve | mMw | sliv | 0 | 0.563 | 1.12 | 6.88 |
| muildier | mule | mLldir | mjul | 0.5 | 0.75 | 4.71 | 6.43 |
| muis | mouse | mLs | m6s | 0.4 | 0.834 | 4.38 | 7 |
| munt | coin | m}nt | k4n | 0 | 0.738 | 1.12 | 7 |
| muntje | coin | m}ntj@ | k4n | 0 | 0.742 | 1.12 | 7 |
| muur | wall | myr | w$l | 0 | 0.5 | 1.5 | 6.88 |
| muziek | music | myzik | mjuzIk | 0.5 | 0.825 | 5.25 | 7 |
| naald | needle | nalt | nidP | 0.333 | 0.75 | 4.25 | 7 |
| naam | name | nam | n1m | 0.5 | 0.834 | 6.12 | 6.88 |
| nacht | night | nAxt | n2t | 0.6 | 0.813 | 4.75 | 6.75 |
| nadeel | disadvantage | nadel | dIs@dv#ntI_ | 0.167 | 0.746 | 1 | 6.88 |
| nat | wet | nAt | wEt | 0.333 | 0.667 | 1.88 | 7 |
| natuur | nature | natyr | n1J@R | 0.667 | 0.78 | 6 | 6.62 |
| nederlaag | defeat | ned@rlax | dIfit | 0.333 | 0.713 | 1.12 | 6.62 |
| neger | negro | neG@r | NA | 0.6 | NA | 5.12 | 6.38 |
| nek | neck | nEk | nEk | 0.75 | 1 | 6.25 | 7 |
| neus | nose | n\|s | n5z | 0.25 | 0.884 | 4.38 | 7 |
| nobel | noble | nob@l | n5bP | 0.6 | 0.87 | 6.25 | 6.62 |
| nonsens | nonsense | nOns@ns | nQns@ns | 0.875 | 0.929 | 6.43 | 6.71 |
| noodlot | fate | nodlOt | f1t | 0 | 0.709 | 1.12 | 6 |
| noodlot | destiny | nodlOt | dEstInI | 0 | 0.679 | 1.25 | 6.25 |
| noodzaak | need | notsak | nid | 0.25 | 0.792 | 2 | 6.38 |
| noodzaak | necessity | notsak | nIsEs@tI | 0.111 | 0.7 | 2.5 | 6 |
| noodzakelijkheid | necessity | notsak@l@khKt | nIsEs@tI | 0.125 | 0.762 | 2.12 | 6.75 |
| ochtend | dawn | Oxt@nt | d$n | 0.143 | 0.808 | 1.25 | 5.62 |
| ochtendschemer | dawn | NA | d$n | 0.071 | NA | 1.12 | 6.38 |
| offer | offer | Of@r | Qf@R | 1 | 0.863 | 6.88 | 3.25 |
| offer | sacrifice | Of@r | s{krIf2s | 0.111 | 0.688 | 1.25 | 6.75 |
| onderbroek | pants | Ond@rbruk | p{nts | 0 | 0.722 | 1 | 3.75 |
| onderwijs | education | Ond@rwKs | E_Uk1SH | 0.111 | 0.644 | 1.38 | 6.88 |
| oneven | unequal | Onev@ | Vnikw@l | 0.286 | 0.786 | 1.38 | 4.25 |
| oneven | irregular | Onev@ | IrEgjUl@R | 0.111 | 0.695 | 1.5 | 6.12 |
| oneven | uneven | Onev@ | VnivH | 0.833 | 0.8 | 2.12 | 5.12 |
| oneven | odd | Onev@ | Qd | 0.167 | 0.7 | 5.12 | 6.38 |
| onlust | riot | Onl}st | r2@t | 0.167 | 0.725 | 1.38 | 4 |
| onschuld | innocence | Onsx}lt | In@s@ns | 0.222 | 0.679 | 1.5 | 7 |
| ontdekking | discovery | OndEkIN | dIskVv@rI | 0 | 0.722 | 1.75 | 6.75 |
| ontmoeting | meeting | OntmutIN | mitIN | 0.6 | 0.844 | 3.38 | 6.5 |
| onzin | nonsense | OnzIn | nQns@ns | 0.375 | 0.786 | 1.62 | 7 |
| oog | eye | ox | 2 | 0 | 0.625 | 4 | 7 |
| oom | uncle | om | VNkP | 0 | 0.625 | 2.62 | 7 |
| oor | ear | or | 7R | 0.333 | 0.8 | 4.25 | 7 |
| oorlog | war | orlOx | w$R | 0.167 | 0.69 | 1.25 | 7 |
| oorzaak | cause | orzak | k$z | 0 | 0.7 | 1.38 | 6.75 |
| opdracht | task | ObdrAxt | t#sk | 0.125 | 0.743 | 1.25 | 6.38 |
| openbaar | public | op@bar | pVblIk | 0.25 | 0.691 | 1.12 | 6.75 |
| opening | gap | op@nIN | g{p | 0 | 0.709 | 1 | 6 |
| opinie | opinion | opini | @pInj@n | 0.714 | 0.757 | 5.62 | 6 |
| opleiding | education | OplKdIN | E_Uk1SH | 0.111 | 0.693 | 1.5 | 6.75 |
| oplichter | conman | OplIxt@r | NA | 0 | NA | 1.38 | 6.5 |
| oplichter | crook | OplIxt@r | krUk | 0.111 | 0.688 | 1.25 | 6.12 |
| oplossing | solution | OplOsIN | s@luSH | 0.222 | 0.664 | 1.75 | 6.5 |
| opofferen | sacrifice | OpOf@r@ | s{krIf2s | 0.111 | 0.594 | 1.25 | 6.75 |
| opoffering | sacrifice | OpOf@rIN | s{krIf2s | 0.1 | 0.625 | 1.25 | 6.5 |
| opstand | riot | OpstAnt | r2@t | 0.143 | 0.715 | 1.25 | 6.12 |
| order | order | Ord@r | $d@R | 1 | 0.93 | 6.75 | 5.38 |
| overeenkomst | treaty | ov@reNkOmst | tritI | 0.167 | 0.728 | 1 | 6.12 |
| overjas | cloak | ov@rjAs | kl5k | 0.143 | 0.636 | 1.43 | 5.14 |
| overstroming | flood | ov@rstromIN | flVd | 0.083 | 0.764 | 1.38 | 6.62 |
| paal | pole | pal | p5l | 0.25 | 0.834 | 4.62 | 5.75 |
| paar | couple | par | kVpP | 0 | 0.688 | 1 | 6.57 |
| paar | pair | par | p8R | 0.75 | 0.817 | 5.88 | 6.5 |
| paard | horse | part | h$s | 0 | 0.563 | 1.25 | 7 |
| pak | suit | pAk | sut | 0 | 0.5 | 1.14 | 6.71 |
| paniek | panic | panik | p{nIk | 0.667 | 0.88 | 5.5 | 6.75 |
| papier | sheet | papir | Sit | 0.167 | 0.7 | 1.12 | 5.62 |
| paraplu | umbrella | paraply | VmbrEl@ | 0.25 | 0.715 | 1.25 | 6.88 |
| parel | pearl | par@l | p3l | 0.6 | 0.84 | 5.5 | 7 |
| passie | passion | pAsi | p{SH | 0.714 | 0.688 | 4.88 | 7 |
| paus | pope | pMs | p5p | 0.25 | 0.734 | 2.75 | 6.88 |
| peer | pear | per | p8R | 0.75 | 0.867 | 5.88 | 6.88 |
| pen | pen | pEn | pEn | 1 | 1 | 7 | 6.75 |
| peper | pepper | pep@r | pEp@R | 0.833 | 0.89 | 6 | 7 |
| perzik | peach | pErzIk | piJ | 0.333 | 0.742 | 3.38 | 7 |
| pijl | arrow | pKl | {r5 | 0 | 0.567 | 1.12 | 6.75 |
| pijp | pipe | pKp | p2p | 0.5 | 0.9 | 5.88 | 6.62 |
| pin | pin | pIn | pIn | 1 | 1 | 7 | 6 |
| piraat | pirate | pirat | p2@r@t | 0.667 | 0.817 | 5.25 | 6.88 |
| pistool | gun | pistol | gVn | 0 | 0.667 | 1 | 6.75 |
| pistool | pistol | pistol | pIstP | 0.857 | 0.9 | 6.12 | 6.75 |
| plaats | town | plats | t6n | 0 | 0.73 | 1.25 | 5.38 |
| plaatsbewijs | ticket | pladzb@wKs | tIkIt | 0.083 | 0.695 | 1.12 | 5.75 |
| plafond | ceiling | plafOnt | silIN | 0.143 | 0.672 | 1.12 | 7 |
| plan | idea | plAn | 2d7 | 0 | 0.625 | 1.12 | 5.12 |
| plan | plan | plAn | pl{n | 1 | 0.875 | 7 | 7 |
| plattegrond | map | plAt@GrOnt | m{p | 0.091 | 0.725 | 1 | 6.75 |
| plezier | joy | pl@zir | _4 | 0 | 0.767 | 1.38 | 6.5 |
| plicht | duty | plIxt | djutI | 0 | 0.6 | 1 | 6.5 |
| poging | effort | poGIN | Ef@t | 0 | 0.57 | 1.12 | 6.75 |
| poging | try | poGIN | tr2 | 0 | 0.69 | 1.25 | 6 |
| poging | attempt | poGIN | @tEmpt | 0 | 0.625 | 1 | 5.88 |
| pokken | pox | pOk@ | pQks | 0.333 | 0.75 | 5.38 | 6.62 |
| politie | police | politsi | p@lis | 0.714 | 0.871 | 5.38 | 6.88 |
| pond | pound | pOnt | p6nd | 0.8 | 0.875 | 6.14 | 6 |
| pool | pole | pol | p5l | 0.5 | 0.884 | 5.75 | 4.75 |
| pop | puppet | pOp | pVpIt | 0.333 | 0.9 | 1.75 | 6.88 |
| pop | doll | pOp | dQl | 0.25 | 0.5 | 3.62 | 6.88 |
| post | post | pOst | p5st | 1 | 0.875 | 1.12 | 7 |
| post | mail | pOst | m1l | 0 | 0.625 | 6.75 | 3 |
| pot | pot | pOt | pQt | 1 | 0.834 | 1.75 | 6.5 |
| pot | jar | pOt | _#R | 0 | 0.65 | 7 | 5 |
| potlood | pencil | pOtlot | pEnsP | 0.143 | 0.742 | 2.25 | 6.88 |
| preek | speech | prek | spiJ | 0.333 | 0.625 | 2.75 | 4.88 |
| prijs | prize | prKs | pr2z | 0.6 | 0.925 | 5.62 | 7 |
| prijs | price | prKs | pr2s | 0.6 | 0.925 | 5.43 | 6.86 |
| principe | principle | prInsip@ | prIns@pP | 0.889 | 0.875 | 6.12 | 6.25 |
| prins | prince | prIns | prIns | 0.667 | 1 | 6.25 | 6.88 |
| publiek | crowd | pyblik | kr6d | 0 | 0.667 | 1 | 6.88 |
| publiek | audience | pyblik | $dj@ns | 0.25 | 0.5 | 1.12 | 6.38 |
| publiek | public | pyblik | pVblIk | 0.714 | 0.867 | 5.5 | 6.75 |
| publiekelijk | public | pyblik@l@k | pVblIk | 0.417 | 0.82 | 4.25 | 6.75 |
| raadsel | riddle | rats@l | rIdP | 0.429 | 0.825 | 4.5 | 6.75 |
| raam | window | ram | wInd5 | 0 | 0.69 | 1 | 7 |
| raar | odd | rar | Qd | 0 | 0.584 | 1.5 | 6 |
| rail | rails | rel | r1lz | 0.8 | 0.913 | 6.88 | 6.75 |
| rail | rail | rel | r1l | 1 | 0.967 | 5.88 | 6.5 |
| rauw | raw | rMw | r$ | 0.75 | 0.75 | 4.75 | 6.38 |
| recent | current | r@sEnt | kVr@nt | 0.429 | 0.834 | 2.25 | 6 |
| rechten | law | rExt@ | l$ | 0 | 0.67 | 1 | 5.75 |
| reden | cause | red@ | k$z | 0 | 0.587 | 1.12 | 6.38 |
| reden | reason | red@ | rizH | 0.5 | 0.675 | 3 | 6.88 |
| regel | sentence | reG@l | sEnt@ns | 0.25 | 0.679 | 1.5 | 6.5 |
| regel | rule | reG@l | rul | 0.4 | 0.8 | 4.75 | 6.62 |
| regel | line | reG@l | l2n | 0.2 | 0.7 | 1.62 | 4.88 |
| regen | rain | reG@ | r1n | 0.4 | 0.788 | 3.25 | 6.88 |
| rel | riot | rEl | r2@t | 0.25 | 0.724 | 3 | 6.88 |
| rente | interest | rEnt@ | Intr@st | 0.25 | 0.786 | 1.62 | 6.75 |
| respect | respect | r@spEkt | rIspEkt | 1 | 0.929 | 7 | 6.62 |
| resultaat | result | rez}ltat | rIzVlt | 0.667 | 0.838 | 5.88 | 6.75 |
| reuk | scent | r\|k | sEnt | 0.2 | 0.625 | 1.25 | 6.5 |
| reuk | smell | r\|k | smEl | 0.2 | 0.563 | 1 | 6.88 |
| reus | giant | r\|s | _2@nt | 0 | 0.69 | 1.29 | 6.75 |
| richting | direction | rIxtIN | dIrEkSH | 0.333 | 0.7 | 1.75 | 6.75 |
| ridder | knight | rId@r | n2t | 0 | 0.84 | 1.5 | 6.88 |
| riem | belt | rim | bElt | 0 | 0.625 | 1.12 | 6.88 |
| rietje | straw | ritj@ | str$ | 0 | 0.67 | 1.25 | 6.38 |
| rijst | rice | rKst | r2s | 0.4 | 0.863 | 5.25 | 7 |
| ritme | rhythm | rItm@ | rID@m | 0.333 | 0.8 | 5 | 6.88 |
| rivier | river | rivir | rIv@R | 0.833 | 0.83 | 5.88 | 7 |
| roem | fame | rum | f1m | 0 | 0.667 | 1.38 | 6.62 |
| rok | skirt | rOk | sk3t | 0 | 0.688 | 1.25 | 6.75 |
| romp | torso | rOmp | t$s5 | 0.2 | 0.613 | 2.25 | 6.38 |
| romp | trunk | rOmp | trVNk | 0.2 | 0.75 | 1.12 | 5.25 |
| rondje | circle | rOntj@ | s3kP | 0.167 | 0.617 | 1.25 | 5.62 |
| rook | smoke | rok | sm5k | 0.4 | 0.725 | 3.38 | 6.38 |
| roos | rose | ros | r5z | 0.5 | 0.884 | 5.88 | 7 |
| rouw | grief | rMw | grif | 0.2 | 0.688 | 1.38 | 5.75 |
| ruggegraat | spine | r}G@Grat | sp2n | 0.1 | 0.706 | 1.29 | 6.86 |
| rugtas | bag | NA | b{g | 0.167 | NA | 1.38 | 5.12 |
| ruig | rough | rLx | rVf | 0.4 | 0.667 | 4 | 6.5 |
| ruiken | smell | rLk@ | smEl | 0 | 0.613 | 1.38 | 6.5 |
| ruil | trade | rLl | tr1d | 0.2 | 0.738 | 1.12 | 6.88 |
| ruil | swap | rLl | swQp | 0 | 0.613 | 1 | 6.43 |
| ruil | exchange | rLl | IksJ1n_ | 0 | 0.679 | 1.12 | 5.62 |
| ruilen | trade | rLl@ | tr1d | 0.167 | 0.675 | 1 | 5.86 |
| ruilen | exchange | rLl@ | IksJ1n_ | 0.125 | 0.65 | 1.25 | 6.88 |
| ruilhandel | trade | rLlhAnd@l | tr1d | 0.3 | 0.772 | 1 | 5.88 |
| ruimte | space | rLmt@ | sp1s | 0.167 | 0.63 | 1 | 6.5 |
| ruw | raw | ryw | r$ | 0.667 | 0.75 | 2 | 5.88 |
| ruw | crude | ryw | krud | 0.4 | 0.688 | 4.38 | 4.62 |
| ruw | rough | ryw | rVf | 0.4 | 0.667 | 3.88 | 5.88 |
| ruwheid | cruelty | rywhKt | kr9ltI | 0.143 | 0.667 | 1.12 | 4.38 |
| ruzie | riot | ryzi | r2@t | 0.2 | 0.663 | 1.12 | 5.88 |
| ruzie | fight | ryzi | f2t | 0 | 0.6 | 1 | 6.62 |
| ruzie | quarrel | ryzi | kwQr@l | 0.286 | 0.667 | 3 | 5.12 |
| saai | dull | saj | dVl | 0 | 0.5 | 1.25 | 6.75 |
| saai | boring | saj | b$rIN | 0.167 | 0.6 | 1.38 | 6.38 |
| salaris | wage | salar@s | w1_ | 0.143 | 0.75 | 1.25 | 6.38 |
| sap | juice | sAp | _us | 0 | 0.667 | 1.12 | 6.88 |
| saus | sauce | sMs | s$s | 0.6 | 0.834 | 6.25 | 6.75 |
| schaamte | shame | sxamt@ | S1m | 0.625 | 0.75 | 4 | 6.62 |
| schaap | sheep | sxap | Sip | 0.5 | 0.75 | 6 | 6.88 |
| schaar | scissors | sxar | sIz@z | 0.375 | 0.65 | 2.25 | 7 |
| schandaal | scandal | sxAndal | sk{ndP | 0.778 | 0.815 | 5.5 | 6.75 |
| schatting | estimation | sxAtIN | EstIm1SH | 0.3 | 0.75 | 1.38 | 6.5 |
| schatting | estimate | sxAtIN | EstIm1t | 0.111 | 0.75 | 1.25 | 6.62 |
| schemer | dusk | sxem@r | dVsk | 0 | 0.667 | 1.25 | 6.38 |
| schemer | twilight | sxem@r | tw2l2t | 0 | 0.559 | 1.5 | 6.62 |
| schemering | dawn | sxem@rIN | d$n | 0.1 | 0.7 | 1.12 | 6.62 |
| schemering | dusk | sxem@rIN | dVsk | 0 | 0.706 | 1.5 | 6.75 |
| schemering | twilight | sxem@rIN | tw2l2t | 0 | 0.644 | 1.25 | 6.62 |
| schepping | creation | sxEpIN | kri1SH | 0.333 | 0.609 | 1.25 | 6.75 |
| schijterd | coward | sxKt@rt | k6@d | 0.333 | 0.75 | 1.12 | 6.25 |
| schildpad | turtle | sxIltpAt | t3tP | 0.111 | 0.75 | 1.25 | 7 |
| schoen | boot | sxun | but | 0.167 | 0.75 | 2.12 | 4.12 |
| schoen | shoe | sxun | Su | 0.667 | 0.813 | 5.38 | 7 |
| schoonheid | beauty | sxonhKt | bjutI | 0 | 0.672 | 1.38 | 6.88 |
| schotel | saucer | sxot@l | s$s@R | 0.286 | 0.709 | 2.5 | 5.88 |
| schoteltje | saucer | sxot@ltj@ | s$s@R | 0.2 | 0.733 | 2.5 | 6.38 |
| schouder | shoulder | sxMd@r | S5ld@R | 0.75 | 0.817 | 5.5 | 6.88 |
| schrijver | author | sxrKv@r | $T@R | 0.111 | 0.779 | 1 | 7 |
| schuld | blame | sx}lt | bl1m | 0 | 0.65 | 1.25 | 6.75 |
| schuld | guilt | sx}lt | gIlt | 0.167 | 0.78 | 1.5 | 6.62 |
| schuld | debt | sx}lt | dEt | 0 | 0.7 | 2.5 | 7 |
| seizoen | season | sKzun | sizH | 0.571 | 0.92 | 5.62 | 7 |
| sheet | sheet | NA | Sit | 1 | NA | 7 | 5.12 |
| simpelheid | simplicity | sImp@lhKt | sImplIs@tI | 0.5 | 0.845 | 5 | 6.12 |
| sjaal | shawl | Sal | S$l | 0.6 | 0.834 | 2.88 | 6 |
| sjaal | scarf | Sal | sk#f | 0.4 | 0.625 | 5.62 | 6.5 |
| slaaf | slave | slaf | sl1v | 0.6 | 0.875 | 5.12 | 6.88 |
| slachtoffer | victim | slAxtOf@r | vIktIm | 0.182 | 0.639 | 1 | 6.88 |
| slager | butcher | slaG@r | bUJ@R | 0.286 | 0.734 | 1.38 | 6.75 |
| slang | snake | slAN | sn1k | 0.4 | 0.662 | 2.5 | 7 |
| slim | smart | slIm | sm#t | 0.2 | 0.75 | 1.88 | 6.62 |
| smaak | taste | smak | t1st | 0 | 0.625 | 1.25 | 6.75 |
| smaak | flavour | smak | fl1v@R | 0.143 | 0.617 | 1.71 | 7 |
| sneeuw | snow | snew | sn5 | 0.5 | 0.85 | 5.62 | 7 |
| snoer | wire | snur | w2@R | 0.2 | 0.613 | 1.12 | 6.75 |
| snor | moustache | snOr | m@st#S | 0.111 | 0.709 | 1 | 7 |
| sok | sock | sOk | sQk | 0.75 | 0.834 | 6.25 | 6.25 |
| spanning | excitement | spAnIN | Iks2tm@nt | 0.1 | 0.722 | 1.62 | 6.38 |
| spanning | tension | spAnIN | tEnSH | 0.25 | 0.659 | 1.25 | 6.88 |
| speld | pin | spElt | pIn | 0.2 | 0.75 | 1.25 | 6.62 |
| spiegel | mirror | spiG@l | mIr@R | 0.143 | 0.7 | 1.25 | 7 |
| spijt | regret | spKt | rIgrEt | 0.167 | 0.742 | 1.12 | 6.88 |
| spoor | rail | spor | r1l | 0 | 0.688 | 1.25 | 6.75 |
| spraak | speech | sprak | spiJ | 0.333 | 0.75 | 3.25 | 6.62 |
| sprookje | tale | sprokj@ | t1l | 0.125 | 0.657 | 1.25 | 5.62 |
| staart | tail | start | t1l | 0.333 | 0.7 | 1.38 | 6.88 |
| staat | shape | stat | S1p | 0.4 | 0.625 | 3.5 | 4.38 |
| stad | town | stAt | t6n | 0 | 0.75 | 1.38 | 6.75 |
| stad | city | stAt | sItI | 0 | 0.75 | 1 | 6.62 |
| stam | trunk | stAm | trVNk | 0 | 0.65 | 1.25 | 5.25 |
| stand | mode | stAnt | m5d | 0 | 0.7 | 1.12 | 4.75 |
| stank | stench | stANk | stEnJ | 0.5 | 0.7 | 3.38 | 6.5 |
| stapel | pile | stap@l | p2l | 0.167 | 0.792 | 1.38 | 6.75 |
| steeg | alley | stex | {lI | 0.2 | 0.613 | 1.12 | 7 |
| steegje | alley | NA | {lI | 0.143 | NA | 1.25 | 7 |
| steen | rock | sten | rQk | 0 | 0.625 | 1.12 | 6.88 |
| steen | stone | sten | st5n | 0.4 | 0.913 | 5.5 | 7 |
| stem | vote | stEm | v5t | 0.25 | 0.688 | 1.5 | 6.5 |
| stem | voice | stEm | v4s | 0 | 0.688 | 1.25 | 6.88 |
| sterkte | strength | stErkt@ | strENT | 0.5 | 0.771 | 3.25 | 6 |
| steun | support | st\|n | s@p$t | 0.143 | 0.75 | 1.75 | 6.88 |
| stier | bull | stir | bUl | 0 | 0.613 | 1.38 | 6.62 |
| stilte | silence | stIlt@ | s2l@ns | 0.429 | 0.825 | 2.38 | 7 |
| stoel | chair | stul | J8R | 0 | 0.675 | 2.25 | 6.88 |
| stoer | tough | stur | tVf | 0.2 | 0.688 | 1.75 | 5.88 |
| stof | material | stOf | m@t7r7l | 0.125 | 0.679 | 2 | 6.38 |
| stof | fabric | stOf | f{brIk | 0 | 0.667 | 1.25 | 6.5 |
| stof | dust | stOf | dVst | 0 | 0.75 | 1.14 | 5.75 |
| stok | pole | stOk | p5l | 0 | 0.563 | 1.62 | 5.5 |
| straat | road | strat | r5d | 0.333 | 0.8 | 1.5 | 6.38 |
| straat | street | strat | strit | 0.667 | 0.9 | 6.12 | 7 |
| strand | beach | strAnt | biJ | 0.167 | 0.7 | 1.88 | 7 |
| strijken | iron | strKk@ | 2@n | 0.25 | 0.742 | 1.12 | 5.88 |
| stro | straw | stro | str$ | 0.6 | 0.875 | 4.38 | 6.88 |
| stroming | stream | stromIN | strim | 0.375 | 0.857 | 1.25 | 4.88 |
| stroming | flow | stromIN | fl5 | 0.125 | 0.665 | 1.25 | 5.5 |
| stroming | current | stromIN | kVr@nt | 0.25 | 0.621 | 5.38 | 5.75 |
| stroom | flood | strom | flVd | 0.333 | 0.65 | 1.5 | 6.12 |
| stroom | current | strom | kVr@nt | 0.143 | 0.641 | 2 | 6.38 |
| strootje | straw | strotj@ | str$ | 0.375 | 0.835 | 2.62 | 6.62 |
| strot | throat | strOt | Tr5t | 0.5 | 0.8 | 3.12 | 6 |
| stuk | piece | st}k | pis | 0 | 0.688 | 1.88 | 6.25 |
| suiker | sugar | sLk@r | SUg@R | 0.5 | 0.84 | 4.75 | 6.88 |
| taai | tough | taj | tVf | 0.2 | 0.667 | 3 | 6.38 |
| taak | duty | tak | djutI | 0 | 0.7 | 1.14 | 6.14 |
| taak | job | tak | _Qb | 0 | 0.65 | 1.12 | 6 |
| taak | task | tak | t#sk | 0.75 | 0.813 | 4.62 | 6.88 |
| taal | language | tal | l{NgwI_ | 0.25 | 0.715 | 1 | 6.75 |
| taart | cake | tart | k1k | 0.2 | 0.563 | 1.5 | 5.75 |
| taart | pie | tart | p2 | 0 | 0.725 | 1 | 6.62 |
| tabak | tobacco | tabAk | t@b{k5 | 0.429 | 0.792 | 4.75 | 7 |
| tafel | desk | taf@l | dEsk | 0 | 0.68 | 1.38 | 4.75 |
| tante | aunt | tAnt@ | #nt | 0.4 | 0.89 | 2.12 | 7 |
| tap | tap | tAp | t{p | 1 | 0.834 | 6.88 | 5.75 |
| tas | bag | tAs | b{g | 0.333 | 0.5 | 1.88 | 6.88 |
| taxi | cab | tAksi | k{b | 0.25 | 0.74 | 1.12 | 6.62 |
| taxi | taxi | tAksi | t{ksI | 1 | 0.84 | 6.86 | 7 |
| teken | token | tek@ | t5k@n | 0.8 | 0.88 | 1.12 | 6.88 |
| teken | sign | tek@ | s2n | 0.2 | 0.625 | 5.88 | 3.88 |
| tekort | lack | t@kOrt | l{k | 0 | 0.709 | 1.62 | 6.38 |
| tekort | shortage | t@kOrt | S$tI_ | 0.25 | 0.7 | 2.25 | 6.75 |
| testament | will | tEstamEnt | wIl | 0 | 0.717 | 1 | 6.62 |
| thee | tea | te | ti | 0.5 | 0.75 | 5.5 | 7 |
| ticket | ticket | tIk@t | tIkIt | 1 | 0.9 | 7 | 6.38 |
| tijd | time | tKt | t2m | 0.5 | 0.734 | 5.12 | 6.88 |
| tijger | tiger | tKG@r | t2g@R | 0.833 | 0.83 | 6.12 | 7 |
| toekomst | future | tukOmst | fjuJ@R | 0 | 0.672 | 1.38 | 6.88 |
| toespraak | speech | tusprak | spiJ | 0.222 | 0.75 | 2 | 6.5 |
| toevluchtsoord | refuge | tuvl}xtsort | rEfju_ | 0.143 | 0.718 | 1.12 | 6.5 |
| toevluchtsoord | haven | tuvl}xtsort | h1vH | 0.071 | 0.723 | 1.25 | 6.75 |
| tong | tongue | tON | tVN | 0.667 | 1 | 6 | 6.88 |
| toorts | torch | torts | t$J | 0.5 | 0.79 | 4.75 | 6.88 |
| touw | rope | tMw | r5p | 0.25 | 0.567 | 1.5 | 6.5 |
| traan | tear | tran | t8R | 0.4 | 0.8 | 3.86 | 6.86 |
| trein | train | trKn | tr1n | 0.8 | 0.925 | 5.88 | 7 |
| triangel | triangle | trijAN@l | tr2{NgP | 0.75 | 0.794 | 5.88 | 4.38 |
| troep | trash | trup | tr{S | 0.4 | 0.75 | 2.75 | 6.38 |
| trots | proud | trOts | pr6d | 0.4 | 0.75 | 1 | 6.75 |
| trots | pride | trOts | pr2d | 0.2 | 0.75 | 1.25 | 7 |
| tuin | garden | tLn | g#dH | 0.167 | 0.8 | 1.12 | 7 |
| tweeling | twins | twelIN | NA | 0.5 | NA | 2.62 | 7 |
| tweeling | twin | twelIN | twIn | 0.5 | 0.834 | 2.88 | 6.88 |
| uitstel | delay | LtstEl | dIl1 | 0 | 0.75 | 1.62 | 6 |
| uitverkoop | sale | Ltf@rkop | s1l | 0.1 | 0.682 | 1.12 | 6.75 |
| uitwisselen | exchange | LtwIs@l@ | IksJ1n_ | 0.091 | 0.725 | 1.5 | 6.75 |
| uitwisseling | exchange | LtwIs@lIN | IksJ1n_ | 0.083 | 0.728 | 1 | 6.88 |
| vaardigheid | ability | vard@xhKt | @bIl@tI | 0.182 | 0.689 | 1.5 | 6.62 |
| vaardigheid | skill | vard@xhKt | skIl | 0.091 | 0.661 | 1 | 6.75 |
| vaas | vase | vas | v#z | 0.5 | 0.834 | 6.12 | 7 |
| vacht | fur | vAxt | f3R | 0 | 0.688 | 1.62 | 6.75 |
| vader | dad | vad@r | d{d | 0.4 | 0.74 | 1.62 | 6.25 |
| vader | father | vad@r | f#D@R | 0.5 | 0.79 | 5.62 | 6.88 |
| varken | pig | vArk@ | pIg | 0 | 0.7 | 1.12 | 7 |
| vastbinden | pin | vAzdbInd@ | pIn | 0.2 | 0.834 | 1.62 | 5.12 |
| vel | sheet | vEl | Sit | 0.2 | 0.5 | 1 | 5.38 |
| veld | domain | vElt | d5m1n | 0 | 0.65 | 1.12 | 3.62 |
| veld | field | vElt | fild | 0.6 | 0.875 | 3.88 | 6.62 |
| verandering | change | v@rAnd@rIN | J1n_ | 0.273 | 0.755 | 1.25 | 6.5 |
| verdelen | part | v@rdel@ | p#t | 0.125 | 0.715 | 1 | 5.75 |
| verdrag | convention | v@rdrAx | k@nvEnSH | 0.2 | 0.682 | 1.38 | 6.38 |
| verdrag | treaty | v@rdrAx | tritI | 0.143 | 0.715 | 1.5 | 6.88 |
| verdriet | pain | v@rdrit | p1n | 0.125 | 0.707 | 2.38 | 6.12 |
| verdriet | sorrow | v@rdrit | sQr5 | 0.25 | 0.679 | 1.12 | 4.25 |
| verdriet | sadness | v@rdrit | s{dnIs | 0.125 | 0.672 | 1 | 6.5 |
| verdriet | grief | v@rdrit | grif | 0.375 | 0.75 | 1.38 | 6.25 |
| verf | paint | vErf | p1nt | 0 | 0.563 | 1.38 | 6.88 |
| vergadering | meeting | v@rGad@rIN | mitIN | 0.455 | 0.775 | 1.25 | 6 |
| verhaal | tale | v@rhal | t1l | 0.143 | 0.725 | 1.38 | 6.88 |
| verhaal | story | v@rhal | st$rI | 0 | 0.641 | 1.38 | 6.5 |
| verjaardag | birthday | v@rjardAx | b3Td1 | 0.3 | 0.695 | 1.38 | 7 |
| verkoop | sell | vErkop | sEl | 0.143 | 0.709 | 1.25 | 6.88 |
| verkoop | sale | vErkop | s1l | 0 | 0.625 | 1.38 | 6.25 |
| verkoudheid | flu | v@rkMthKt | flu | 0.091 | 0.767 | 1.62 | 4.62 |
| verlegen | shy | v@rleG@ | S2 | 0 | 0.7 | 1.38 | 6.88 |
| verlies | defeat | v@rlis | dIfit | 0.143 | 0.709 | 1.5 | 6 |
| verpleegster | nurse | v@rplexst@r | n3s | 0.25 | 0.75 | 1.12 | 7 |
| verraad | betrayal | v@rat | bItr1@l | 0.5 | 0.643 | 1.5 | 7 |
| verraad | treason | v@rat | trizH | 0.143 | 0.64 | 1 | 6.12 |
| verrassing | surprise | v@rAsIN | s@pr2z | 0.2 | 0.75 | 1.43 | 6.88 |
| verschil | difference | v@rsxIl | dIfr@ns | 0.1 | 0.75 | 1.38 | 7 |
| verslagen | defeat | v@rslaG@ | dIfit | 0.222 | 0.656 | 1.62 | 6.25 |
| vertraging | delay | v@rtraGIN | dIl1 | 0.2 | 0.75 | 1.75 | 6.62 |
| vertrouwen | faith | v@rtrMw@ | f1T | 0.1 | 0.731 | 1.62 | 6.62 |
| viezigheid | dirt | viz@xhKt | d3t | 0.1 | 0.744 | 1.25 | 5.88 |
| vijand | enemy | vKjAnt | En@mI | 0 | 0.7 | 1.25 | 7 |
| vinger | finger | vIN@r | fINg@R | 0.833 | 0.95 | 6.38 | 7 |
| viool | violin | vijol | v2@lIn | 0.5 | 0.75 | 5.25 | 7 |
| vlam | flame | vlAm | fl1m | 0.6 | 0.875 | 4.75 | 6.75 |
| vlees | meat | vles | mit | 0.2 | 0.563 | 1.25 | 6.5 |
| vleugel | wing | vl\|G@l | wIN | 0.143 | 0.659 | 1.62 | 6.88 |
| vlieg | fly | vlix | fl2 | 0.2 | 0.85 | 3.5 | 6.75 |
| vliegen | fly | vliG@ | fl2 | 0.143 | 0.83 | 3.25 | 6.62 |
| vliegtuig | plane | vlixtLx | pl1n | 0.111 | 0.736 | 1 | 7 |
| vliegtuig | airplane | vlixtLx | 8pl1n | 0.111 | 0.7 | 1.38 | 6 |
| vlinder | butterfly | vlInd@r | bVt@fl2 | 0.111 | 0.707 | 1.25 | 7 |
| vlo | flea | vlo | fli | 0.25 | 0.834 | 3.38 | 7 |
| vloed | flood | vlut | flVd | 0.6 | 0.875 | 5 | 6.12 |
| vloek | spell | vluk | spEl | 0 | 0.625 | 2 | 6.75 |
| vloek | curse | vluk | k3s | 0 | 0.688 | 1.5 | 6 |
| voet | foot | vut | fUt | 0.5 | 0.9 | 5.88 | 6.75 |
| vogel | bird | voG@l | b3d | 0 | 0.64 | 1.25 | 6.75 |
| volgorde | order | vOlGOrd@ | $d@R | 0.375 | 0.806 | 2.38 | 6 |
| volk | people | vOlk | pipP | 0.333 | 0.613 | 1.12 | 6.38 |
| voorbeeld | example | vorbelt | Igz#mpP | 0.111 | 0.636 | 1.25 | 6.88 |
| voordeel | advantage | vordel | @dv#ntI_ | 0.111 | 0.744 | 1.38 | 6.88 |
| voordracht | speech | vordrAxt | spiJ | 0.2 | 0.682 | 1.12 | 6.12 |
| voorhoofd | brow | vorhoft | br6 | 0.222 | 0.736 | 1.12 | 3.75 |
| voorhoofd | forehead | vorhoft | fQrId | 0.333 | 0.786 | 4.12 | 6.75 |
| voorkeur | favour | vork\|r | f1v@R | 0.25 | 0.767 | 2.25 | 6.62 |
| voorstel | proposition | vorstEl | prQp@zISH | 0.273 | 0.689 | 1.75 | 6.75 |
| voorstel | proposal | vorstEl | pr@p5zP | 0.25 | 0.707 | 1.25 | 6.75 |
| voortgang | progress | vortxAN | pr5grEs | 0.111 | 0.593 | 1.12 | 6.62 |
| vooruitgang | advancement | vorLtxAN | @dv#nsm@nt | 0.091 | 0.685 | 1.5 | 6.25 |
| vooruitgang | progress | vorLtxAN | pr5grEs | 0.091 | 0.669 | 1.43 | 6.57 |
| vordeel | favour | NA | f1v@R | 0 | NA | 1.62 | 6.25 |
| vordering | progress | vOrd@rIN | pr5grEs | 0.222 | 0.682 | 1.75 | 6.75 |
| vorm | shape | vOrm | S1p | 0 | 0.625 | 5.75 | 5.25 |
| vorm | form | vOrm | f$m | 0.75 | 0.925 | 1.12 | 6.62 |
| vos | fox | vOs | fQks | 0.333 | 0.813 | 4.75 | 7 |
| vraag | demand | vrax | dIm#nd | 0.167 | 0.625 | 1.25 | 5.62 |
| vraag | question | vrax | kwEsJ@n | 0 | 0.643 | 1 | 6.75 |
| vrede | peace | vred@ | pis | 0.2 | 0.6 | 1.5 | 7 |
| vreemd | odd | vremt | Qd | 0.167 | 0.75 | 1.12 | 6 |
| vreugde | happiness | vr\|Gd@ | h{pInIs | 0.111 | 0.6 | 1.25 | 6.38 |
| vreugde | joy | vr\|Gd@ | _4 | 0 | 0.758 | 1.25 | 6.75 |
| vriend | chap | vrint | J{p | 0 | 0.69 | 1.25 | 5.25 |
| vriend | friend | vrint | frEnd | 0.833 | 0.9 | 5.88 | 6.75 |
| vrouw | female | vrMw | fim1l | 0 | 0.65 | 1.25 | 4.75 |
| vrouw | lady | vrMw | l1dI | 0 | 0.5 | 1.5 | 6.88 |
| vrouw | dame | vrMw | d1m | 0 | 0.563 | 1.25 | 5.62 |
| vrouw | woman | vrMw | wUm@n | 0 | 0.65 | 2.12 | 7 |
| vuil | dirt | vLl | d3t | 0 | 0.567 | 1.14 | 6 |
| vuil | trash | vLl | tr{S | 0 | 0.613 | 1.25 | 6 |
| vuilnis | trash | vLlnIs | tr{S | 0 | 0.667 | 1.25 | 6.38 |
| vuist | fist | vLst | fIst | 0.6 | 0.925 | 4.12 | 7 |
| vuur | fire | vyr | f2@R | 0 | 0.838 | 3.38 | 7 |
| waard | worth | wart | w3T | 0.2 | 0.688 | 3.88 | 6.62 |
| waarde | worth | ward@ | w3T | 0.333 | 0.74 | 1.38 | 7 |
| waarde | value | ward@ | v{lju | 0.333 | 0.54 | 3.62 | 6.38 |
| waarheid | truth | warhKt | truT | 0.125 | 0.667 | 1.25 | 6.88 |
| wachter | watch | wAxt@r | wQJ | 0.429 | 0.867 | 4.38 | 5.12 |
| walvis | whale | wAlvIs | w1l | 0.333 | 0.792 | 4.88 | 7 |
| wand | wall | wAnt | w$l | 0.5 | 0.688 | 4.57 | 6.29 |
| wang | cheek | wAN | Jik | 0 | 0.549 | 1.25 | 6.5 |
| wanhoop | despair | wAnhop | dIsp8R | 0 | 0.625 | 1.25 | 6.5 |
| want | glove | wAnt | glVv | 0 | 0.5 | 1 | 5.5 |
| warmte | warmth | wArmt@ | w$mT | 0.833 | 0.75 | 1.25 | 6 |
| warmte | heat | wArmt@ | hit | 0.167 | 0.709 | 5.62 | 6.88 |
| water | water | wat@r | w$t@R | 1 | 0.89 | 6.88 | 7 |
| weg | road | wEx | r5d | 0 | 0.5 | 1.12 | 6.88 |
| wegkwijnen | pine | wExkwKn@ | p2n | 0.3 | 0.744 | 1.88 | 5.25 |
| wenkbrauw | brow | wENgbrMw | br6 | 0.333 | 0.806 | 3.14 | 5.88 |
| wens | wish | wEns | wIS | 0.25 | 0.75 | 3.12 | 6.75 |
| wereld | world | wer@lt | w3ld | 0.667 | 0.867 | 4.62 | 6.88 |
| werkster | maid | wErkst@r | m1d | 0 | 0.719 | 1.12 | 6.88 |
| werktuig | utensil | wErktLx | jutEnsP | 0.125 | 0.636 | 1 | 6.5 |
| werktuig | tool | wErktLx | tul | 0.125 | 0.715 | 1.29 | 5.62 |
| west | west | wEst | wEst | 1 | 1 | 7 | 6.88 |
| westen | west | wEst@ | wEst | 0.667 | 0.95 | 5.62 | 7 |
| wet | law | wEt | l$ | 0 | 0.584 | 1.62 | 6.88 |
| wetenschap | science | wet@sxAp | s2@ns | 0.3 | 0.744 | 1.62 | 6.5 |
| wiel | wheel | wil | wil | 0.6 | 1 | 5.38 | 6.88 |
| wijsheid | knowledge | wKshKt | nQlI_ | 0.111 | 0.65 | 1.5 | 6 |
| wijsheid | wisdom | wKshKt | wIzd@m | 0.375 | 0.784 | 3.62 | 6.62 |
| wil | will | wIl | wIl | 0.75 | 1 | 6.5 | 5.88 |
| wind | wind | wInt | wInd | 1 | 1 | 7 | 7 |
| winkel | store | wINk@l | st$R | 0.167 | 0.6 | 1.5 | 6.62 |
| winkel | shop | wINk@l | SQp | 0 | 0.625 | 1.38 | 6.25 |
| winkelen | shop | wINk@l@ | SQp | 0 | 0.643 | 1.38 | 6.25 |
| winter | winter | wInt@r | wInt@R | 1 | 0.992 | 6.88 | 7 |
| wissel | change | wIs@l | J1n_ | 0 | 0.73 | 1.38 | 5.12 |
| wisselgeld | change | wIs@lGElt | J1n_ | 0.2 | 0.739 | 1.25 | 6 |
| wisseling | chance | wIs@lIN | J#ns | 0 | 0.679 | 1.12 | 3.75 |
| woede | anger | wud@ | {Ng@R | 0 | 0.65 | 1.12 | 6.14 |
| wolf | wolf | wOlf | wUlf | 1 | 0.875 | 6 | 7 |
| wolk | cloud | wOlk | kl6d | 0.2 | 0.625 | 1.38 | 7 |
| woord | word | wort | w3d | 0.8 | 0.813 | 6 | 6.86 |
| wortel | root | wOrt@l | rut | 0.333 | 0.792 | 1.62 | 6.62 |
| wortel | carrot | wOrt@l | k{r@t | 0.167 | 0.709 | 2 | 6.88 |
| woud | woods | wMt | NA | 0.6 | NA | 1.12 | 6.38 |
| woud | wood | wMt | wUd | 0.75 | 0.9 | 5.25 | 5.88 |
| woud | forest | wMt | fQrIst | 0.167 | 0.709 | 4.5 | 5.5 |
| wraak | vengeance | wrak | vEn_@ns | 0.111 | 0.643 | 1.25 | 6.75 |
| wraak | revenge | wrak | rIvEn_ | 0 | 0.667 | 1.38 | 6.62 |
| wreed | crude | wret | krud | 0.2 | 0.75 | 1.38 | 5.38 |
| wreed | cruel | wret | kr9l | 0.4 | 0.663 | 1.12 | 6.75 |
| wreedheid | cruelty | wrethKt | kr9ltI | 0.222 | 0.729 | 1.38 | 6.75 |
| wrok | grudge | wrOk | grV_ | 0.167 | 0.75 | 1.25 | 6.62 |
| zaak | case | zak | k1s | 0.25 | 0.667 | 1.62 | 6.5 |
| zak | pocket | zAk | pQkIt | 0.167 | 0.8 | 3.62 | 6.25 |
| zak | bag | zAk | b{g | 0.333 | 0.667 | 1.12 | 5.88 |
| zal | will | NA | wIl | 0.25 | NA | 1.5 | 6.5 |
| zee | sea | ze | si | 0.333 | 0.75 | 6 | 7 |
| zeep | soap | zep | s5p | 0.25 | 0.884 | 5.62 | 6.88 |
| zeil | sail | zKl | s1l | 0.5 | 0.9 | 5.38 | 6.5 |
| zeilen | sail | zKl@ | s1l | 0.333 | 0.863 | 4.38 | 6.75 |
| zekerheid | security | zek@rhKt | sIkj9r@tI | 0.222 | 0.805 | 3 | 6.38 |
| zekerheid | certainty | zek@rhKt | s3tHtI | 0.111 | 0.738 | 2.38 | 5.88 |
| ziekenhuis | hospital | zik@hLs | hQspItP | 0 | 0.643 | 1.62 | 6.75 |
| zijde | silk | zKd@ | sIlk | 0.2 | 0.675 | 3.62 | 6.88 |
| zilver | silver | zIlv@r | sIlv@R | 0.833 | 0.992 | 6.25 | 6.88 |
| zomer | summer | zom@r | sVm@R | 0.5 | 0.89 | 5.25 | 7 |
| zon | sun | zOn | sVn | 0.333 | 1 | 6 | 7 |
| zonde | pity | zOnd@ | pItI | 0 | 0.65 | 2.12 | 5.5 |
| zonde | sin | zOnd@ | sIn | 0.2 | 0.8 | 3.25 | 6.75 |
| zondigen | sin | zOnd@G@ | sIn | 0.25 | 0.786 | 2.38 | 6.5 |
| zonsopgang | dawn | zOnsOpxAN | d$n | 0.1 | 0.772 | 1.12 | 6.62 |
| zonsopkomst | dawn | zOnsOpkOmst | d$n | 0 | 0.768 | 1.12 | 6.25 |
| zoon | son | zon | sVn | 0.5 | 0.834 | 4.5 | 6.88 |
| zorg | care | zOrx | k8R | 0.25 | 0.675 | 1.75 | 6.38 |
| zorgen | care | zOrG@ | k8R | 0.333 | 0.69 | 1.12 | 6.5 |
| zout | salt | zMt | s$lt | 0.25 | 0.813 | 4.14 | 7 |
| zullen | will | z}l@ | wIl | 0.333 | 0.724 | 1.12 | 5.62 |
| zus | sister | z}s | sIst@R | 0.167 | 0.816 | 2.88 | 6.75 |
| zuster | sister | z}st@r | sIst@R | 0.667 | 0.933 | 1.88 | 6.75 |
| zuster | nurse | z}st@r | n3s | 0.333 | 0.709 | 4.75 | 7 |
| zwaar | rough | zwar | rVf | 0 | 0.688 | 1.5 | 6 |
| zwaar | tough | zwar | tVf | 0 | 0.563 | 1.62 | 5.62 |
| zwakheid | weakness | zwAkhKt | wiknIs | 0.375 | 0.715 | 1.5 | 6.75 |
| zwakte | weakness | zwAkt@ | wiknIs | 0.375 | 0.709 | 1.75 | 6.75 |
| zweet | sweat | zwet | swEt | 0.6 | 0.875 | 5.38 | 6.88 |
| zweten | sweat | zwet@ | swEt | 0.333 | 0.85 | 4.88 | 6.88 |
