# Supplementary material for: Cross-Language Distributions of High Frequency and Phonetically Similar Cognates
Source: PLoS One. 2013 May 10;8(5):e63006. doi: 10.1371/journal.pone.0063006 (PMC3651159; doi:10.1371/journal.pone.0063006)
Supplement: Table S3 — Subjective form similarity ratings (both orthographic and phonetic similarity ratings) for 319 cognates and non-cognates. Automatic form and meaning similarity measures are added for comparison. (DOCX) [file pone.0063006.s003.docx]

Table S3. Stimulus materials from [1]

This list contains orthographic (O rating) and phonetic (P rating) similarity ratings for 319 cognates and non-cognates provided by [1]. We have added similarity scores based on automatic semantic (S), orthographic (O sim), and phonetic (P sim) similarity measures. Phonetic transcriptions (PT) were used for the computation of phonetic similarity.

1. Dijkstra T, Miwa K, Brummelhuis B, Sappelli M, Baayen H (2010) How cross-language similarity and task demands affect cognate recognition. Journal of Memory and language 62: 284–301.

Table 23. Stimulus materials from [1] with S, O, and P similarity measures.

| Dutch | English | PT | PT | S | O sim | P sim | #S | O rating | P rating |
| --- | --- | --- | --- | --- | --- | --- | --- | --- | --- |
| aap | monkey | ap | mVNkI | a | 0 | 0.504 | 1 | 1 | 1.25 |
| advies | advice | Atfis | @dv2s | a | 0.667 | 0.877 | 6 | 5.13 | 5 |
| alarm | alarm | alArm | @l#m | a | 1 | 0.797 | 2 | 7 | 7 |
| angst | fear | ANst | f7R | a | 0 | 0.5 | 5 | 1.5 | 1 |
| anker | anchor | ANk@r | {Nk@R | a | 0.5 | 0.936 | 2 | 3.63 | 4.375 |
| arend | eagle | ar@nt | igP | a | 0 | 0.492 | 2 | 1.88 | 1.375 |
| baard | beard | bart | b7d | a | 0.8 | 0.722 | 1 | 5.88 | 5.125 |
| bad | bath | bAt | b#T | a | 0.5 | 0.829 | 5 | 4.88 | 5.125 |
| bakker | baker | bAk@r | b1k@R | a | 0.833 | 0.871 | 3 | 5.75 | 4.625 |
| bal | ball | bAl | b$l | a | 0.75 | 0.9 | 9 | 6 | 6 |
| bamboe | bamboo | bAmbu | b{mbu | a | 0.833 | 0.956 | 2 | 5.75 | 5.75 |
| banaan | banana | banan | b@n#n@ | a | 0.667 | 0.814 | 2 | 5.13 | 5 |
| band | tire | bAnt | t2@R | a | 0 | 0.58 | 3 | 1 | 1 |
| bewijs | proof | b@wKs | pruf | a | 0 | 0.68 | 8 | 1 | 1 |
| boek | book | buk | bUk | a | 0.75 | 0.934 | 5 | 5.5 | 6.75 |
| boer | farmer | bur | f#m@R | a | 0.333 | 0.677 | 5 | 1.13 | 1.125 |
| boom | tree | bom | tri | a | 0 | 0.473 | 1 | 1.13 | 1 |
| boos | angry | bos | {NgrI | a | 0 | 0.468 | 4 | 1 | 1 |
| bord | plate | bOrt | pl1t | a | 0 | 0.655 | 2 | 1 | 1.375 |
| borst | breast | bOrst | brEst | a | 0.5 | 0.8 | 4 | 4 | 3.375 |
| bries | breeze | bris | briz | a | 0.5 | 1 | 1 | 4.38 | 5.75 |
| broek | pants | bruk | p{nts | a | 0 | 0.64 | 2 | 1 | 1 |
| bruid | bride | brLt | br2d | a | 0.6 | 0.95 | 2 | 4.5 | 4.375 |
| bureau | desk | byro | dEsk | a | 0.167 | 0.5 | 3 | 1 | 1 |
| chaos | chaos | xaOs | k1Qs | a | 1 | 0.7 | 4 | 7 | 5.125 |
| circus | circus | sIrk}s | s3k@s | a | 1 | 0.798 | 1 | 7 | 6.875 |
| cirkel | circle | sIrk@l | s3kP | a | 0.5 | 0.748 | 4 | 5 | 5.5 |
| code | code | kod@ | k5d | a | 1 | 0.8 | 5 | 7 | 6 |
| crisis | crisis | kris@s | kr2sIs | a | 1 | 0.913 | 2 | 7 | 5.5 |
| dak | roof | dAk | ruf | a | 0 | 0.487 | 4 | 1 | 1.125 |
| debat | debate | d@bAt | dIb1t | a | 0.833 | 0.846 | 6 | 6 | 4.75 |
| detail | detail | detAj | dit1l | a | 1 | 0.788 | 2 | 7 | 5.75 |
| dichter | poet | dIxt@r | p5It | a | 0.143 | 0.673 | 3 | 1 | 1 |
| dief | thief | dif | Tif | a | 0.6 | 0.863 | 2 | 4.75 | 5 |
| dier | animal | dir | {nImP | a | 0.167 | 0.677 | 2 | 1 | 1 |
| dij | thigh | dK | T2 | a | 0.2 | 0.694 | 2 | 1.88 | 3.125 |
| dik | thick | dIk | TIk | a | 0.4 | 0.863 | 4 | 4.88 | 5 |
| doel | target | dul | t#gIt | a | 0.167 | 0.594 | 10 | 1.13 | 1 |
| dokter | doctor | dOkt@r | dQkt@R | a | 0.667 | 0.95 | 3 | 5.88 | 6 |
| dom | silly | dOm | sIlI | a | 0 | 0.56 | 8 | 1 | 1.125 |
| domein | domain | domKn | d5m1n | a | 0.833 | 0.9 | 6 | 5.5 | 5.75 |
| dood | death | dot | dET | a | 0.2 | 0.735 | 3 | 3.75 | 3.75 |
| doof | deaf | dof | dEf | a | 0.5 | 0.873 | 1 | 4.5 | 4.75 |
| doorn | thorn | dorn | T$n | a | 0.6 | 0.722 | 2 | 3.13 | 4.125 |
| dorst | thirst | dOrst | T3st | a | 0.5 | 0.778 | 1 | 2.25 | 3.125 |
| drama | drama | drama | dr#m@ | a | 1 | 0.877 | 5 | 7 | 6.25 |
| duif | pigeon | dLf | pI_In | a | 0 | 0.668 | 1 | 1 | 1.125 |
| duim | thumb | dLm | TVm | a | 0.2 | 0.763 | 1 | 2.13 | 2.875 |
| duivel | devil | dLv@l | dEvP | a | 0.5 | 0.86 | 4 | 3.75 | 4.625 |
| dun | thin | d}n | TIn | a | 0.25 | 0.816 | 4 | 3 | 2.75 |
| echo | echo | Exo | Ek5 | a | 1 | 0.767 | 3 | 7 | 6.25 |
| eed | oath | et | 5T | a | 0 | 0.644 | 4 | 2.88 | 2.125 |
| eend | duck | ent | dVk | a | 0 | 0.534 | 1 | 1 | 1 |
| eenheid | unit | enhKt | junIt | a | 0.286 | 0.704 | 6 | 4.75 | 1.375 |
| eis | demand | Ks | dIm#nd | a | 0.167 | 0.584 | 4 | 1 | 1.125 |
| emmer | bucket | Em@r | bVkIt | a | 0.167 | 0.654 | 1 | 1.25 | 1 |
| engel | angel | EN@l | 1n_@l | a | 0.8 | 0.76 | 1 | 5.5 | 4.625 |
| enorm | huge | enOrm | hju_ | b | 0 | 0.433 | 6 | 1 | 1 |
| ezel | donkey | ez@l | dQNkI | a | 0.167 | 0.446 | 1 | 1 | 1 |
| fataal | fatal | fatal | f1tP | a | 0.833 | 0.76 | 8 | 5.88 | 5 |
| film | movie | fIlm | muvI | a | 0 | 0.636 | 4 | 1.13 | 1 |
| fles | bottle | flEs | bQtP | a | 0.167 | 0.476 | 1 | 1 | 1 |
| fout | error | fMt | Er@R | b | 0 | 0.625 | 3 | 1.25 | 1 |
| fruit | fruit | frLt | frut | a | 1 | 0.9 | 2 | 7 | 5.5 |
| gat | hole | xAt | h5l | a | 0 | 0.494 | 7 | 1 | 1 |
| gedicht | poem | x@dIxt | p5Im | a | 0 | 0.627 | 3 | 1 | 1.125 |
| geest | mind | xest | m2nd | a | 0 | 0.683 | 4 | 1 | 1 |
| gek | crazy | xEk | kr1zI | a | 0 | 0.568 | 22 | 1 | 1 |
| geld | money | xElt | mVnI | a | 0 | 0.508 | 3 | 1 | 1 |
| geloof | faith | x@lof | f1T | a | 0 | 0.637 | 4 | 1 | 1 |
| geluk | luck | x@l}k | lVk | a | 0.4 | 0.718 | 4 | 3.75 | 3.75 |
| gemak | ease | x@mAk | iz | a | 0.2 | 0.532 | 4 | 1 | 1 |
| genade | mercy | x@nad@ | m3sI | b | 0.167 | 0.642 | 9 | 1 | 1 |
| gerucht | rumor | x@r}xt | NA | a | 0.286 | ######## | 3 | 1.38 | 1.25 |
| gevaar | danger | x@var | d1n_@R | a | 0.167 | 0.657 | 6 | 1.5 | 1 |
| geval | case | x@vAl | k1s | a | 0 | 0.632 | 2 | 1 | 1 |
| geweer | rifle | x@wer | r2fP | a | 0.167 | 0.597 | 2 | 1 | 1.125 |
| gezicht | face | x@zIxt | f1s | a | 0.143 | 0.65 | 7 | 1 | 1.125 |
| gids | guide | xIts | g2d | a | 0.6 | 0.75 | 10 | 3.5 | 2.25 |
| gitaar | guitar | xitar | gIt#R | a | 0.667 | 0.82 | 1 | 5.25 | 5.125 |
| glad | smooth | xlAt | smuD | a | 0 | 0.489 | 5 | 1 | 1.125 |
| glas | glass | xlAs | gl#s | a | 0.8 | 0.875 | 3 | 6 | 5.625 |
| goud | gold | xMt | g5ld | a | 0.75 | 0.725 | 2 | 4.75 | 2.875 |
| graad | degree | xrat | dIgri | a | 0.167 | 0.5 | 6 | 2.13 | 1.375 |
| graf | grave | xrAf | gr1v | a | 0.6 | 0.763 | 3 | 5.13 | 3.625 |
| grap | joke | xrAp | _5k | a | 0 | 0.522 | 9 | 1 | 1.125 |
| grens | limit | xrEns | lImIt | b | 0 | 0.557 | 3 | 1.25 | 1 |
| grijs | grey | xrKs | gr1 | a | 0.4 | 0.725 | 4 | 3 | 2.875 |
| groen | green | xrun | grin | a | 0.8 | 0.85 | 8 | 5.5 | 3.875 |
| groot | large | xrot | l#_ | a | 0 | 0.625 | 5 | 1 | 1 |
| grot | cave | xrOt | k1v | a | 0 | 0.578 | 3 | 1.13 | 1.125 |
| gunst | favour | x}nst | f1v@R | a | 0 | 0.575 | 4 | 1 | 1.125 |
| hard | hard | hArt | h#d | a | 1 | 0.85 | 9 | 7 | 6.375 |
| haven | port | hav@ | p$t | a | 0 | 0.462 | 1 | 1 | 1 |
| heks | witch | hEks | wIJ | a | 0 | 0.52 | 2 | 1.25 | 1.5 |
| hel | hell | hEl | hEl | a | 0.75 | 1 | 2 | 5.75 | 7 |
| helder | bright | hEld@r | br2t | b | 0 | 0.592 | 5 | 1 | 1.25 |
| hemel | heaven | hem@l | hEvH | a | 0.5 | 0.635 | 4 | 2.63 | 2.25 |
| herfst | autumn | hErfst | $t@m | a | 0 | 0.598 | 2 | 1 | 1.125 |
| hertog | duke | hErtOx | djuk | a | 0 | 0.545 | 1 | 1 | 1 |
| hoek | angle | huk | {NgP | a | 0 | 0.535 | 1 | 1 | 1 |
| honger | hunger | hON@r | hVNg@R | b | 0.833 | 0.9 | 3 | 5.88 | 5 |
| honing | honey | honIN | hVnI | a | 0.5 | 0.84 | 1 | 3.25 | 4.25 |
| hoofd | head | hoft | hEd | a | 0.4 | 0.78 | 7 | 3.88 | 2.625 |
| hoop | hope | hop | h5p | a | 0.5 | 0.9 | 1 | 5 | 6.5 |
| horloge | watch | hOrloZ@ | wQJ | a | 0 | 0.597 | 1 | 1 | 1 |
| hotel | hotel | hotEl | h5tEl | a | 1 | 0.94 | 1 | 7 | 6.875 |
| huur | rent | hyr | rEnt | a | 0 | 0.567 | 3 | 1 | 1 |
| huurder | tenant | hyrd@r | tEn@nt | a | 0 | 0.6 | 2 | 1 | 1.25 |
| idee | idea | ide | 2d7 | a | 0.75 | 0.8 | 13 | 5.63 | 4.5 |
| idioot | idiot | idijot | Id7t | a | 0.833 | 0.75 | 10 | 5.75 | 5 |
| jaar | year | jar | j7R | a | 0.5 | 0.763 | 6 | 4.75 | 4.125 |
| jas | coat | jAs | k5t | a | 0.25 | 0.493 | 3 | 1 | 1 |
| jeugd | youth | j\|xt | juT | a | 0.2 | 0.705 | 4 | 3 | 3.125 |
| jeuk | itch | j\|k | IJ | a | 0 | 0.634 | 2 | 1 | 1 |
| jury | jury | Zyri | _9rI | a | 1 | 0.794 | 2 | 6.88 | 5.75 |
| juweel | jewel | jywel | _u@l | a | 0.667 | 0.735 | 4 | 4.5 | 4.375 |
| kaart | card | kart | k#d | a | 0.4 | 0.825 | 3 | 3.75 | 4.375 |
| kaas | cheese | kas | Jiz | a | 0.167 | 0.667 | 1 | 1.88 | 2.125 |
| kamer | room | kam@r | rum | a | 0 | 0.602 | 3 | 1.13 | 1 |
| kans | chance | kAns | J#ns | a | 0.333 | 0.875 | 3 | 3.75 | 3.5 |
| kantoor | office | kAntor | QfIs | a | 0 | 0.686 | 4 | 1 | 1 |
| keel | throat | kel | Tr5t | a | 0 | 0.533 | 2 | 1 | 1 |
| kelder | cellar | kEld@r | sEl@R | a | 0.5 | 0.817 | 4 | 3.13 | 3.125 |
| kerk | church | kErk | J3J | a | 0.167 | 0.65 | 3 | 1.5 | 1.5 |
| kern | core | kErn | k$R | a | 0.25 | 0.775 | 3 | 2.63 | 2.875 |
| kers | cherry | kErs | JErI | a | 0.333 | 0.669 | 4 | 1.25 | 2 |
| keten | chain | ket@ | J1n | a | 0.2 | 0.7 | 10 | 1.63 | 2.5 |
| keuze | choice | k\|z@ | J4s | a | 0.167 | 0.7 | 6 | 1.75 | 1.125 |
| klein | small | klKn | sm$l | a | 0 | 0.465 | 3 | 1.13 | 1 |
| kleur | colour | kl\|r | kVl@R | a | 0.5 | 0.832 | 4 | 2 | 3 |
| klok | clock | klOk | klQk | a | 0.6 | 0.95 | 4 | 5.75 | 6.875 |
| koel | cool | kul | kul | a | 0.5 | 1 | 5 | 4.13 | 6.25 |
| koffie | coffee | kOfi | kQfI | a | 0.667 | 0.9 | 2 | 4.5 | 6.75 |
| kogel | bullet | koG@l | bUlIt | a | 0.167 | 0.554 | 2 | 1 | 1.125 |
| konijn | rabbit | konKn | r{bIt | a | 0 | 0.713 | 1 | 1.25 | 1 |
| koning | king | konIN | kIN | a | 0.667 | 0.8 | 4 | 3.88 | 3 |
| kooi | cage | koj | k1_ | a | 0 | 0.739 | 2 | 1.63 | 2.375 |
| koor | choir | kor | kw2@R | a | 0.4 | 0.732 | 8 | 4.25 | 4.375 |
| koord | cord | kort | k$d | a | 0.6 | 0.825 | 3 | 4.38 | 6.375 |
| koorts | fever | korts | fiv@R | a | 0 | 0.527 | 3 | 1 | 1 |
| kort | short | kOrt | S$t | a | 0.6 | 0.738 | 5 | 4.13 | 4.75 |
| kroon | crown | kron | kr6n | a | 0.6 | 0.925 | 3 | 4.5 | 4.75 |
| kruid | herb | krLt | h3b | a | 0 | 0.6 | 2 | 1.25 | 1 |
| kurk | cork | k}rk | k$k | a | 0.5 | 0.747 | 2 | 3.63 | 4.375 |
| kus | kiss | k}s | kIs | a | 0.5 | 0.953 | 3 | 4.38 | 4.625 |
| kussen | pillow | k}s@ | pIl5 | a | 0 | 0.686 | 2 | 1 | 1 |
| laars | boot | lars | but | a | 0 | 0.485 | 1 | 1.63 | 1 |
| lam | lamb | lAm | l{m | a | 0.75 | 0.926 | 1 | 5.88 | 4.5 |
| lamp | lamp | lAmp | l{mp | a | 1 | 0.944 | 2 | 7 | 5.625 |
| last | burden | lAst | b3dH | a | 0 | 0.587 | 3 | 1 | 1 |
| lawaai | noise | lawaj | n4z | a | 0 | 0.53 | 5 | 1.13 | 1.25 |
| leeg | empty | lex | EmptI | a | 0 | 0.44 | 7 | 1 | 1 |
| leeuw | lion | lew | l2@n | a | 0.2 | 0.669 | 1 | 2.38 | 3.125 |
| leger | army | leG@r | #mI | a | 0 | 0.532 | 4 | 1 | 1 |
| leider | leader | lKd@r | lid@R | a | 0.833 | 0.94 | 7 | 5.63 | 4.625 |
| lelijk | ugly | lel@k | VglI | a | 0.167 | 0.579 | 2 | 1 | 1.125 |
| lengte | length | lENt@ | lENT | a | 0.833 | 0.818 | 1 | 5.63 | 4.875 |
| lepel | spoon | lep@l | spun | a | 0 | 0.632 | 1 | 1 | 1 |
| lichaam | body | lIxam | bQdI | a | 0 | 0.457 | 5 | 1 | 1 |
| licht | light | lIxt | l2t | a | 0.8 | 0.85 | 14 | 5.5 | 4.5 |
| lid | member | lIt | mEmb@R | b | 0 | 0.558 | 3 | 1 | 1 |
| lied | song | lit | sQN | a | 0 | 0.513 | 4 | 1.38 | 1.125 |
| liefde | love | livd@ | lVv | a | 0.333 | 0.691 | 4 | 2.88 | 2.25 |
| lijm | glue | lKm | glu | a | 0 | 0.534 | 6 | 1.38 | 1.125 |
| logica | logic | loGika | lQ_Ik | a | 0.833 | 0.734 | 3 | 5.75 | 4.5 |
| lui | lazy | lL | l1zI | a | 0.25 | 0.7 | 2 | 1.38 | 2.25 |
| maagd | virgin | maxt | v3_In | a | 0.167 | 0.692 | 1 | 1 | 1 |
| maan | moon | man | mun | a | 0.5 | 0.789 | 1 | 4.88 | 4.375 |
| maand | month | mant | mVnT | a | 0.2 | 0.801 | 1 | 3.63 | 3.25 |
| macht | power | mAxt | p6@R | a | 0 | 0.575 | 8 | 1.38 | 1 |
| masker | mask | mAsk@r | m#sk | a | 0.667 | 0.817 | 3 | 5.63 | 3.876 |
| massa | mass | mAsa | m{s | NA | 0.8 | 0.819 | 1 | 6.13 | 4.875 |
| meisje | girl | mKsj@ | g3l | a | 0.167 | 0.555 | 2 | 1 | 1 |
| melk | milk | mElk | mIlk | a | 0.75 | 0.925 | 3 | 6 | 4.625 |
| meloen | melon | m@lun | mEl@n | a | 0.833 | 0.889 | 1 | 5.38 | 4.5 |
| menigte | crowd | men@xt@ | kr6d | a | 0 | 0.639 | 4 | 1 | 1 |
| menu | menu | meny | mEnju | a | 1 | 0.8 | 4 | 7 | 5.875 |
| mes | knife | mEs | n2f | a | 0 | 0.734 | 1 | 1 | 1 |
| metaal | metal | metal | mEtP | a | 0.833 | 0.82 | 1 | 6 | 4.875 |
| mild | mild | mIlt | m2ld | a | 1 | 0.975 | 6 | 7 | 6 |
| misdaad | crime | mIzdat | kr2m | a | 0 | 0.631 | 8 | 1 | 1 |
| model | model | modEl | mQdP | a | 1 | 0.78 | 15 | 7 | 5.75 |
| moeras | swamp | murAs | swQmp | a | 0 | 0.531 | 4 | 1 | 1.125 |
| molen | mill | mol@ | mIl | a | 0.4 | 0.794 | 1 | 3.38 | 2.375 |
| moment | moment | momEnt | m5m@nt | a | 1 | 0.927 | 7 | 7 | 6 |
| motor | engine | motOr | En_In | a | 0 | 0.628 | 2 | 1 | 1 |
| mouw | sleeve | mMw | sliv | a | 0 | 0.613 | 1 | 1 | 1.125 |
| muis | mouse | mLs | m6s | a | 0.4 | 0.892 | 2 | 3.5 | 4.5 |
| munt | coin | m}nt | k4n | a | 0 | 0.663 | 8 | 1.38 | 1.125 |
| muur | wall | myr | w$l | a | 0 | 0.515 | 5 | 1 | 1.125 |
| mythe | myth | mite | mIT | a | 0.8 | 0.722 | 2 | 5.88 | 4.25 |
| naald | needle | nalt | nidP | a | 0.333 | 0.6 | 7 | 3.13 | 3.25 |
| nest | nest | nEst | nEst | a | 1 | 1 | 1 | 7 | 7 |
| neus | nose | n\|s | n5z | a | 0.25 | 0.9 | 2 | 3.25 | 4.75 |
| noodlot | fate | nodlOt | f1t | c | 0 | 0.634 | 5 | 1 | 1 |
| oma | granny | oma | gr{nI | a | 0.167 | 0.528 | 3 | 1 | 1 |
| ontwerp | design | OntwErp | dIz2n | b | 0 | 0.618 | 4 | 1.13 | 1 |
| oom | uncle | om | VNkP | a | 0 | 0.523 | 1 | 1 | 1.5 |
| oorzaak | cause | orzak | k$z | a | 0 | 0.572 | 4 | 1 | 1 |
| oost | east | ost | ist | a | 0.5 | 0.906 | 3 | 4.38 | 4.375 |
| oven | oven | ov@ | VvH | a | 1 | 0.606 | 4 | 7 | 6.125 |
| paard | horse | part | h$s | a | 0 | 0.508 | 2 | 1.13 | 1.125 |
| paleis | palace | palKs | p{lIs | a | 0.5 | 0.92 | 1 | 4.63 | 5 |
| parel | pearl | par@l | p3l | a | 0.6 | 0.76 | 1 | 4.63 | 3.5 |
| pijl | arrow | pKl | {r5 | a | 0 | 0.439 | 1 | 1 | 1 |
| pijn | pain | pKn | p1n | a | 0.5 | 0.934 | 2 | 4.5 | 5.375 |
| pil | pill | pIl | pIl | a | 0.75 | 1 | 4 | 6 | 6.75 |
| plan | plan | plAn | pl{n | a | 1 | 0.944 | 10 | 7 | 6.125 |
| plant | plant | plAnt | pl#nt | a | 1 | 0.98 | 2 | 7 | 6.25 |
| plicht | duty | plIxt | djutI | a | 0 | 0.612 | 2 | 1 | 1 |
| poort | gate | port | g1t | a | 0 | 0.625 | 3 | 1 | 1.5 |
| post | mail | pOst | m1l | a | 0 | 0.675 | 4 | 1.13 | 1 |
| prijs | price | prKs | pr2s | a | 0.6 | 0.95 | 1 | 4.25 | 5.375 |
| prins | prince | prIns | prIns | a | 0.667 | 1 | 1 | 5.13 | 6.75 |
| punt | point | p}nt | p4nt | a | 0.6 | 0.94 | 5 | 4.75 | 4.625 |
| puur | pure | pyr | pj9R | a | 0.5 | 0.744 | 5 | 4.88 | 5.25 |
| raam | window | ram | wInd5 | a | 0 | 0.52 | 2 | 1 | 1.125 |
| rechter | judge | rExt@r | _V_ | a | 0.143 | 0.66 | 1 | 1 | 1 |
| regen | rain | reG@ | r1n | a | 0.4 | 0.719 | 2 | 3.75 | 2.875 |
| reis | voyage | rKs | v4I_ | b | 0 | 0.73 | 1 | 1 | 1 |
| ridder | knight | rId@r | n2t | a | 0 | 0.76 | 1 | 1.13 | 1.125 |
| rijk | rich | rKk | rIJ | a | 0.5 | 0.8 | 3 | 3 | 3.5 |
| ring | ring | rIN | rIN | a | 1 | 1 | 6 | 7 | 6.75 |
| ritme | rhythm | rItm@ | rID@m | a | 0.333 | 0.728 | 2 | 4 | 4.25 |
| rok | skirt | rOk | sk3t | a | 0 | 0.7 | 1 | 1.13 | 1.125 |
| saai | dull | saj | dVl | a | 0 | 0.634 | 3 | 1 | 1 |
| sap | juice | sAp | _us | a | 0 | 0.567 | 2 | 1.13 | 1 |
| scherm | screen | sxErm | skrin | a | 0.5 | 0.66 | 7 | 2.75 | 2.125 |
| schip | ship | sxIp | SIp | a | 0.8 | 0.85 | 2 | 5.5 | 5.125 |
| schoen | shoe | sxun | Su | a | 0.667 | 0.725 | 2 | 4.38 | 4 |
| school | school | sxol | skul | b | 1 | 0.85 | 7 | 7 | 5.625 |
| schroef | screw | sxruf | skru | a | 0.571 | 0.82 | 4 | 1.88 | 2.25 |
| schuld | debt | sx}lt | dEt | a | 0 | 0.638 | 1 | 1.25 | 2.75 |
| schuld | guilt | sx}lt | gIlt | a | 0.167 | 0.792 | 2 | 1.25 | 1 |
| servet | napkin | sErvEt | n{pkIn | a | 0 | 0.7 | 1 | 1.13 | 1 |
| sneeuw | snow | snew | sn5 | a | 0.5 | 0.8 | 2 | 4.5 | 4.125 |
| snel | fast | snEl | f#st | a | 0 | 0.525 | 7 | 1.13 | 1.25 |
| snoep | candy | snup | k{ndI | a | 0 | 0.556 | 6 | 1 | 1 |
| soep | soup | sup | sup | a | 0.75 | 1 | 1 | 5.5 | 6.5 |
| sok | sock | sOk | sQk | a | 0.75 | 0.934 | 1 | 5.75 | 6.75 |
| spiegel | mirror | spiG@l | mIr@R | a | 0.143 | 0.705 | 4 | 1 | 1 |
| spier | muscle | spir | mVsP | a | 0.167 | 0.538 | 1 | 1 | 1 |
| spijt | regret | spKt | rIgrEt | a | 0.167 | 0.723 | 2 | 1.13 | 1.125 |
| spoor | rail | spor | r1l | c | 0 | 0.598 | 3 | 1.25 | 1.125 |
| spoor | trace | spor | tr1s | a | 0 | 0.66 | 3 | 1 | 1 |
| sport | sport | spOrt | sp$t | a | 1 | 0.88 | 3 | 7 | 6.5 |
| steeg | alley | stex | {lI | a | 0.2 | 0.496 | 2 | 1.13 | 1 |
| steen | stone | sten | st5n | a | 0.4 | 0.925 | 8 | 4.75 | 4.375 |
| stem | voice | stEm | v4s | a | 0 | 0.644 | 5 | 1 | 1.125 |
| stem | vote | stEm | v5t | a | 0.25 | 0.665 | 2 | 1.25 | 1 |
| sterk | strong | stErk | strQN | a | 0.333 | 0.772 | 7 | 3.5 | 2.5 |
| stier | bull | stir | bUl | a | 0 | 0.592 | 2 | 1 | 1.125 |
| stoel | chair | stul | J8R | a | 0 | 0.592 | 2 | 1 | 1.25 |
| storm | storm | stOrm | st$m | a | 1 | 0.88 | 2 | 7 | 7 |
| straat | street | strat | strit | a | 0.667 | 0.88 | 2 | 5.5 | 4.5 |
| strand | beach | strAnt | biJ | a | 0.167 | 0.562 | 2 | 1 | 1.125 |
| stuk | piece | st}k | pis | a | 0 | 0.625 | 14 | 1 | 1.125 |
| suiker | sugar | sLk@r | SUg@R | a | 0.5 | 0.896 | 1 | 3.5 | 3.5 |
| tak | branch | tAk | br#nJ | a | 0.167 | 0.677 | 4 | 1.13 | 1.625 |
| tand | tooth | tAnt | tuT | a | 0.2 | 0.621 | 4 | 3.88 | 2.875 |
| teder | tender | ted@r | tEnd@R | a | 0.833 | 0.85 | 4 | 5.88 | 3.375 |
| teken | sign | tek@ | s2n | a | 0.2 | 0.621 | 11 | 1.13 | 1 |
| tennis | tennis | tEn@s | tEnIs | a | 1 | 0.956 | 2 | 7 | 6.875 |
| tomaat | tomato | tomat | t@m#t5 | a | 0.667 | 0.843 | 1 | 5.5 | 4.25 |
| tong | tongue | tON | tVN | a | 0.667 | 1 | 3 | 4.88 | 5.625 |
| toren | tower | tor@ | t6@R | a | 0.6 | 0.675 | 1 | 3.63 | 3 |
| totaal | total | total | t5tP | a | 0.833 | 0.82 | 9 | 5.38 | 5.75 |
| trein | train | trKn | tr1n | a | 0.8 | 0.95 | 3 | 5.5 | 5 |
| troon | throne | tron | Tr5n | a | 0.5 | 0.822 | 1 | 4.63 | 5.75 |
| tuin | garden | tLn | g#dH | a | 0.167 | 0.655 | 2 | 1.13 | 1 |
| twijfel | doubt | twKf@l | d6t | a | 0 | 0.622 | 2 | 1.13 | 1 |
| type | type | tip@ | t2p | a | 1 | 0.8 | 8 | 7 | 5.125 |
| uur | hour | yr | 6@R | a | 0.5 | 0.662 | 3 | 2.88 | 3.75 |
| vallei | valley | vAlK | v{lI | a | 0.833 | 0.894 | 2 | 5.38 | 4.625 |
| vee | cattle | ve | k{tP | a | 0.167 | 0.49 | 3 | 1 | 1.125 |
| verdrag | treaty | v@rdrAx | tritI | a | 0.143 | 0.574 | 3 | 1 | 1 |
| verf | paint | vErf | p1nt | a | 0 | 0.685 | 1 | 1 | 1 |
| verhaal | story | v@rhal | st$rI | a | 0 | 0.577 | 7 | 1.13 | 1.125 |
| verhaal | tale | v@rhal | t1l | b | 0.143 | 0.635 | 2 | 1 | 1 |
| verlies | loss | v@rlis | lQs | a | 0.286 | 0.645 | 6 | 1.75 | 1.375 |
| vijand | enemy | vKjAnt | En@mI | a | 0 | 0.572 | 2 | 1 | 1 |
| vleugel | wing | vl\|G@l | wIN | a | 0.143 | 0.627 | 8 | 1 | 1.25 |
| vloed | flood | vlut | flVd | a | 0.6 | 0.875 | 7 | 2.75 | 5.125 |
| voet | foot | vut | fUt | a | 0.5 | 0.934 | 8 | 3.13 | 5.75 |
| vogel | bird | voG@l | b3d | a | 0 | 0.573 | 1 | 1 | 1 |
| vonk | spark | vONk | sp#k | a | 0.2 | 0.625 | 3 | 1.38 | 1.125 |
| vorm | shape | vOrm | S1p | a | 0 | 0.68 | 5 | 1 | 1 |
| vrede | peace | vred@ | pis | a | 0.2 | 0.608 | 2 | 1.13 | 1 |
| vrouw | wife | vrMw | w2f | a | 0 | 0.683 | 2 | 1.63 | 1.125 |
| vrouw | woman | vrMw | wUm@n | a | 0 | 0.592 | 1 | 1.63 | 1.125 |
| vuil | dirt | vLl | d3t | NA | 0 | 0.515 | 1 | 1 | 1.125 |
| vuist | fist | vLst | fIst | a | 0.6 | 0.95 | 2 | 3.38 | 3.75 |
| vuur | fire | vyr | f2@R | a | 0 | 0.775 | 8 | 1.5 | 2.875 |
| warmte | warmth | wArmt@ | w$mT | a | 0.833 | 0.715 | 2 | 5.88 | 4.875 |
| water | water | wat@r | w$t@R | a | 1 | 0.924 | 6 | 7 | 6.125 |
| wees | orphan | wes | $fH | a | 0 | 0.506 | 3 | 1 | 1 |
| weg | road | wEx | r5d | a | 0 | 0.559 | 3 | 1.25 | 1 |
| wiel | wheel | wil | wil | a | 0.6 | 1 | 1 | 4.13 | 6.875 |
| wild | wild | wIlt | w2ld | a | 1 | 0.975 | 9 | 7 | 5.5 |
| wind | wind | wInt | wInd | a | 1 | 1 | 4 | 7 | 6.625 |
| winkel | shop | wINk@l | SQp | a | 0 | 0.505 | 4 | 1.13 | 1 |
| winter | winter | wInt@r | wInt@R | a | 1 | 0.984 | 1 | 7 | 7 |
| wond | wound | wOnt | wund | a | 0.8 | 0.875 | 2 | 5.25 | 5.25 |
| wortel | carrot | wOrt@l | k{r@t | a | 0.167 | 0.62 | 4 | 1.13 | 1.125 |
| zaad | seed | zat | sid | a | 0.25 | 0.8 | 2 | 2.88 | 3.875 |
| zacht | soft | zAxt | sQft | a | 0.2 | 0.875 | 10 | 1.5 | 2.25 |
| zadel | saddle | zad@l | s{dP | a | 0.5 | 0.84 | 2 | 3.75 | 4.25 |
| zak | pocket | zAk | pQkIt | a | 0.167 | 0.7 | 1 | 1 | 1.25 |
| zeep | soap | zep | s5p | a | 0.25 | 0.9 | 1 | 3 | 4.125 |
| zeker | sure | zek@r | S$R | a | 0.2 | 0.712 | 1 | 1.13 | 1.75 |
| zijde | silk | zKd@ | sIlk | a | 0.2 | 0.625 | 1 | 1 | 2.125 |
| zomer | summer | zom@r | sVm@R | a | 0.5 | 0.92 | 1 | 4.13 | 5 |
| zout | salt | zMt | s$lt | a | 0.25 | 0.755 | 8 | 2.13 | 3.125 |
| zuid | south | zLt | s6T | a | 0.2 | 0.755 | 3 | 2.25 | 3.125 |
| zuur | acid | zyr | {sId | b | 0 | 0.775 | 3 | 1 | 1 |
| zwaar | heavy | zwar | hEvI | a | 0.2 | 0.455 | 6 | 1.25 | 1 |
| zwaard | sword | zwart | s$d | a | 0.5 | 0.744 | 3 | 4.25 | 3.125 |
